# Characterization of intestinal immune responses in generalized human and murine lipodystrophy

**DOI:** 10.1172/JCI192322

**Published:** 2026-03-16

**Authors:** Marilena Letizia, Toka Omar, Patrick Weidner, Manuel O. Jakob, Inka Freise, Susanne M. Krug, Britt-Sabina Löscher, Elisa Rosati, Benedikt Obermayer, Reyes Gamez-Belmonte, Julia Hecker, Jörn-Felix Ziegler, Benjamin Weixler, Patrick Asbach, Desiree Kunkel, Michael Stumvoll, Konstanze Miehle, Christoph Becker, Christoph S.N. Klose, Rainer Glauben, Dieter Beule, Anja A. Kühl, Thomas Conrad, Frank Tacke, Stefan Wirtz, Andre Franke, Ashley D. Sanders, Britta Siegmund, Carl Weidinger

**Affiliations:** 1 Charité – Universitätsmedizin Berlin, corporate member of Freie Universität Berlin, Humboldt-Universität zu Berlin, Berlin, Germany.; 2 Department of Gastroenterology, Infectious Diseases and Rheumatology, Campus Benjamin Franklin, Berlin, Germany.; 3 Berlin Institute for Medical Systems Biology, Max Delbrück Center for Molecular Medicine in the Helmholtz Association, Berlin, Germany.; 4 Berlin Institute of Health (BIH) at Charité – Universitätsmedizin Berlin, Berlin, Germany.; 5 Department of Microbiology, Infectious Diseases and Immunology,; 6 Institute of Clinical Physiology/Nutritional Medicine, and; 7 Institute of Clinical Molecular Biology, Kiel University and University Medical Center Schleswig-Holstein, Kiel, Germany.; 8 Core Unit Bioinformatics, Berlin Institute of Health, Berlin, Germany.; 9 Department of Medicine 1, Universitätsklinikum Erlangen, Friedrich-Alexander-Universität Erlangen-Nürnberg, Erlangen, Germany.; 10 Department of Visceral Surgery and; 11 Department of Radiology, Campus Benjamin Franklin, Berlin, Germany.; 12 Berlin Institute of Health at Charité – Universitätsmedizin Berlin, Flow and Mass Cytometry Core Facility, Berlin, Germany.; 13 Medical Department III – Endocrinology, Nephrology, Rheumatology, University of Leipzig Medical Center, Germany, Leipzig, German.; 14 Cluster of Excellence ImmunoPreCept, Charité – Universitätsmedizin Berlin, Berlin, Germany.; 15 iPATH.Berlin–Immunopathology for Experimental Models, Core Facility of the Charité, Berlin, Germany.; 16 Core Unit Genomics, Berlin Institute of Health at Charité – Universitätsmedizin Berlin, Berlin, Germany.; 17 Department of Hepatology and Gastroenterology, Campus Virchow-Klinikum and Campus Charité Mitte, Berlin, Germany.

**Keywords:** Autoimmunity, Endocrinology, Gastroenterology, Adipose tissue, Inflammatory bowel disease, T cells

## Abstract

Acquired generalized lipodystrophy (AGL) is a rare metabolic disorder frequently associated with autoimmunity. Its etiology is incompletely understood, and the effect of adipose tissue loss on intestinal inflammation in AGL remains unclear. Using mass cytometry and single-cell RNA-seq, we observed an oligoclonal expansion of T cells in the periphery and inflamed intestine in a patient with AGL and Crohn’s disease (AGLCD). To explore if loss of adipose tissue triggers lymphoproliferation, we studied lipodystrophic mice as a model for AGL. Unexpectedly, lipodystrophic mice did not show T cell expansion, were protected from colitis, and displayed a defect in the development of proinflammatory T cells, which could be reversed by allogeneic fat transplantations, indicating that clonal T cell expansion in AGLCD is not primarily caused by lipodystrophy. Instead, gene sequencing revealed a T cell–intrinsic de novo neuroblastoma RAS viral oncogene homolog (*NRAS*) mutation, implicating somatic mosaicism as a facilitator of clonal T cell expansion and intestinal inflammation in AGLCD.

## Abstract 2

This study shows that fat loss protects mice from intestinal inflammation, whereas in a patient with acquired generalized lipodystrophy and Crohn’s disease, inflammation is driven by T cell-intrinsic lymphoproliferation.

## Graphical abstract

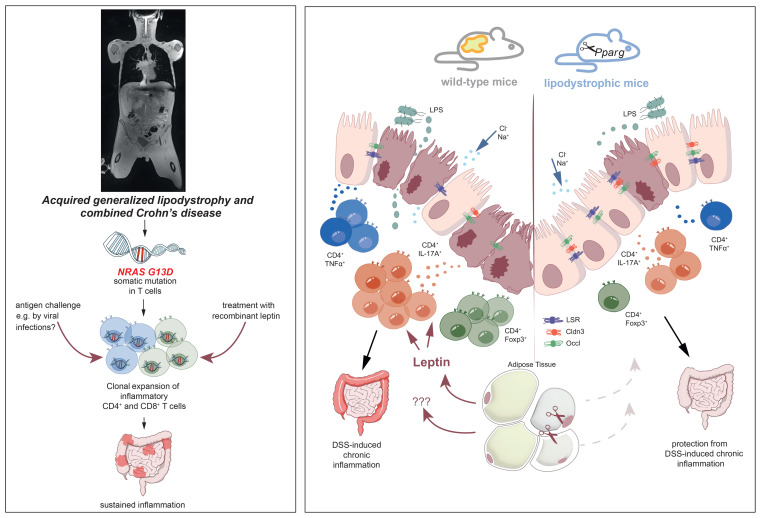

## Introduction

Acquired generalized lipodystrophy (AGL) is a complex metabolic disorder of nonhereditary origin. It is characterized by the complete absence of adipose tissue, severe type 2 diabetes, and early onset of metabolic complications including the development of pronounced steatohepatitis (1, 2). AGL is frequently associated with autoimmune and inflammatory comorbidities, including autoimmune hepatitis, arthritis, glomerulonephritis, and Crohn’s disease (CD). Additionally, patients with AGL are at an elevated risk of developing rare forms of T cell lymphoma (3).

The etiology of AGL remains poorly understood. However, initial observations in patients with melanoma suggest a T cell–driven development of AGL, since several cases of progressive loss of adipose tissue and AGL have been reported in patients with cancer following immunomodulatory therapies with the checkpoint blockers nivolumab or pembrolizumab (4–6). The high incidence of T cell lymphoma occurring in patients with AGL further supports the hypothesis that T cells may play a critical role in AGL pathogenesis (3). Nevertheless, it remains unclear whether the increased incidence of autoinflammatory disorders in patients with AGL directly results from the absence of fat-derived immunomodulatory signals or whether inflammation is primarily caused by T cell–intrinsic disturbances of immune cell homeostasis. Additionally, the precise manner by which the absence of adipose tissue and missing fat-derived signals influences inflammation in AGL is not understood.

In addition to the clinical necessity of elucidating the pathophysiology of AGL, human lipodystrophies are of particular interest for metabolic research because they can serve as a powerful model system to study the interplay between adipose tissue and the immune system. To date, the role of adipose tissue in the pathophysiology of inflammatory bowel diseases (IBD) such as CD remains elusive. However, it is well established that patients with CD commonly exhibit specific alterations in the mesenteric fat, also referred to as “creeping fat” (CF). These changes include the development of hyperplastic adipocytes, increased local production of various adipokines including leptin and adiponectin, infiltration of lymphocytes and macrophages, as well as colonization of CF with live bacteria (7, 8). Furthermore, recent epidemiologic meta-analyses have indicated that there may be an association between obesity and an increased risk of developing CD (9). There is also evidence that obesity is linked to a worse clinical outcome in patients with CD, comprising an elevated risk of disease recurrence, therapeutic failure, and higher resection rates (10). However, it remains challenging to decipher the causative contribution of individual adipose tissue–derived signals to the pathogenesis of IBD in humans.

By studying a patient with AGL and concomitant Crohn’s disease (AGLCD), we previously observed that treatment with recombinant leptin, administered to improve leptin deficiency–mediated type 2 diabetes, altered immune cell function as well as the metabolism of macrophages and T cells. Thus, leptin substitution induced TNF-α in T cells and monocytes in vivo and worsened intestinal inflammation in the patient with AGLCD, suggesting that leptin acts as a proinflammatory adipokine in human intestinal inflammation (11). Similarly, previous data indicate that leptin receptor–deficient (LEPR-deficient) CD4^+^ T cells of *Lepr^fl/fl^ Cd4-Cre* mice fail to induce intestinal and neuronal inflammation in T cell transfer models (12, 13), as LEPR-deficient mice show impaired Th17 cell differentiation in vitro and in vivo (13), underscoring leptin’s proinflammatory role in IBD pathogenesis.

In this study, we aimed to elucidate the molecular mechanisms contributing to intestinal inflammation in AGL. We therefore characterized a rare patient with AGLCD and used lipodystrophic *Pparg^fl/fl^ Adipoq-Cre* mice (14) to explore the role of adipose tissue in intestinal inflammation under steady-state conditions, following DSS-induced colitis, and through allogeneic fat transplantation models. Mass cytometry and single-cell RNA-seq (scRNA-seq) revealed an AGLCD-specific oligoclonal expansion of T cells associated with a somatic Neuroblastoma RAS viral oncogene homolog (*NRAS*) G13D mutation, which has previously been linked to the development of so-called RAS-associated autoimmune lymphoproliferative disorder (RALD) (15–18), suggesting that somatic mosaicism might contribute to T cell expansion in AGL. By using mouse models of lipodystrophy, we observed that adipose tissue loss did not primarily cause T cell lymphoproliferation; instead, lipodystrophic mice were protected from DSS-induced colitis due to impaired Th1 and Th17 cell function, which could be partially reversed by allogeneic transplantation of WT fat but not of leptin-deficient adipose tissue. Accordingly, treatment of the patient with AGLCD with recombinant leptin increased the abundance of Th17 cells in vivo, underscoring leptin’s role as a key regulator of proinflammatory T cells. Overall, we present what we believe to be first evidence that AGLCD might share features of a clonal autoimmune lymphoproliferative disease characterized by clonal expansion and a T cell–intrinsic de novo *NRAS* G13D mutation. Furthermore, our findings indicate that reducing adipose tissue mass may be beneficial for modulating intestinal inflammation in IBD by suppressing adipokine-induced proinflammatory T cells.

## Results

### Detection of high abundances of effector and central memory CD4^+^ and CD8^+^ T cells in the inflamed intestine and periphery in a patient with AGLCD.

**Figure 1:**
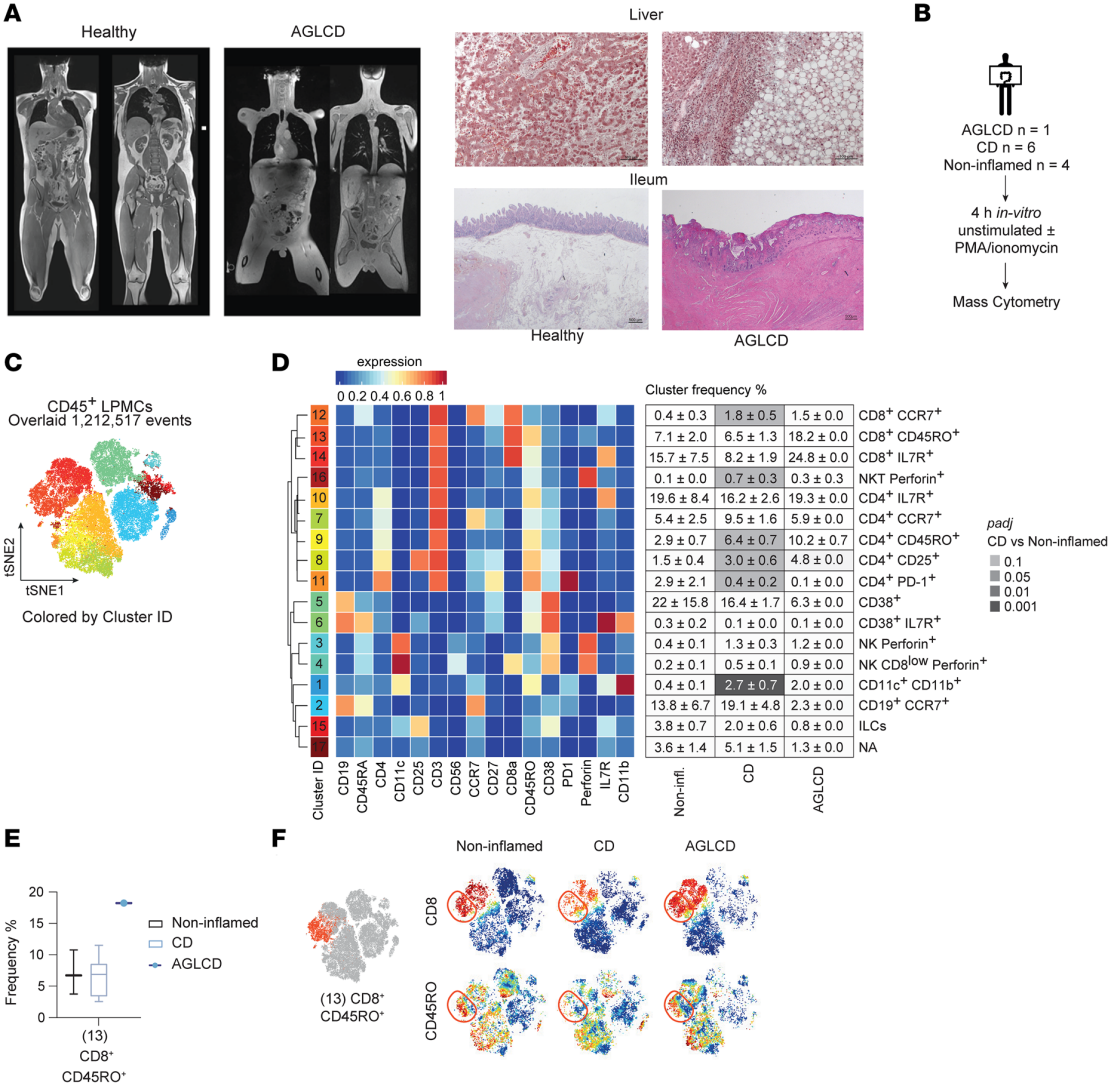
Enrichment of T cells in the small intestinal lamina propria in a patient with AGLCD as assessed by mass cytometry. (**A**) T1-weighted whole-body MRI (left) and liver (upper right) and ileal (lower right) histology from a patient with AGLCD versus a healthy control showing a complete absence of subcutaneous and visceral fat, hepatic steatosis with inflammation, and severe ileal lesions. (**B**) Workflow for mass cytometry. LPMCs were isolated from terminal ileum surgical resections from patients with CD (*n* = 6), 1 patient with AGLCD, and noninflamed controls (*n* = 4). Cells were stimulated with or without PMA and ionomycin for 4 hours. (**C**) FlowSOM clustering of merged flow cytometry standard files identified 17 cell clusters among CD45^+^ LPMCs. tSNE, *t*-distributed stochastic neighbor embedding. (**D**) Heatmap showing relative expression of 16 phenotypic markers across clusters. The accompanying table summarizes the mean cluster frequencies (± SEM) in noninflamed (Non-infl.), CD, and AGLCD samples. Statistical comparisons were performed using the edgeR framework with a negative binomial generalized linear model and FDR correction (10%, Benjamini-Hochberg). ILCs, innate lymphoid cells; padj., adjusted *P* value. (**E**) Frequency of CD8^+^CD45RO^+^ effector T cells (cluster 13) across groups. Boxes indicate IQRs, whiskers denote minimum to maximum values, and lines represent the median. (**F**) viSNE plots of representative samples (noninflamed, CD, and AGLCD) colored by marker expression (blue = low; red = high). Data in **C**–**F** are from 1 mass cytometry experiment.

The pathogenesis of AGL remains poorly understood, and the drivers of autoimmunity in AGL have yet to be elucidated. To gain insights into the mechanisms of chronic intestinal inflammation in AGL, we conducted an in-depth analysis of the intestinal immune cell composition of a rare patient with AGLCD (11), who presented with a complete absence of adipose tissue, leptin deficiency, severe metabolic dysfunction, progressive liver disease requiring liver transplantation at 15 years of age, and CD with recurrent ileocolonic inflammation from age 11 that ultimately required long-term immunosuppression with tacrolimus, mycophenolate, and TNF-α blockade (Figure 1A).

Thus, we performed mass cytometry to compare the ileal immune cell compartment in the patient with AGLCD with fat-proficient noninflamed control individuals or patients with CD (Figure 1B and Supplemental Figure 1; supplemental material available online with this article; https://doi.org/10.1172/JCI192322DS1), resolving a total of 17 intestinal immune cell clusters (Figure 1, C and D). Among these, CD4^+^IL-7R^–^CD25^+^ Tregs (cluster 8) as well as CD4^+^CD45RO^+^ effector T cells (cluster 9), which have been identified as a cell signature indicative of IBD (19), were enriched in both the AGLCD and CD patients when compared with noninflamed controls (Figure 1D). Interestingly, only the patient with AGLCD showed an expansion of CD8^+^ CD45RO^+^ effector T cells (cluster 13), which was not detected in the patients with CD or the noninflamed controls (Figure 1, E and F). Supplemental Figure 2 summarizes the differences in activation markers, cytokines, and cytolytic proteins between cell clusters of the AGLCD patient, CD patients, and noninflamed controls, highlighting an upregulation of IL-17 in CD8^+^CD45RO^+^ T cells and IL-10 in CD4^+^ T cells from the patient with AGLCD.

**Figure 2:**
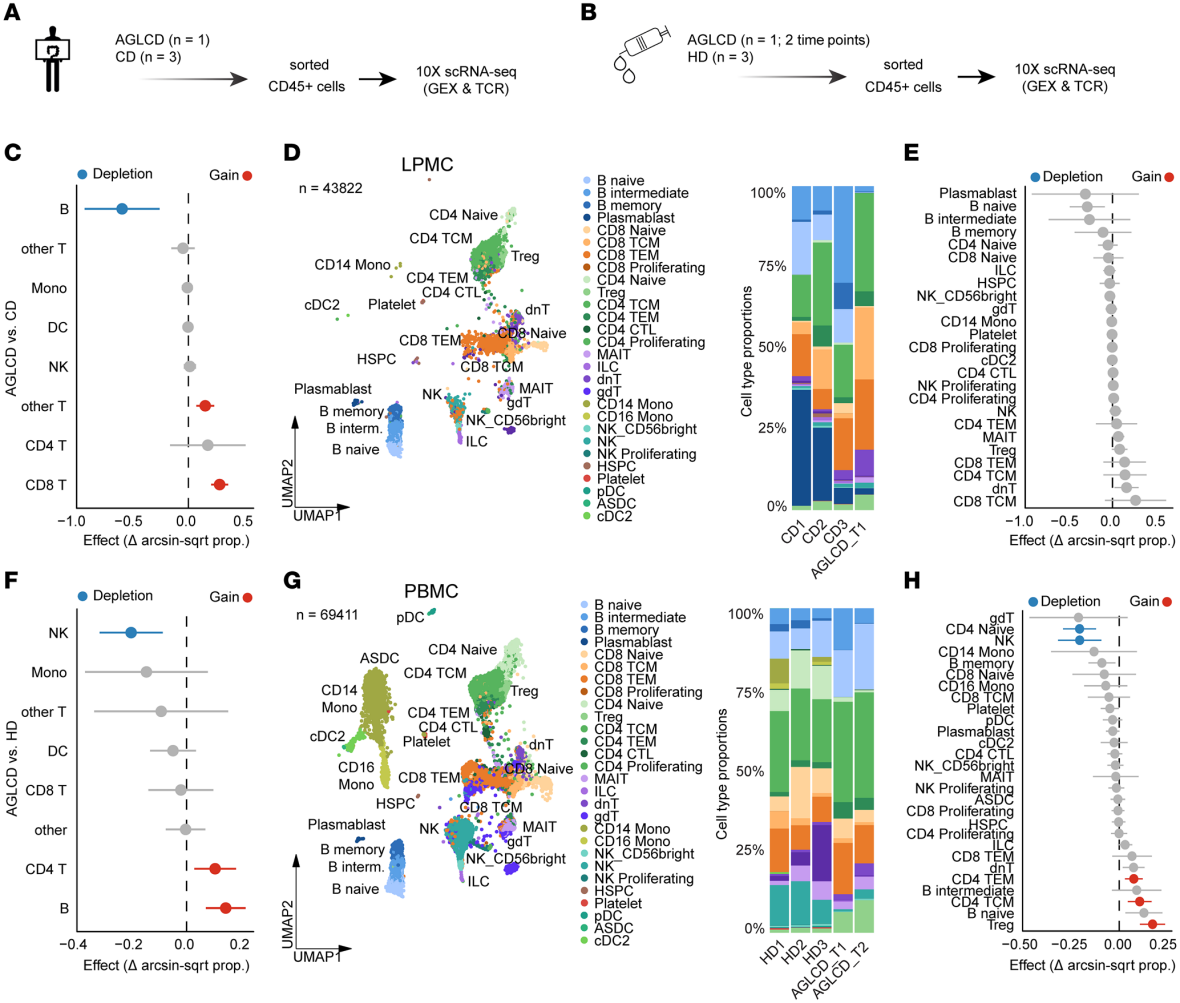
Altered peripheral and intestinal immune cell compositions in AGLCD revealed by single-cell profiling. (**A** and **B**) Workflow for scRNA-seq of CD45^+^ immune cells from small intestine or peripheral blood of the patient with AGLCD and from 3 lean male HDs and 3 patients with CD. GEX, gene expression; TCR, single T cell receptor sequencing. (**C**–**E**) Intestinal immune cell (LPMC) comparisons showing the AGLCD sample alongside CD controls. (**F**–**H**) PBMC comparisons showing the AGLCD sample alongside HDs. (**D** and **G**) Uniform manifold approximation and projections (UMAPs) and bar graphs display cell-type frequencies, and forest plots show arcsine square root–transformed (Δ arcsin-sqrt prop.) relative abundance of lymphocyte clusters for the CD (**C** and **E**) and HD (**F** and **H**) groups against the AGLCD sample. ASDC, AXL+ Siglec-6+ dendritic cell; cDC2, conventional Dendritic Cell 2; CTL, CD4+ cytotoxic T; dnT, double-negative T; gdT, gamma-delta T; HSPC, hematopoietic stem and progenitor cell; interm., intermediate; Mono, monocytes; pDC, plasmacytoid DCs; TCM, central memory T cells; TEM, effector memory T cells.

To better characterize the intestinal immune cell composition of the patient with AGLCD, we performed scRNA-seq on FACS-sorted ileal CD45^+^ lamina propria mononuclear cells (LPMCs) from the patient with AGLCD and 3 control patients with CD (Figure 2A), as well as on CD45^+^ PBMCs collected from the patient with AGLCD at 2 independent time points 2 years apart, alongside samples from 3 age-matched, lean male healthy donors (HDs) (Figure 2B). As shown in Figure 2, C–F, the patient with AGLCD exhibited an expansion of intestinal CD4^+^ and CD8^+^ T cells, accompanied by a reduced abundance of total B cells (Figure 2C). In particular, central memory and effector memory CD4^+^ and CD8^+^ T cells were increased, whereas naive B cells and plasmablasts were decreased in the patient with AGLCD compared with the CD controls (Figure 2, D and E).

Consistent with these findings, we previously used mass and flow cytometry to identify a systemic expansion of CD8^+^ T cells in the peripheral blood of the patient with AGLCD (11), which demonstrated that the patient with AGLCD had a systemic occurrence of expanded CD8^+^ T cells. Likewise, scRNA-seq analyses of the CD45^+^ PBMCs revealed a higher abundance of peripheral B cells and CD4^+^ T cells, together with a reduced frequency of NK cells in the patient with AGLCD when compared with HDs (Figure 2F). More specifically, the patient with AGLCD showed increased CD4^+^ and CD8^+^ effector memory T cell subsets, more Tregs, and a reduced frequency of NK cells and naive CD4^+^ T cells relative to HDs (Figure 2, G and H). These findings confirm our previous observations of defective NK cell homeostasis (11) and highlight a persistent expansion of T cells in the patient with AGLCD, in both the peripheral blood and the gut at the site of inflammation.

Considering that these immune alterations occur in the complete absence of adipose tissue, we next asked whether adipose loss itself might contribute to or protect against immune dysregulation. Adipose tissue plays a critical role in regulating the immune system in the context of IBD as well as rheumatoid arthritis (7, 20). Given that patients with AGL frequently experience autoimmune-related comorbidities such as arthritis, glomerulonephritis, and autoimmune hepatitis (2), we sought to determine whether the observed accumulation of T cells was a consequence of the absence of adipose tissue. Accordingly, we examined the effect of adipose tissue loss on immune cell composition, focusing on T cell differentiation, function, and expansion in lipodystrophic *Pparg^fl/fl^ Adipoq-Cre* mice.

### Lipodystrophic mice show alterations in splenic NK and intestinal T cell composition and have increased intestinal antimicrobial peptide expression.

**Figure 3:**
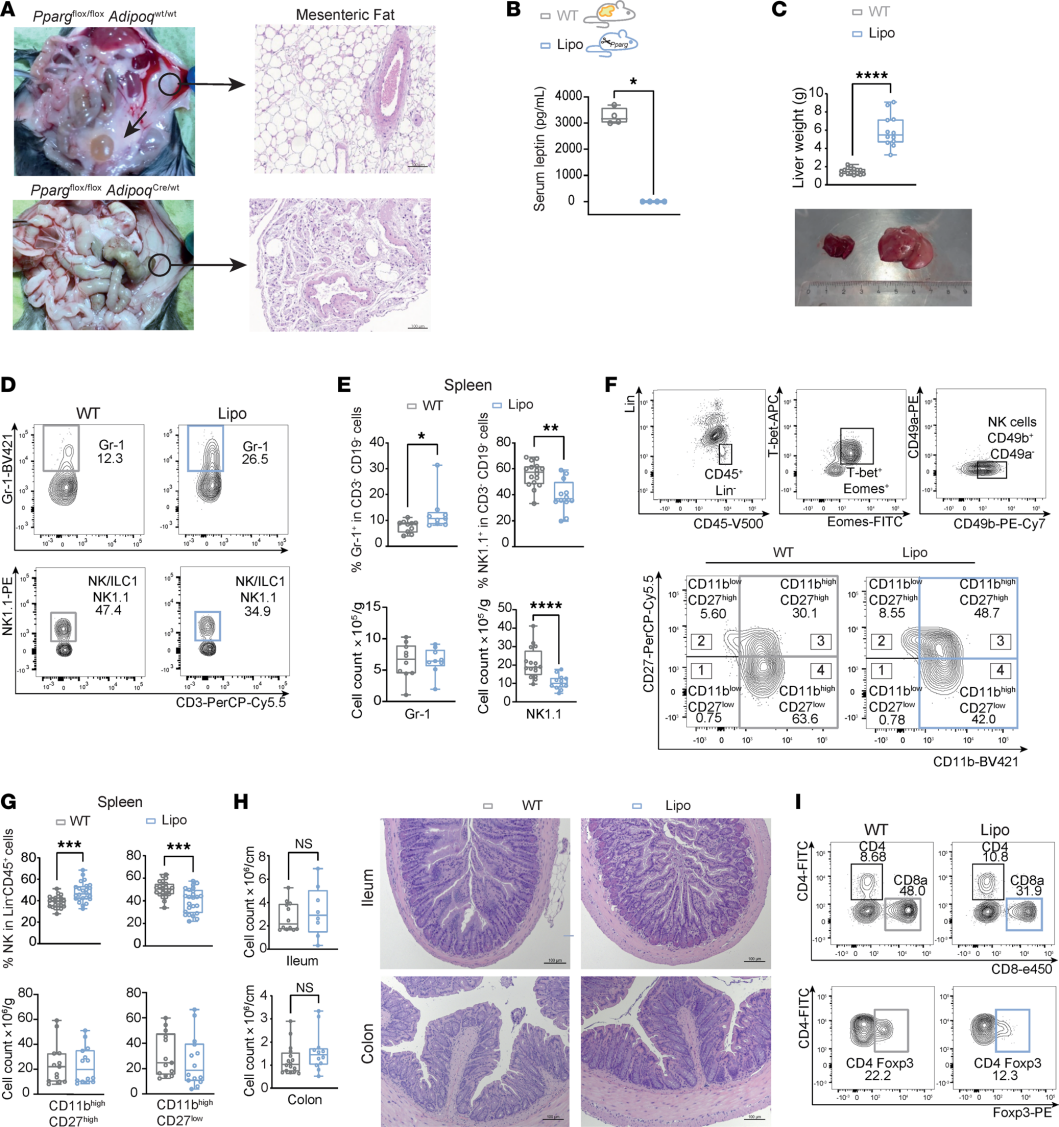
Lipodystrophic *Pparg^fl/fl^ Adipoq-Cre* mice mimic the hepatic phenotype of the patient with AGLCD and show defects in the development of NK and T cells under steady-state conditions. (**A**) Representative images of *Pparg^fl/fl^ Adipoq-Cre* (Lipo) and WT mice showing the complete absence of fat tissue and H&E-stained images of mesenteric adipose tissue (arrows indicate mesenteric and gonadal fat tissue). Scale bars: 100 μm. (**B**) Leptin plasma levels (WT *n* = 4; Lipo *n* = 4). (**C**) Liver weights of WT mice (*n* = 16) and *Pparg^fl/fl^ Adipoq-Cre* mice (*n* = 12) from 2 independent experiments with a representative image. (**D**–**G**) Flow cytometric assessment of splenic NK cells from *Pparg^fl/fl^ Adipoq-Cre* mice (*n* = 9–24) compared with WT littermate cells (*n* = 10–23). Data were pooled from 3 independent experiments. (**H**) Representative H&E-stained images and immune cell counts (millions/cm) in the terminal ileum or colon as assessed by histology (WT: *n* = 12–16; Lipo: *n* = 8–12). Scale bars: 100 μm. (**I**) Gating strategy of T cells and CD4^+^FoxP3^+^ Tregs. The line in the box indicates the median. Boxes range from the 25th to 75th percentiles. Whisker plots show the minimum (smallest) and maximum (largest) values while the line in the box indicates the median. *P < 0.05, **P < 0.01, ***P < 0.001, and ****P < 0.0001, by unpaired 2-tailed *t* test with Welch’s correction.

As previously published by Wang et al. (14), lipodystrophic *Pparg^fl/fl^ Adipoq-Cre* mice exhibited a complete absence of visceral fat (Figure 3A), consecutive absence of adipokines including leptin in the serum (Figure 3B) and developed nonalcoholic steatohepatitis analogous to the patient with AGLCD (Figure 3C and Supplemental Figure 3A). However, no accumulation of lipids was observed in other tissues, including the pancreas, spleen, kidneys, or cardiac muscle (Supplemental Figure 3A). Additionally, splenomegaly with an enlarged red pulp and larger-sized glomeruli in the kidneys were detected, as previously described (21) (Supplemental Figure 3A). Although it has been reported that knockout models of fat-derived signals, such as leptin (*ob/ob*) or leptin receptor–deficient (*db/db*) mice exhibit disturbances in immune cell homeostasis compared with WT controls (22), scarce knowledge exists regarding the effect of lipodystrophy on immune cell homeostasis, and more specifically on T cell homeostasis. To gain insight into the effect of adipose tissue loss on immune cell composition in lipodystrophic mice at steady state, we conducted a comprehensive immune cell characterization of the spleen and intestinal tissue using flow cytometry (gating strategies are summarized in Supplemental Figure 3, B and C). Specifically, splenocytes and LPMCs isolated from the ileum and colon were stained with antibody cocktails targeting CD4 and CD8 for the identification of T cells, CD19 for B cells, and Gr-1 for myeloid subsets. NK1.1, CD49b, CD49a, T-bet, Eomes, CD11b, and CD27 were used to characterize NK subsets according to the 4-stage model of NK cell maturation CD11b^lo^CD27^lo^ → CD11b^lo^CD27^hi^ → CD11b^hi^CD27^hi^ → CD11b^hi^CD27^lo^ (23). Moreover, to identify discrepancies in cytokine production, cells were stimulated in vitro with PMA/ionomycin for 4 hours and then stained intracellularly for TNF-α, IFN-γ, and IL-17A (Supplemental Figure 3, B and C). Notably, lipodystrophic mice showed a reduction in frequencies and absolute cell numbers of splenic NK1.1^+^ NK and type 1 innate lymphoid cells and an increased abundance of Gr-1^+^ myeloid subsets compared with WT littermate controls (Figure 3, D and E). Further analysis of the CD49b^+^ NK cell population revealed a higher frequency of less mature CD11b^hi^CD27^hi^ NK cells and a reduced percentage of the highly cytolytic CD11b^hi^CD27^lo^ NK cell subset in lipodystrophic mice compared with WT mice (Figure 3, F and G). Although no obvious histological abnormalities were detected in the small or large intestine of lipodystrophic versus WT animals (Figure 3H), flow cytometric analysis revealed a significantly reduced frequency of CD8^+^ T cells in the colon of fat-deficient lipodystrophic mice compared with WT controls (Figure 3I), while the overall numbers of intestinal and splenic T cell subsets did not significantly differ between the 2 groups (Supplemental Figure 3, D–F).

These data collectively indicate that adipose tissue and its secreted factors are required to maintain immune cell homeostasis, including the differentiation of splenic NK cells and, to a lesser extent, intestinal CD8^+^ T cells under steady-state conditions. Additionally, our findings show that the absence of adipose tissue and missing fat-derived signals in AGL were not responsible for the expansion of peripheral and intestinal CD4^+^ and CD8^+^ T cells observed in the patient with AGLCD.

**Figure 4:**
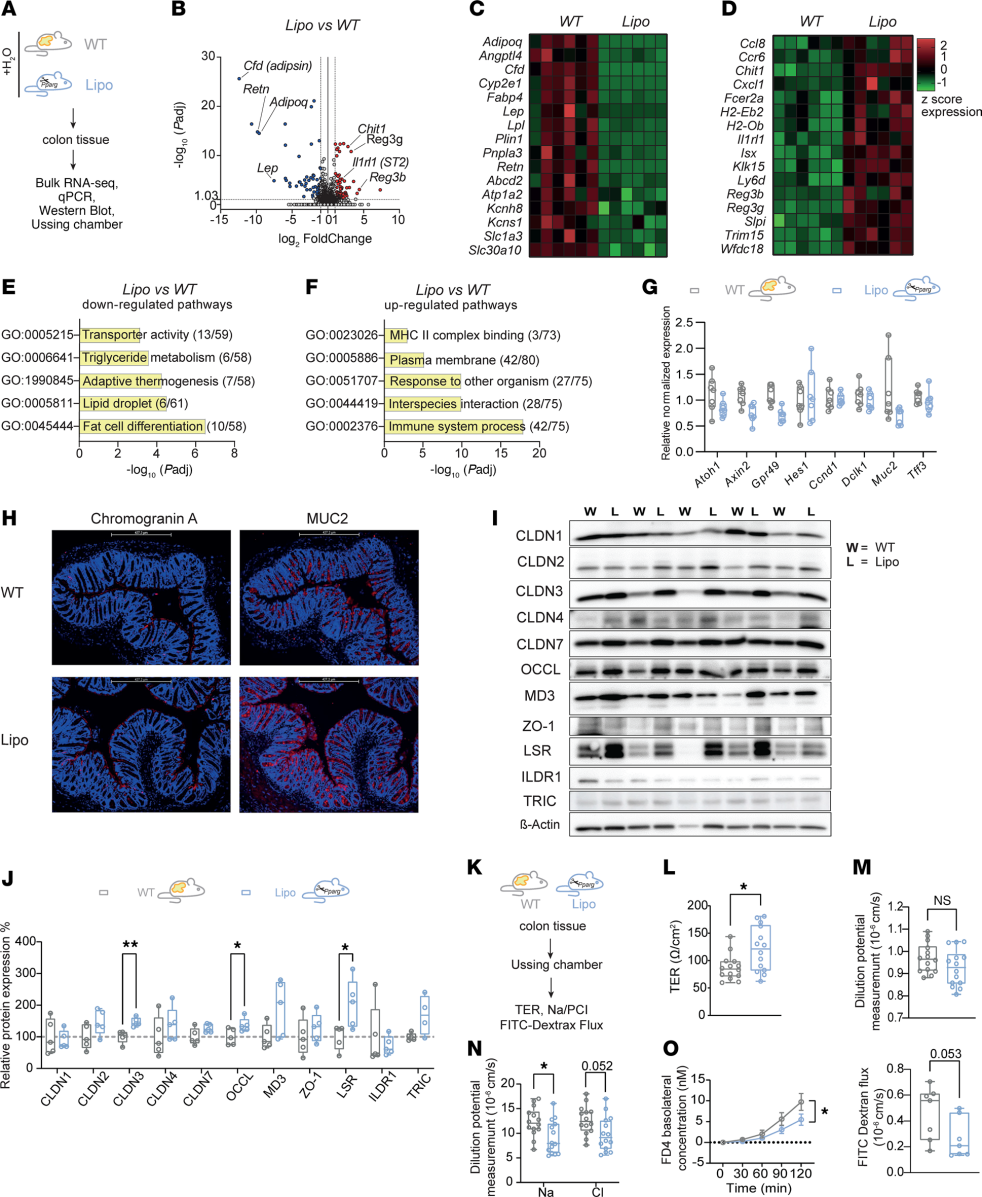
Lipodystrophic mice display normal mucus production and have increased expression of epithelial TJ proteins. (**A**) Experimental setup of bulk RNA-seq experiments of colon tissue obtained from WT and lipodystrophic mice. (**B**) Volcano plot showing differentially regulated genes between *Pparg^fl/fl^ Adipoq-Cre* and WT mice (*n* = 6 per group). (**C** and **D**) Heatmaps showing differentially regulated genes related to adipose tissue –secreted factors and antimicrobial factors between *Pparg^fl/fl^ Adipoq-Cre* mice and WT littermates (*n* = 6 per group). (**E** and **F**) Bar graphs displaying pathway analyses of significantly regulated genes in lipodystrophic mice. (**G**) Box-and-whisker plots showing qPCR expression of epithelial differentiation markers in colonic tissue obtained from 6 WT mice and 6 *Pparg^fl/fl^ Adipoq-Cre* mice. (**H**) Representative immunohistochemical staining of colonic tissue obtained from WT controls and *Pparg^fl/fl^ Adipoq-Cre* mice showing epithelial MUC2 and chromogranin A expression. Scale bars: 427.2 μm. (**I** and **J**) Western blot analyses of colonic TJ proteins in WT mice (*n* = 5) and *Pparg^fl/fl^ Adipoq-Cre* mice (*n* = 4–5). (**K**) Intestinal epithelial barrier function was evaluated ex vivo by mounting murine colonic tissue in Ussing chambers. (**L**) TER, (**M**) permeability ratios of sodium to chloride (PNa/PCl), and (**N**) the respective absolute permeabilities for sodium and chloride. Each point represents an individual measurement (*n* = 7 per group with 2 measurements for each). (**O**) Basolateral increase in FD4 concentration over time. FD4 permeability is shown for WT and *Pparg^fl/fl^ Adipoq-Cre* mice (*n* = 7 per group). Statistical differences were calculated using the Mann-Whitney *U* test. Boxes range from the 25th to 75th percentiles. Whisker plots show the minimum (smallest) and maximum (largest) values (**G**, **J**, **L**–**N**, and **O** right). Data indicate the mean ± SEM (**O** left). *P < 0.05 and **P < 0.01, by an unpaired 2-tailed *t* test with Welch correction for panel **J**, and by a Mann-Whitney U 2-tailed test for panels **L**, **M**, and **O**. GO, Gene Ontology.

To gain further insight into the effect of adipose tissue absence on mucosal homeostasis, we conducted bulk RNA-seq analyses of colonic tissue from 6 lipodystrophic and 6 control mice (Figure 4A). We detected 73 downregulated and 83 upregulated genes in the colonic tissue of lipodystrophic mice compared with WT littermates. Consistent with the complete absence of adipose tissue, the most significantly downregulated genes in lipodystrophic mice were those transcribing for adipose-derived factors including adipsin (*Cfd*), resistin (*Retn*), leptin (*Lep*), and adiponectin (*Adipoq*) (Figure 4, B and C). In addition, colonic tissue from lipodystrophic mice showed significantly higher mRNA transcripts of antimicrobial peptides (AMPs), including *Reg3b*, *Reg3g*, and *Chitin1*, compared with WT controls (Figure 4D). Pathway analyses revealed that lipodystrophic mice exhibited a differential regulation of genes primarily involved in adipocyte differentiation, regulation of thermogenesis, and pathways controlling lipid droplet formation (Figure 4, E and F). Notably, we did not detect major alterations in intestinal epithelial cell differentiation between lipodystrophic and WT mice by RNA-seq or quantitative PCR (qPCR) (Figure 4, E–G). Similarly, immunohistochemical staining of colonic tissue revealed comparable levels of chromogranin A and mucin 2 (MUC2), indicating that lipodystrophy does not affect the homeostasis of intestinal neuroendocrine or goblet cells (Figure 4H).

Several publications have proposed that excessive production of adipose-derived factors, such as leptin, could impair epithelial barrier function in patients with obesity by affecting the expression of tight junction (TJ) proteins, including claudins, in intestinal and mammary gland epithelial cells (24–26), which are crucial for epithelial barrier function and repair (27, 28). We therefore examined the effect of fat loss on TJ protein expression levels in intestinal epithelial cells by Western blot analyses. Specifically, we analyzed members of the claudin (Cldn) family, which are expressed in the colon and mainly determine the paracellular barrier properties for ions and small solutes (29). We furthermore studied TJ-associated MARVEL proteins (occludin, tricellulin, marvelD3), of which occludin and tricellulin are pivotal for paracellular macromolecule permeability, as well as the intestinal angulins angulin 1 (also known as LSR) and angulin 2 (also known as ILDR1), which control the correct localization of tricellulin and the regulation of the permeability of the tricellular TJ (26, 30). As an important TJ scaffolding protein, we additionally analyzed the expression of zonula occludens 1 (ZO-1) (29, 31). As shown in Figure 4, I and J, we observed significantly elevated expression of claudin 3 (CLDN3), occludin (OCCL), and the lipolysis-stimulated lipoprotein receptor (LSR) in colonic tissue of lipodystrophic mice when compared with expression levels in WT controls. This suggests that lipodystrophic mice may exhibit an augmented epithelial resistance to the translocation of luminal pathogens due to their enhanced expression of TJ proteins and antimicrobial peptides. To functionally test epithelial barrier functions of lipodystrophic mice, we therefore compared the properties of colon explants of lipodystrophic mice and WT littermates in Ussing chamber experiments (Figure 4K), which revealed higher transepithelial electric resistance (TER) (Figure 4L). However, the measurement for ion permeability was not affected by ion charge selectivity (Figure 4M), presumably because of the decreased permeability for sodium (Figure 4N). In addition, flux rates of the macromolecular 4 kDa FITC-dextran (FD4) were decreased in lipodystrophic mice compared with WT controls (Figure 4O), highlighting the idea that epithelial barrier functions improve in lipodystrophic mice in the absence of fat-derived signals.

### Lipodystrophic mice are protected from chronic DSS-induced colitis.

**Figure 5:**
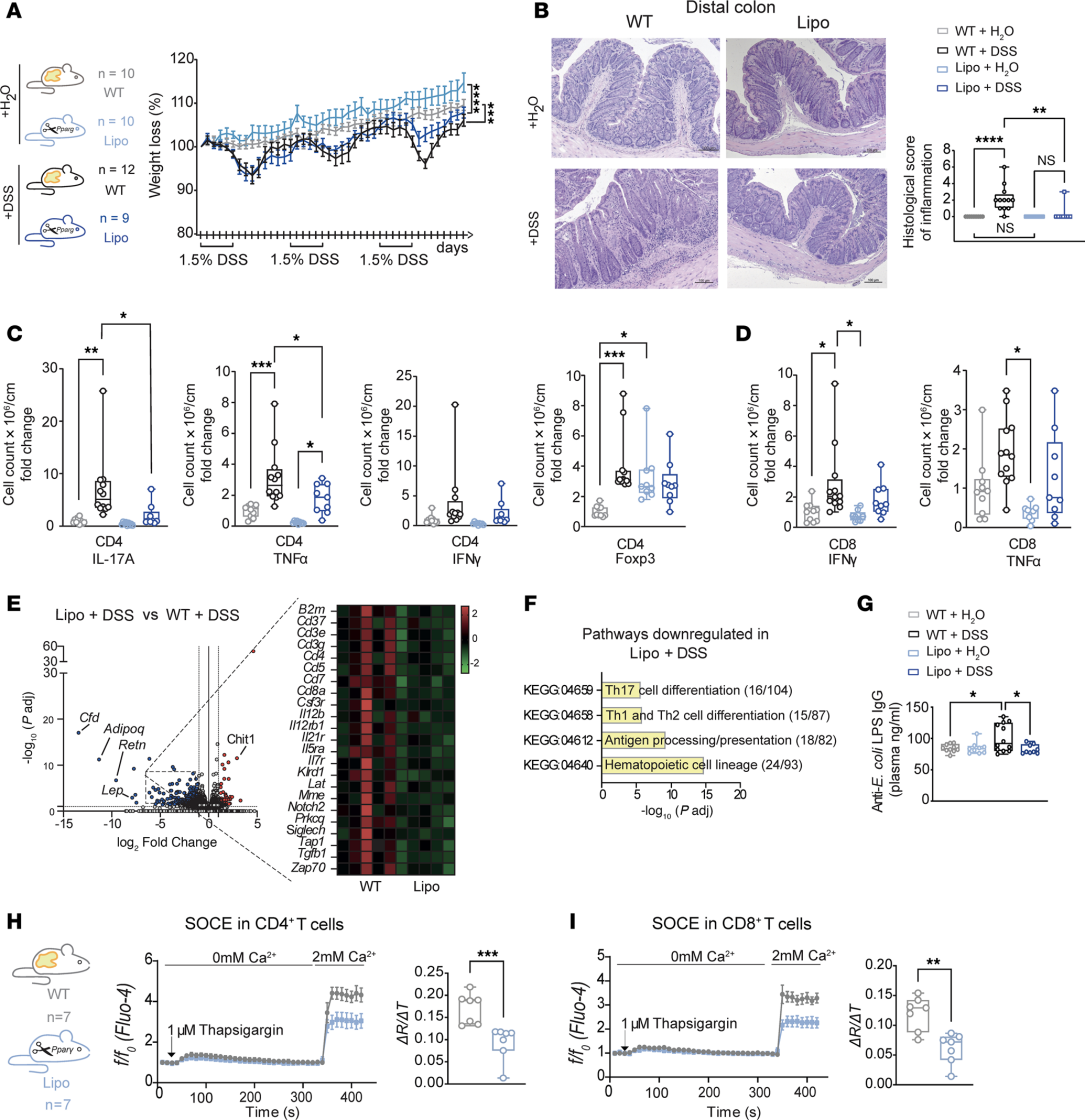
Lipodystrophic mice are protected from DSS-induced colitis. (**A**) Experimental groups for chronic DSS colitis induction and percentage of weight loss. Differences were calculated by 1-way ANOVA with Šídák’s correction. Lines indicate mean values. (**B**) Histologic colitis score with representative H&E staining of distal colon sections (WT + H_2_O: *n* = 10; WT + DSS: *n* = 12; Lipo + H_2_O: *n* = 10; Lipo + DSS: *n* = 9). Differences were tested by Kruskal-Wallis with Dunn’s correction; data were pooled from 2 independent experiments. Relative cell numbers (per cm) in colonic tissue samples from WT + H_2_O (*n* = 10), WT + DSS (*n* = 12), and Lipo + H_2_O (*n* = 9) mice and for CD4^+^ (**C**) and CD8^+^ T cells (**D**), as assessed by flow cytometry. Differences were calculated by Mann-Whitney *U* test; data were pooled from 2 experiments. (**E**) Volcano plot of differentially regulated genes between *Pparg^fl/fl^ Adipoq-Cre* and WT mice (*n* = 5/group) and heatmap of downregulated inflammation-related genes in DSS-treated *Pparg^fl/fl^ Adipoq-Cre* versus DSS-treated WT controls. (**F**) Kyoto Encyclopedia of Genes and Genomes (KEGG) pathway analysis and representative genes significantly downregulated (*P*adj < 0.05, log_2_ fold change <–1) in Lipo + DSS (*n* = 5) versus WT + DSS (*n* = 5) mice. (**G**) Plasma anti–*E. coli* LPS IgG levels (WT + H_2_O: *n* = 10; WT + DSS: *n* = 12; Lipo + H_2_O: *n* = 9; Lipo + DSS: *n* = 9). Significance was determined by 1-way ANOVA with Šídák’s correction. (**H** and **I**) Calcium influx upon thapsigargin treatment in splenic CD4^+^ (**H**) and CD8^+^ (**I**) T cells from *Pparg^fl/fl^ Adipoq-Cre* mice and WT littermates (*n* = 7/group), as assessed by flow cytometry. Data were pooled from 2 experiments. Boxes represent 25th–75th percentiles; whiskers represent minimum and maximum; line indicates the median. SEM is shown where applicable. f/f_0_, MFI of Fluo-4 (*f*) normalized to MFI average detected during 30 s baseline measurement (f0); ΔR/ΔT, calcium influx rate. Boxes range from 25th–75th percentiles. Whisker plots show minimum (smallest) and maximum (largest) values; line in box indicates the median. Data indicate the mean ± SEM (**A**, **H**, and **I**). *P < 0.05, **P < 0.01, ***P < 0.001, and ****P < 0.0001, by 1-way ANOVA corrected for multiple comparisons with Šídák’s test (**A**–**D** and **G**) and Mann-Whitney *U* 2-tailed test (**H** and **I**).

To rule out the possibility that the T lymphoproliferation observed in the patient with AGLCD only arises during chronic intestinal inflammation in AGL, we next challenged lipodystrophic or WT littermates with 3 cycles of 1.5% DSS, which induced weight loss in both WT and lipodystrophic mice (Figure 5A). We found that only fat-proficient WT mice developed severe chronic colitis with higher scores in stool consistency (Supplemental Figure 4A), whereas lipodystrophic animals were characterized by significantly reduced intestinal inflammation as assessed by histology (Figure 5B).

Flow cytometric analyses of colonic immune cells revealed that DSS treatment effectively induced colitis in WT mice, which harbored significantly elevated numbers of CD4^+^ Tregs and proinflammatory CD4^+^ and CD8^+^ T cell subsets compared with WT littermates receiving only water (Figure 5C). Notably, lipodystrophic mice treated with DSS were characterized by a significant reduction in colonic CD4^+^ T cells, that produced TNF-α or IL-17A, and a decline in Tregs (Figure 5D) in comparison with WT mice treated with DSS. Additionally, there was a notable decrease in intestinal CD8^+^ T cells in lipodystrophic mice, which further supports the notion that fat-derived signals play a pivotal role in the differentiation and function of proinflammatory T cells during intestinal inflammation. Consistently, bulk RNA-seq of colonic tissue substantiated that DSS-treated lipodystrophic mice had significantly decreased expression of gene sets linked to T cell differentiation and antigen presentation when compared with their DSS-treated WT littermates (Figure 5, E and F). In addition, further gene set enrichment analysis (GSEA) not only revealed a significant decline in the expression of genes associated with inflammation but also showed a reduced epithelial-mesenchymal transition signature in DSS-treated lipodystrophic mice (Supplemental Figure 4B), indicating a preserved intestinal epithelial polarity and adherence in the absence of fat. Notably, we also observed that DSS-treated lipodystrophic mice had significantly less bacterial translocation through the intestinal barrier, as fat-deficient mice harbored lower serum levels of anti–*E*. *coli* LPS IgG compared with WT mice receiving DSS (Figure 5G).

To better understand why T cells from lipodystrophic mice exhibited reduced cytokine production in the absence of adipose tissue, we conducted a comparative analysis of the ability of CD4^+^ and CD8^+^ T cells to flux calcium through store-operated calcium entry (SOCE) between T cells obtained from lipodystrophic mice or WT littermates. SOCE, mediated by ORAI and STIM molecules, is a key signaling pathway controlling proinflammatory T cell function in murine and human IBD downstream of T cell receptor (TCR) activation (19). As shown in Figure 5, H and I, SOCE was significantly reduced in splenic CD4^+^ and CD8^+^ T cells derived from lipodystrophic mice when compared with T cells from WT mice. This suggests that T cells from lipodystrophic mice had a defect in the homeostasis of SOCE, which may have contributed to impaired T cell function, ultimately resulting in decreased production of proinflammatory cytokines and a reduction in intestinal inflammation, as SOCE signaling is required for the production of IFN-γ, IL-17A, and TNF-α in both CD4^+^ and CD8^+^ T cells and genetic or pharmacologic ablation of SOCE results in reduced differentiation of Th1 cells, Th17 cells, and Tregs (19). Elevated lipid concentrations and intracellular lipid droplet accumulation have been shown to act as cytoplasmic Ca²^+^ sinks, thereby impairing SOCE in immune cells (32). To determine whether the observed SOCE defect was T cell intrinsic or secondary to lipodystrophy, we incubated WT splenocytes with plasma from lipodystrophic or WT mice. Lipodystrophic plasma contained elevated triglycerides, induced lipid droplet formation in CD4^+^ and CD8^+^ T cells, and significantly reduced SOCE, indicating that triglyceride-driven lipid droplet accumulation can act as intracellular Ca²^+^ sinks and thereby secondarily diminish SOCE in T cells (Supplemental Figure 5, A–F).

### Transplantation of allogeneic fat tissue partially rescues intestinal inflammation in lipodystrophic mice via a leptin-dependent induction of proinflammatory T cells.

**Figure 6:**
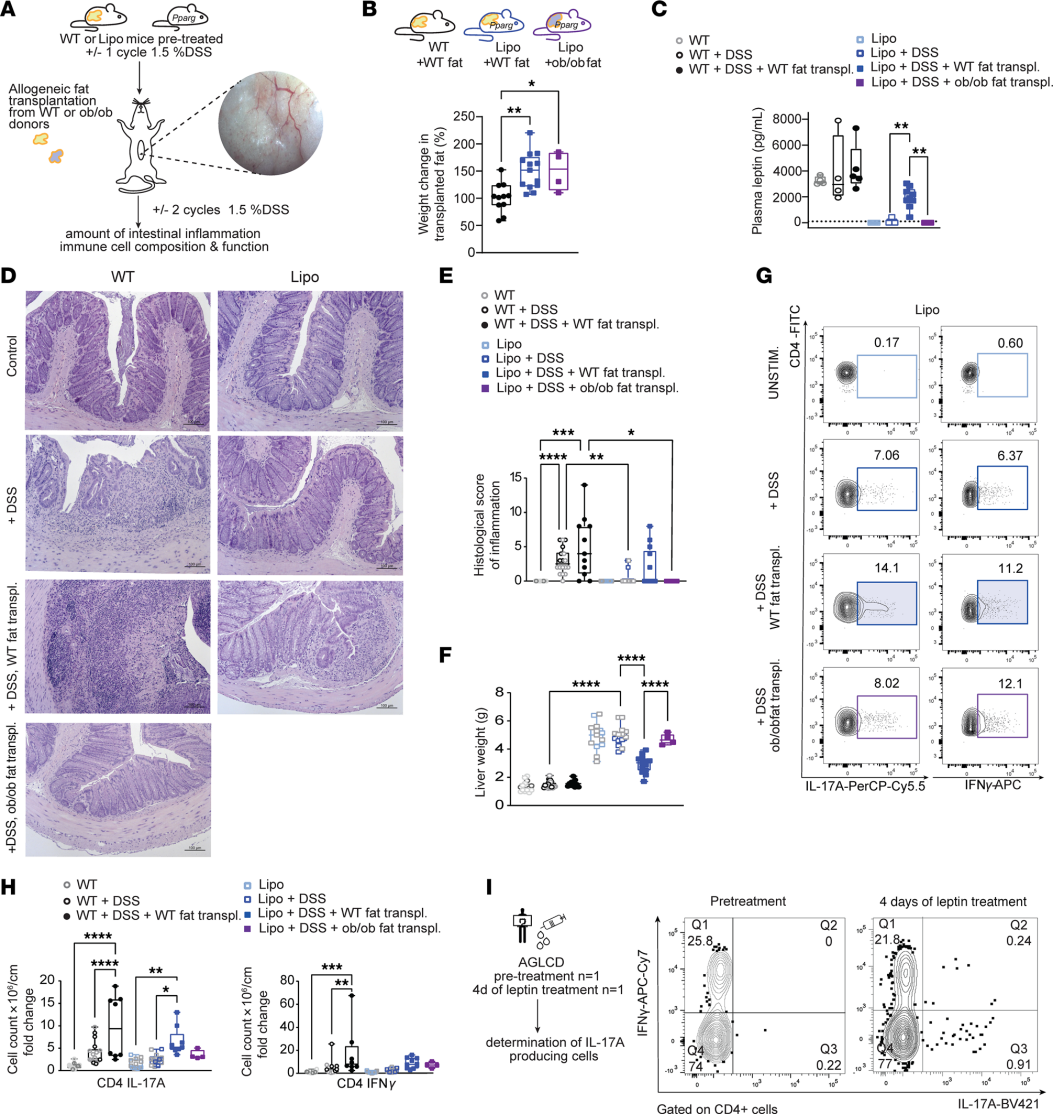
Allogeneic fat transplantation partially restores basal leptin levels, reverses steatohepatitis, and promotes intestinal inflammation via induction of intestinal proinflammatory T cells. (**A**) Experimental design: After 1 DSS cycle (1.5%), lipodystrophic or WT mice were transplanted with 500–600 mg adipose tissue from WT or leptin-deficient *ob/ob* donors by mini-laparotomy before undergoing another 2 cycles of DSS. Image on right shows vascularized transplanted fat 1 month after surgery. (**B**) Weight change of transplanted fat tissue relative to baseline. (**C**) Plasma leptin levels (*n* = 4–9). (**D**) Representative images of H&E- stained colon sections from transplanted versus nontransplanted DSS-treated animals. Scale bars: 100 μm. (**E**) Box-and-whisker plots summarizing the histologic inflammation score of fat-transplanted and nontransplanted animals. Data shown were pooled from 2 independent transplantation experiments (bold symbols) and additional control data points derived from nontransplantation DSS experiments (light gray) shown in Figure 3 (*n* = 4–18). (**F**) Liver weights of transplanted WT and *Pparg^fl/fl^ Adipoq-Cre* mice (*n* = 4–18; data were pooled from 5 experiments). (**G**) Representative FACS plots showing IFN-γ and IL-17A production in colonic CD4^+^ T cells. UNSTIM., unstimulated. (**H**) Box-and-whisker plots summarizing absolute numbers of IFN-γ– and IL-17A–producing CD4^+^ T cells normalized to WT mice (*n* = 4–18; data were pooled from 5 experiments). Statistical differences were calculated by 1-way ANOVA with Šídák’s correction. Each point represents 1 mouse; boxes range from the 25th-75th percentiles. Whisker plots show the minimum (smallest) and maximum (largest) values while the line in the box indicates the median. (**I**) Experimental setup and representative FACS plots showing IFN-γ– and IL-17A–producing CD4^+^ T cells in peripheral blood of a patient with AGLCD before and 4 days after daily recombinant leptin substitution. transpl., transplantation. Data indicate the mean ± SEM. *P < 0.05, **P < 0.01, ***P < 0.001, and ****P < 0.0001, by 1-way ANOVA with Tukey’s multiple comparisons test for **B**, **C**, **E**, **F**, and **H**.

To further support our observation that adipose tissue is required for proper T cell homeostasis, we next examined how the reintroduction of adipose tissue would affect the T cell compartment of lipodystrophic mice under steady-state and inflammatory conditions. Using allogeneic adipose tissue transplantation models, we transplanted DSS-treated mice with either adipose tissue from WT donor mice or with adipose tissue from leptin-deficient *ob/ob* donor mice (Figure 6A and Supplemental Figure 6A). We observed that transplanted adipose tissue showed good engraftment with spontaneous vascularization, led to fat uptake in transplanted adipose tissue, and partially restored basal leptin production in recipient lipodystrophic mice (Figure 6, A–C and Supplemental Figure 6B). As expected, leptin production could thus only be observed in recipient lipodystrophic mice receiving adipose tissue from WT donor mice but not in lipodystrophic mice transplanted with fat tissue from leptin-deficient *ob/ob* mice (Figure 6C). Remarkably, transplantation of WT fat partially rescued the hepatic phenotype of lipodystrophic mice by reducing lipid load and liver weight (Figure 6F, Supplemental Figure 6, C and D, and Supplemental Figure 7C), underscoring the engraftment of functional adipose tissue after fat transplantation. Furthermore, Western blot analyses of colonic tissue revealed that WT fat transplantation could partially reverse the overexpression of colonic TJ proteins observed in lipodystrophic mice under steady-state conditions (Supplemental Figure 6, E and F), indicating that epithelial barrier functions decreased with increasing fat mass.

As shown in Figure 6, D and E, transplantation of WT adipose tissue resulted in comparable levels of histologic inflammation and weight loss in WT and lipodystrophic mice treated with DSS (Supplemental Figure 7, A and B). However, lipodystrophic mice that did not undergo fat transplantation or receive fat from leptin-deficient *ob/ob* mice showed significantly reduced inflammation or no inflammation upon DSS treatment when compared with DSS-treated WT mice that had undergone transplantation with WT adipose tissue (Figure 6, D and E). Analysis of bulk RNA-seq data from colon tissue revealed that transplantation of WT fat tissue led to an enrichment of a proinflammatory Th1/Th2 gene signature in DSS-treated lipodystrophic mice when compared with DSS-treated lipodystrophic mice that did not receive adipose tissue (Supplemental Figure 7, E and F). Interestingly, we also observed that the transplantation of WT fat in lipodystrophic mice caused an enrichment of a Th17 cell mRNA signature when compared with lipodystrophic mice that received fat tissue from *ob/ob* mice (Supplemental Figure 7, G and H), underscoring the notion that fat-secreted leptin plays an important role in the differentiation of proinflammatory T cells. Likewise, we could detect a significantly higher number of colonic lamina propria Th17 cells in lipodystrophic mice receiving WT fat (Figure 6, G and H, and Supplemental Figure 7D), and we observed that the transplantation of WT fat significantly increased the production of IFN-γ in T cells of lipodystrophic mice (Figure 6H). Likewise, splenocytes from WT mice that were exposed to plasma of DSS-treated lipodystrophic mice that had been transplanted with WT fat showed a higher trend to flux calcium compared with those exposed to plasma from mice transplanted with *ob/ob* fat (Supplemental Figure 8). Taken together, these findings confirm previous observations that fat-secreted leptin controls the differentiation and function of T cells in mice (13). To exclude the possibility that the observed effects on T cell differentiation were primarily driven by differences in microbiota composition rather than by leptin, we performed 16S sequencing of stool obtained from the different experimental groups. These analyses did not reveal significant differences between lipodystrophic and WT controls, between DSS-treated lipodystrophic and WT mice, or between DSS-treated lipodystrophic mice transplanted with WT fat or *ob/ob* fat (Supplemental Figure 9).

To confirm that leptin is not only required for T cell homeostasis in mice but also involved in the functional regulation of proinflammatory CD4^+^ T cells in humans, we analyzed PBMCs from our patient with AGLCD, obtained before treatment with recombinant leptin and following 4 days of daily treatments with recombinant leptin. Consistent with our observations in mice, we found that leptin treatment increased the abundance and expression of IL-17A–producing CD4^+^ T cells in the patient with AGLCD in vivo (Figure 6I). Taken together, these findings demonstrate that fat-derived signals such as leptin are required for the differentiation and function of proinflammatory T cells in both mice and humans and that lipodystrophy strongly protects against intestinal inflammation.

### Identification of a persistently restricted TCR repertoire in the patient with AGLCD.

**Figure 7:**
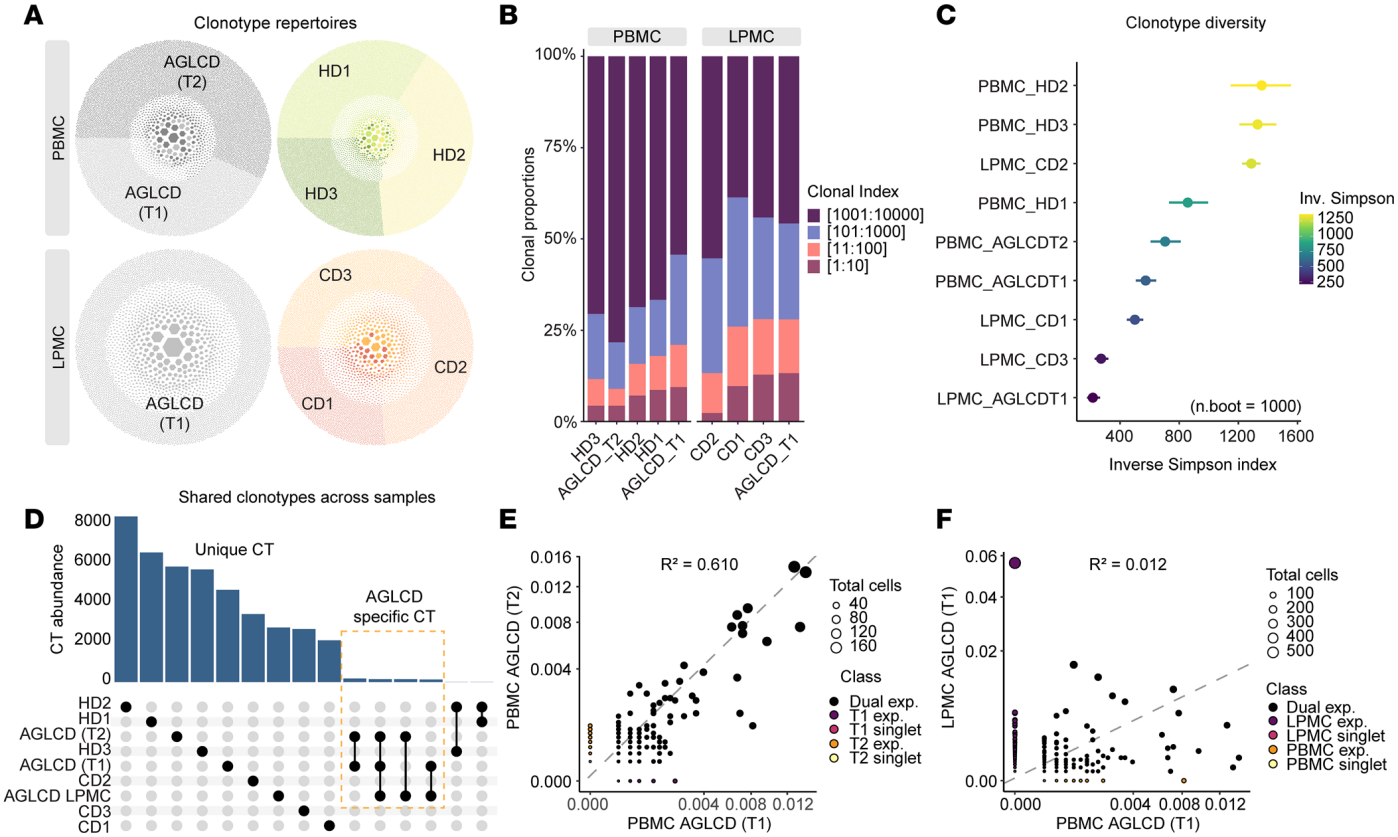
AGLCD patient TCR repertoires characterized by reduced diversity and persistent peripheral CTs. (**A**) Honeycomb plots from scTCR-seq of peripheral T cells from the patient with AGLCD at 2 time points, age-matched HDs, and intestinal T cells from the AGLCD and CD patients, showing CT distributions. (**B**) Stacked bar plot of proportional repertoire occupancy across groups. (**C**) Forest plot of mean inverse (Inv.) Simpson index (dots) after equal depth downsampling with 1,000 bootstrap iterations and 95% CIs (whiskers). (**D**) Upset plot of shared CTs across peripheral and intestinal samples from the patient with AGLCD, HDs, and patients with CD. (**E**) Scatter plots showing CT abundance concordance over time in AGLCD blood (2 years apart) and (**F**) between matched blood and intestinal samples. Clones are categorized by counts into singlets or expanded, either exclusively present or shared between the selected samples. Associations were evaluated using Pearson’s correlation; the corresponding R², reflecting explained variance, is shown along with a global dashed regression line. T1 exp., time point 1 expanded; T2 exp., time point 2 expanded.

Given the high frequency of CD4^+^ and CD8^+^ T cells that we had detected in both the periphery and at the site of inflammation in the patient with AGLCD, despite the absence of fat-derived signals, we hypothesized that the observed T cell expansion reflected a predominantly T cell–intrinsic process. To test this hypothesis, we characterized the T cell clonotypes (CTs) of the patient with AGLCD using scTCR-seq. scTCR-seq of PBMCs and LPMCs revealed marked clonal restrictions of T cells in both the blood and small intestine of the patient with AGLCD (Figure 7, A–C), in contrast to PBMCs from HDs and intestinal T cells from patients with CD. To assess the temporal stability and tissue distribution of these CTs, scRNA/TCR-seq was performed on PBMCs collected from the patient with AGLCD 2 years apart (time point 1 [T1] and time point 2 [T2]), while LPMCs were analyzed once from inflamed ileal tissue at the first time point. Cross-comparison of TCR repertoires across time points and tissues identified shared CTs in the patient with AGLCD (Figure 7D). Within the peripheral compartment, CT abundances were strongly correlated over time (Pearson’s *R²* = 0.61), indicating long-term persistence of expanded T cell clones. By contrast, the overlap between intestinal and peripheral repertoires was limited (Pearson’s *R²* = 0.012), suggesting that only a small subset of expanded clones was shared across compartments (Figure 7, E and F).

**Figure 8:**
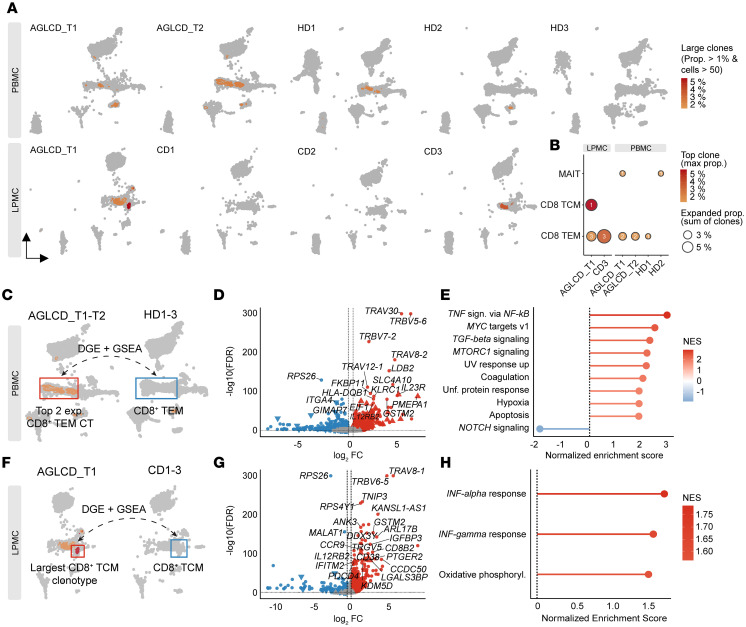
Characterization of clonally expanded T cells in the patient with AGLCD. (**A**) UMAPs showing densities of expanded CTs in the patient with AGLCD, HDs, and patients with CD. (**B**) Bubble plots quantifying top clone frequencies and total expanded CT proportions per sample. (**C** and **F**) Workflow diagrams outlining contrasts for DGE and GSEA by tissue. (**D** and **G**) Volcano plots of differentially expressed genes in expanded CD8^+^ T cells from the patient with AGLCD versus tissue-matched HD or CD T cells. (**E** and **H**) HALLMARK GSEA of expanded CD8^+^ T cells relative to tissue-matched control T cells. LPMCs and PBMCs were sequenced in independent scRNA-seq experiments. NES, normalized enrichment score; max prop., maximum proportion; Unf., unfolded.

Mapping dominant CTs (clonal proportion >1%, *n* >50) onto the scRNA-seq landscape demonstrated that clonal expansion was most prominent among effector and central memory CD8^+^ T cells and mucosal-associated invariant T (MAIT) cells (Figure 8, A and B). Tissue-matched differential gene expression (DGE) analyses revealed that clonally expanded T cells in both the blood and intestine exhibited a proinflammatory profile, with significant enrichment of gene signatures related to TNF-α, IFN-γ, and mTORC1 signaling (Figure 8, C–H). Together, these data identify persistently expanded, clonally stable inflammatory T cell populations in the circulation, with selective recruitment or retention of related clones within inflamed intestinal tissue.

### T cells of the patient with AGLCD harbor a somatic de novo NRAS G13D mutation.

**Figure 9:**
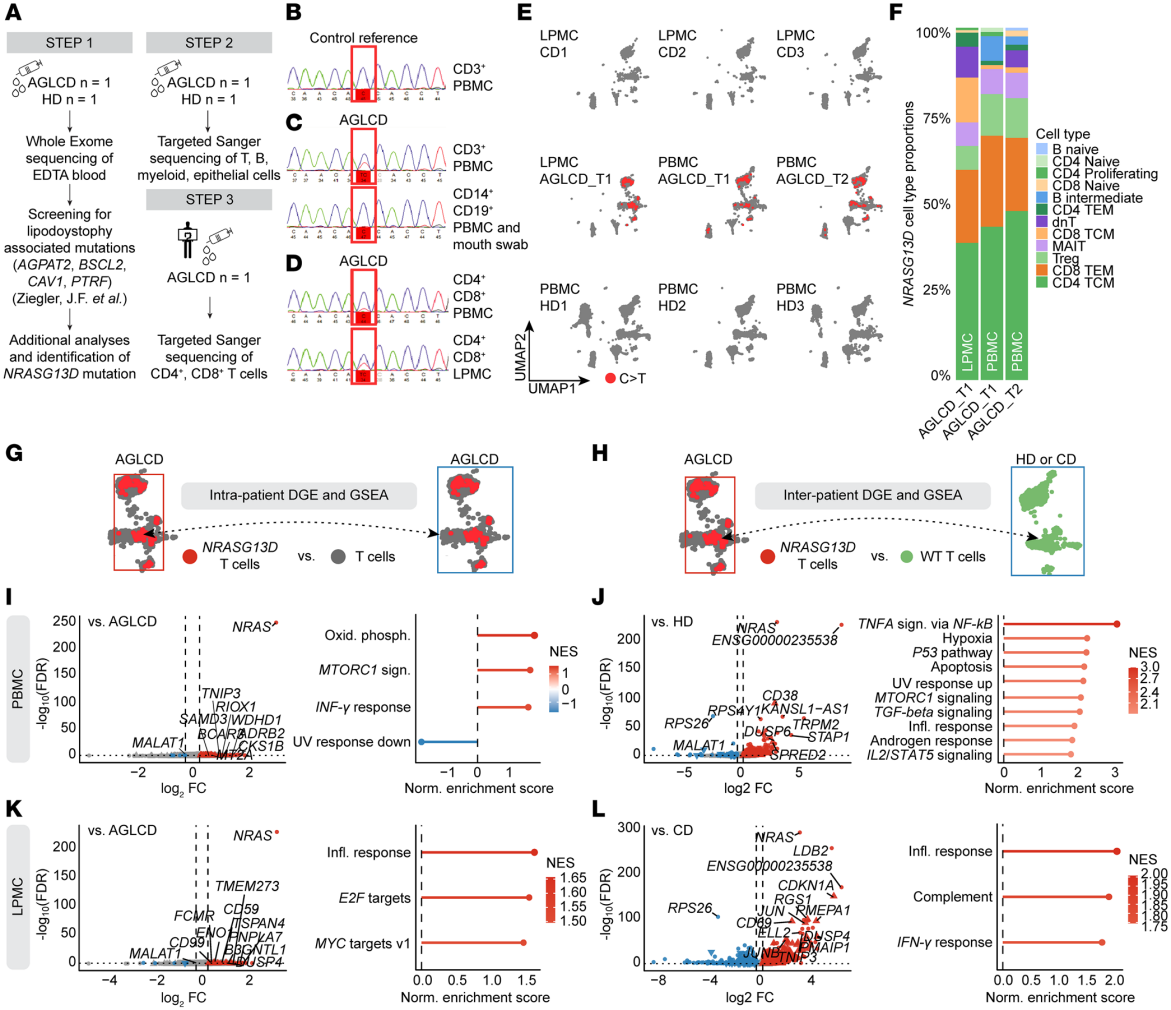
Detection of a somatic *NRAS* G13D mutation in T cells of the patient with AGLCD. (**A**) Workflow for WES experiments. (**B**–**D**) Targeted Sanger sequencing of the NRAS locus in buccal cells and sorted immune cell subsets from peripheral blood and ileal lamina propria of the patient with AGLCD. (**E**) UMAPs and (**F**) bar plots summarizing *NRAS* G13D^+^ cells across T cell subtypes per sample. (**G** and **H**) Workflow diagrams outlining intra-patient and inter-patient contrasts used for DGE analysis and GSEA. (**I** and **J**) Volcano plots comparing tissue-matched DGE and lollipop plots showing GSEA pathways comparing PBMC *NRAS* G13D^+^ T cells with T cells from the same patient with no detectable *NRAS* G13D reads (**I**) and T cells from HDs (**J**). (**K** and **L**) Volcano plots comparing tissue-matched DGE and lollipop plots showing GSEA pathways comparing LPMC *NRAS* G13D^+^ T cells with tissue-matched T cells from the same patient with no detectable *NRAS* G13D reads (**K**) and T cells from patients with CD (**L**). Infl., inflammatory; Oxid. phosph., oxidative phosphorylation; sign., signaling.

Prior research has indicated that somatic de novo mutations in T cells may play a role in the survival and evolution of clonally restricted T cells in patients with rheumatoid arthritis, multiple sclerosis, or nonmalignant hematologic or immunologic disorders, including aplastic anemia, myelodysplastic syndrome, and autoimmune thrombocytopenia (33–35). To understand whether the observed long-term persistence of clonally expanded T cells could be supported by the occurrence of somatic mutations in the patient with AGLCD, we next examined the mutational landscape of lymphocytes in this patient. Using whole-exome sequencing (WES) generated from whole blood of the patient with AGLCD and his relatives (11), we had previously excluded germline mutations commonly associated with inherited forms of generalized lipodystrophies in genes regulating lipolysis and adipocyte homeostasis (*AGPAT2*, *BSCL2*, *CAV1,* *PTRF*) (36–39). However, by looking beyond these genes and further reanalyzing this existing WES dataset (11), we were able to detect a de novo mutation causing a nonconservative glycine–to–aspartic acid substitution at codon 13 in the *NRAS* gene (*NRAS* G13D) (Figure 9A, STEP1). The *NRAS* gene encodes the NRAS protein, an essential regulator of the hematopoietic system development (40).

For example, NRAS was found to play a crucial role during the development and function of CD8^+^ T cells in mice, as NRAS-deficient CD8^+^ T cells show impaired thymic development and functional impairments in controlling infections with influenza virus (41). Previous studies have furthermore identified mutations that lead to the constitutive activation of NRAS as a potential risk factor for hematopoietic malignancies in both patients and transgenic mouse models (42). In particular, the *NRAS* G13D mutation results in the substitution of glycine at position 13 by an aspartate residue, which lies within the GTP-binding pocket of the NRAS protein. This variant has previously been implicated in the pathogenesis of autoimmune lymphoproliferative syndrome (ALPS) by rendering T cells and lymphocytes more resistant to growth factor deprivation–induced apoptosis that ultimately results in a systemic, nonmalignant lymphocyte expansion (15). In addition, *NRAS* G13D has been associated with RALDs, which are characterized by autoimmune manifestations such as lupus-like syndromes, lymphadenopathy, and massive splenomegaly (16–18). Moreover, the mutation has been identified in patients with Noonan syndrome and in cases of acute promyelocytic-like leukemia with concomitant myelodysplastic syndrome (43). Of note, the *NRAS* G13D variant is essentially absent as a germline allele, with an extremely low allele frequency of 6.196 × 10^–7^ (https://gnomad.broadinstitute.org) and has been shown to drive cell survival and lymphoproliferation in the context of ALPS (15). Since the *NRAS* G13D mutation was detected in less than 10% of all reads by WES performed on whole blood cells (data not shown), we hypothesized that only a small fraction of PBMCs might carry the *NRAS* G13D mutation. To test this, we used targeted Sanger sequencing to separately analyze peripheral CD3^+^ T cells, CD19^+^ B cells, CD14^+^ monocytes, and buccal epithelial cells isolated from the patient with AGLCD or a HD (Figure 9A, step 2). As shown in Figure 9, B–D, among all the cell types investigated, only CD3^+^ T cells harbored the *NRAS* G13D mutation. Further analysis of sorted CD4^+^ and CD8^+^ T cells isolated from either blood or intestinal biopsies from the patient with AGLCD (Figure 9E, step 3) confirmed the presence of the *NRAS* G13D point mutation in T cells in the peripheral blood compartment as well as in FACS-sorted T cells at the site of inflammation in the intestine (Figure 9E). Remarkably, we were also able to detect RNA reads spanning the *NRAS* G13D mutation in our scRNA-seq data in CD4^+^ and CD8^+^ T cells obtained from the peripheral blood and intestine, supporting the notion that the patient with AGLCD carries a somatic de novo *NRAS* G13D mutation in the T cell compartment (Figure 9F). To assess the transcriptional consequences of the *NRAS* G13D mutation, we compared gene expression profiles of mutation-positive T cells with T cells from the same patient lacking detectable *NRAS* G13D, as well as with T cells from HDs expressing WT NRAS (Figure 9, G and H). *NRAS* G13D^+^ T cells showed increased NRAS transcript abundance and a marked enrichment of lymphoproliferative and inflammatory gene programs, supporting the idea of a functional role for activating NRAS signaling in driving T cell expansion and sustained lymphoproliferation (Figure 9, I–L).

In light of the growing evidence for the role of somatic mutations in the evolution and maintenance of clonally restricted T cells in nonmalignant T cells, our data collectively indicate that the occurrence of the *NRAS* G13D mutation might facilitate the survival of autoreactive T cells, thereby advancing the selection and survival of clonally restricted inflammatory T cells in the patient with AGLCD, ultimately contributing to persistent intestinal and systemic inflammation, which can then be further aggravated by exogenous substitution with recombinant leptin.

## Discussion

In summary, we describe T cell–intrinsic lymphoproliferation characterized by a heterozygous de novo mutation of *NRAS* as a potential facilitator of systemic and intestinal inflammation in AGL. A T cell–driven cause of AGL is supported by observations in patients with cancer who develop AGL after treatment with the immune checkpoint–blocking antibodies pembrolizumab and nivolumab, resulting in adipose tissue infiltration by CD8^+^ T cells, progressive generalized lipodystrophy, severe insulin resistance, and chronic inflammation (4–6).

Using mouse models of lipodystrophy, we were able to exclude the possibility that the observed lymphoproliferation in AGLCD was a secondary consequence of absent fat-derived adipokine signaling, as we did not observe spontaneous development of lymphoproliferation in lipodystrophic mice. In contrast, we demonstrated that lipodystrophy protected mice from chronic DSS-induced intestinal inflammation by reducing the differentiation of proinflammatory T cell subsets, especially Th1 and Th17 cells, impairing the capacity of CD4^+^ and CD8^+^ T cells to flux calcium and by improving intestinal barrier properties without significantly altering the gut microbiota. Conversely, the transplantation of allogeneic fat tissue resulted in a decreased expression of TJ proteins such as Cldn3 and angulin 1/LSR and promoted the differentiation of proinflammatory T cells in a leptin-dependent manner, ultimately exacerbating intestinal inflammation. These results align with our prior observations, in which we noticed a worsening of intestinal inflammation in the patient with AGLCD after treatment with recombinant leptin, which eventually necessitated aggressive triple-immune suppression using tacrolimus, mycophenolate mofetil (MMF), and the TNF blocker adalimumab for the treatment of inflammation (11). In summary, our findings suggest that leptin acts as a proinflammatory adipokine in AGLCD, amplifying systemic and intestinal inflammation and thereby reinforcing a disease process characterized by T cell–intrinsic lymphoproliferation of inflammatory CD4^+^ and CD8^+^ T cells. It is important to note, however, that only 1 patient with AGLCD could be studied because of the extreme rarity of this condition, and further studies will be required to validate and extend these observations beyond intestinal inflammation.

Given the previously reported role of *NRAS* G13D in the promotion of T cell lymphoproliferation in patients with ALPS (15) and RALD and the restricted TCR repertoire in T cells of the patient with AGLCD, it seems very likely that the presence of *NRAS* G13D facilitates the survival and/or clonal evolution of inflammatory T cells, thereby promoting systemic and intestinal inflammation. In this study, we were able to detect clonally restricted cells that carry the *NRAS* G13D mutation, express inflammatory markers, and display significant enrichment of gene sets related to mTORC1 signaling, which is a crucial pathway for maintaining immune receptor signaling, effector functions, and metabolic homeostasis of T cells (44). Taken together, these findings suggest that the occurrence of somatic mutations in clonally restricted cells might contribute to the persistence of clonally restricted T cell subsets in AGLCD over time. Likewise, Lundgren and colleagues recently identified an *NRAS* G13D mutation as a potential driver of clonal T cell expansion in a patient with aplastic anemia (33), which is supported by observations from Garcia and colleagues, who demonstrated that naturally occurring somatic mutations can enhance the function and survival of CAR T cells in vitro and in vivo (45). Moreover, clonally restricted T cells were found to harbor somatic mutations in up to 20% of patients with other autoimmune diseases such as rheumatoid arthritis or multiple sclerosis (34, 35). However, speculation remains as to which antigens are recognized by clonally expanded T cells found in the patient with AGLCD, and future studies will be required to delineate how somatic mutations such as the *NRAS* G13D mutation contribute to the selection of antigen-specific inflammatory cells under suboptimal conditions such as hypoxia and inflammation, in both AGL and autoimmune diseases in general.

A T cell–driven mechanism in AGL is also supported by Fischer-Posovszky and colleagues, who previously reported that T cells induced apoptosis of adipocytes in a FASL-dependent manner in a young patient with autoimmune-associated AGL (46). Despite this observation, the specific antigens responsible for T cell activation in the adipose tissue of patients with AGL are unknown, and it remains speculative if the T cells found in the periphery and gut of the patient with AGLCD could also contribute to adipose tissue destruction, as we were unable to isolate fat-residing T cells from the patient with AGLCD because of the patient’s complete absence of adipose tissue. However, given the established role of T cells in chronic systemic inflammation in AGL, it is, in our opinion, still plausible to hypothesize that the observed T cell proliferation may drive both autoimmune lipodystrophy and intestinal inflammation in the patient with AGLCD. Similarly, concomitant local inflammation of adipose tissue such as panniculitis and erythema nodosum (47) can also be observed in CD patients with extraintestinal manifestations, suggesting that the complete loss of fat tissue in the patient with AGLCD might also be viewed as a very extreme form of an extraintestinal manifestation of CD.

Additionally, our data caution that the de novo mutation in *NRAS* may facilitate the transformation of lymphoproliferative T cells into malignant lymphoma, given the high incidence of T cell lymphoma observed in patients with AGL (3). This is particularly important because mutations in *NRAS* are associated with therapy resistance and unfavorable clinical outcomes in patients with cutaneous T cell lymphoma (48). One limitation of this study stems from the prolonged disease course of our patient with AGLCD and the intensive immunosuppressive therapy with tacrolimus, MMF, and TNF blockers. As a result, it remains speculative whether the somatic *NRAS* G13D mutation was a consequence of the harsh immunosuppressive therapy, the patient’s severe inflammation, or a fundamental driver in the development of AGLCD. Additionally, we cannot exclude the possibility that the triple immunosuppressive therapy further influenced our scRNA-seq, mass cytometry, and flow cytometry findings. Despite extensive efforts, we have so far not been able to identify additional patients with AGLCD, owing to the unique coexistence of acquired generalized lipodystrophy and CD. Consequently, it remains unclear whether the somatic mutations observed in T cells represent a patient-specific feature of AGLCD or a more general characteristic of AGL in the context of concomitant inflammatory disorders. Another factor that should be considered as a potential contributor to genetic instability and the subsequent formation of somatic mosaicism is the high lipid burden and the consecutive lipotoxic challenge that lymphocytes might experience during AGL as a result of systemically elevated triglyceride levels. We previously observed in the patient with AGLCD that, in the absence of adipose tissue monocytes, NK and CD8^+^ T cells stored high amounts of lipid droplets (11). It is thus conceivable that the elevated levels of circulating lipids observed in AGL may contribute to lipotoxicity and subsequent genetic instability, thereby not only supporting the formation of somatic mutations in inflammatory nonmalignant T cells, as evidenced in the current study, but also increasing the overall probability of genetic alterations, ultimately leading to an increased risk for lymphoma development in AGL.

Despite recent advances in sequencing technologies, somatic mutations may often be missed by standard bulk sequencing approaches, as mutated cell lines often represent only a small fraction of cells in tissue conglomerates or whole blood samples. We believe that it will be critical to thoroughly consider single-cell analyses to delineate the intra-individual genetic heterogeneity driving complex diseases such as AGL and to develop individualized therapies based on these analyses. It is currently unknown whether somatic de novo mutations may be a specific feature of AGLCD or whether similar de novo mutations may underlie the development of IBD in general, and future studies are needed to explore these questions.

We believe that our data are not only crucial in the context of AGL, as they show that chronic inflammation in AGL is likely to arise from T cell–intrinsic events such as somatic mosaicism and clonal evolution of inflammatory cells, but they also may help to better delineate the so far incompletely understood function of adipose tissue in the pathogenesis of IBD. Although findings from cross-sectional and retrospective cohort studies correlating the effect of adipose tissue with the development of intestinal inflammation have indicated that obesity is associated with a higher risk of developing CD, the underlying mechanisms of this correlation remain unsolved (9). Our data support the proinflammatory role of adipose tissue and its secreted factors in intestinal inflammation and confirm that adipose tissue-derived signals, and especially leptin, play an important role in the regulation of immune cell function by controlling the differentiation and function of inflammatory T cells. These findings suggest that the removal or reduction of adipose tissue with a subsequent reduction in adipokine levels may improve intestinal barrier function and reduce the generation of proinflammatory T cells such as Th17 and Th1 in patients with relapsing IBD. Overall, this study reveals a complex picture of the interplay between adipose tissue and immune cell homeostasis and argues that tight regulation of fat-derived signals is required to prevent excessive autoimmune responses in IBD. In addition, our findings indicate that somatic mutations in T cells might contribute to the development of complex autoimmune phenotypes and that somatic mutations should be considered in the future as potential drug targets in personalized treatment approaches for complex IBD with severe extraintestinal manifestation.

## Methods

### Sex as a biological variable.

Our study used human tissues and primary cells from both male and female patients. HDs were sex matched to the patient with AGLCD to eliminate any potential confounding effects related to sex. In the lipodystrophic mouse model, both male and female mice were used to reduce animal breeding.

### Isolation of peripheral and LPMCs.

PBMCs were isolated from the patient with AGLCD and HDs using SepMate-50 tubes (STEMCELL Technologies) according to the manufacturer’s instructions. Surgical specimens of the terminal ileum were obtained from patients with CD and the patient with AGLCD undergoing colectomy. Noninflamed specimens from patients with colorectal cancer served as controls. The main clinical patient characteristics are summarized in Supplemental Table 1. Isolation of LPMCs was done as previously described (19). For details, see the Supplemental Methods.

### Mass cytometry staining and acquisition.

LPMCs isolated from ileal resections were stimulated for 4 hours in activation medium (Supplemental Table 2) as previously described (19). A complete list of antibodies is provided in Supplemental Table 3. Staining, acquisition, and data analyses protocols are described in detail in the Supplemental Methods.

### WES.

DNA was extracted from sorted immune cells using DNeasy Blood and Tissue Kits (Qiagen). Exomes were enriched and sequenced as previously reported (11). For details, see the Supplemental Methods.

### scRNA-seq.

FACS-sorted CD45^+^ single-cell suspensions were loaded onto a Chromium Chip G (10x Genomics) according to the manufacturer’s instructions for processing with the Chromium Next GEM Single Cell 5′ Library and Gel Bead Kit version 1.1. Details on library preparation and analyses are provided in the Supplemental Methods.

### Mice.

Lipodystrophic *Pparg^fl/fl^ Adipoq-Cre* mice were a gift from Ulrich Kintscher (Charité, Berlin, Germany). Lipodystrophic mice and littermate controls were bred at the Charité animal facility. Leptin-deficient *ob/ob* mice were purchased from Charles River Laboratories.

### Chronic DSS colitis.

Nine- to 13-week-old *Pparg^fl/fl^ Adipoq-Cre* mice and their control littermates were exposed to 3 cycles of DSS (MP Biomedicals), with each cycle consisting of 5 days of treatment with 1.5% DSS in the drinking water followed by 9 days of plain water. Mice were monitored daily for weight loss and stool consistency, as indicated in the Supplemental Methods.

### Adoptive fat transplantation.

Fat transplantations were performed on *Pparg^fl/fl^ Adipoq-Cre* mice and control littermates at steady state or after 1 cycle of DSS treatment. A total of 500–600 mg parametrial donor fat was harvested from sex-matched WT littermates or *ob/ob* mice. Mini-laparotomy was performed on anesthetized recipient mice to install donor fat into their peritoneal cavity, as previously described (49). One month later, at sacrifice, grafts were weighed and evaluated for attachment and vascularization using binocular surgical microscopy.

### Histopathologic analyses.

Histopathologic analyses of colon sections from mice after chronic DSS-induced colitis with or without adoptive fat grafting were performed in a blinded fashion using an additive scoring system ranging from 0 to 21 points as previously described (50).

### Flow cytometry.

Cells were incubated for 4 hours with or without activation medium (Supplemental Table 2) and 10 μg/mL brefeldin A (MilliporeSigma) 2 hours before harvesting. Cells were stained for viability followed by surface staining before being fixed in fixation/permeabilization buffer (eBioscience) according to the manufacturer’s protocol and stained with intracellular antibodies. A complete list of antibodies and viability dyes is provided in Supplemental Table 5. Samples were acquired using a BD FACSCanto II device. Data were analyzed using the FlowJo software version 10.6.2.

### Bulk RNA-seq.

Distal colon tissue was homogenized for 30 seconds at 50 Hz in lysis buffer (RLT buffer plus β-mercaptoethanol) (Qiagen) and Reagent DX (Qiagen) using a TissueLyser LT Adapter. RNA was extracted following the RNeasy Mini protocol (Qiagen) according to the manufacturer’s instructions. For details on sequencing and data analysis, see the Supplemental Methods.

### ELISA.

Plasma levels of leptin or anti–*E. coli* (LPS) IgG were determined using the mouse Leptin DuoSet ELISA kit (R&D Systems) or the mouse anti–*E.* *coli* LPS (O111:B4) Antibody ELISA Kit (Chondrex). For details, see the Supplemental Methods.

### Western blot analyses and Ussing chamber experiments.

All buffers used for immunoblotting are listed in Supplemental Table 6. For details on experimental procedures, see the Supplemental Methods.

### Calcium influx measurements in mouse splenocytes.

Splenocytes from lipodystrophic or littermate controls were stained with Fluo-4, kept in 0 mM Ca^2+^ Ringer solution, stimulated with 1 μg thapsigargin after 30 seconds, and subsequently exposed to 2 mM Ca^2+^ Ringer solution for 2 minutes as previously described (19). Change of fluorescence over time was acquired using a BD FACSCanto II flow cytometer. For details on buffers and reagents, see the Supplemental Methods.

### Data availability.

Patient data supporting the human findings of this study are available from the corresponding authors upon reasonable request. Mouse bulk sequencing data for this project can be found on the European Nucleotide Archive under reference number PRJEB100523. Human raw sequencing data are available from the European Genome-phenome Archive under controlled access accession codes EGAD50000002190 (scRNA-seq) and EGAD50000002191 (scTCR-seq). Data values for all graphs are provided in the Supporting Data Values file.

### Statistics.

Statistical analysis was performed using GraphPad Prism 10 (GraphPad Software) or coded into R using RStudio (versions 4.3.1 and 4.3.2). Statistical significance was calculated by Fisher’s exact test with the Benjamini-Hochberg correction for multiple comparisons, unpaired 2-tailed *t* test with Welch’s correction, Kruskal-Wallis test with Dunn’s correction for multiple comparisons, 2-way-ANOVA, 1-way-ANOVA with Šidák’s corrections, Mann-Whitney *U* test, or repeated-measures correlation. *P* values of less than 0.05 were considered statistically significant. Data were presented as a mean + SEM when applicable; otherwise, they were presented as box and whisker plots where boxes range from the 25th to 75th percentiles. Whisker plots show the minimum (smallest) and maximum (largest) values while the line in the box indicates the median.

### Study approval.

Written informed consent was obtained from all healthy volunteers and patients as approved by the IRB of Charité – Universitätsmedizin Berlin (EA1/200/17). All mouse experiments were performed according to protocols approved by Berlin’s Regional Animal Experimentation Committee (LaGeSo).

## Author contributions

The study was designed by ML, TO, PW, ADS, BS, and CW. Supervision and main funding acquisition was provided by ADS, BS and CW. The original draft of the manuscript was prepared by ML, TO, PW, ADS, BS, and CW. ML, TO, PW, MOJ, CB, RG, FT, ADS, BS, RGB, and CW edited and revised the manuscript. BSL, JFZ, PA, ER, BW, MS, KM, AF, BS, and CW contributed to the acquisition of patient material and to the interpretation of clinical and experimental data. Data were visualized by ML, TO, and PW. Bioinformatics analyses and data interpretation were led by PW and supported by BSL, BO, DB, CSNK, AF, and ADS. CyTOF and flow cytometric analyses were performed by ML and TO (lead) and JFZ, DK, RG, and JH (supporting). Animal models of colitis experiments were conducted by ML and TO (lead); MOJ, SK and IF, JH, RGB, and FT (supporting). 16S sequencing and analysis were conducted by SW. Western blot analyses and Ussing chamber experiments were performed by SMK. Histologic analyses were performed by AK, ML, and TO. Single cell sequencing was preformed by TC. All authors revised the manuscript and approved the final version submitted for publication.

## Funding support

- CW received funding from the German Research Foundation (Project ID: We 5303/3-2).
- The following individuals are funded by the German Research Foundation as part of the SFB-TRR241 (Grant ID: 375876048): BS and CW for project B01; AS and CW for project A09; CB and SW for project A03; CSNK for project B05; SK for B06 and BS for project Z02.
- The following groups are funded by the German Research Foundation as part of the SFB-TRR 412 (Grant ID: 535081457): BS and CW for project A05; and FT for project B02.
- BS received funding from the German Research Foundation for the following projects CRC 1449-B04 (Grant ID: 431232613); CRC 1340-B06 (Grant ID: 372486779); SI749/14-1 (Grant ID: 418055832).
- DB, FT, CSNK, ADS, BS, and CW received funding under Germany’s Excellence Strategy- EXC3118/1 (Project ID: 533770413).
- CW and JZ received funding from the Clinician Scientist Program of the Berlin Institute of Health.
- CW received funding by the Fritz-Thyssen Foundation (10.19.2.028MN).

## Supplementary Material

Supplemental data

Unedited blot and gel images

Supporting data values

## Version 1 03/16/2026

Electronic publication
