# Supplementary material for: Characterization of intestinal immune responses in generalized human and murine lipodystrophy
Source: J Clin Invest. 2026 Mar 16;136(6):e192322. doi: 10.1172/JCI192322 (PMC12987653; doi:10.1172/JCI192322)
Supplement: Supplemental data [file jci-136-192322-s254.pdf]

Supplemental Materials Letizia et al.

Supplemental Experimental Procedures

Isolation of lamina propria mononuclear cells (LPMCs) from ileal resectates
Ileal mucosa of the AGLCD Patient, CD Patients or non-inflamed controls was dissected, washed in 1 mM 1,4 dithiothreitol (DTT) (#6908.4, Carl Roth), and dissolved in Hanks' balanced salt solution (HBSS) w/o Ca^{2+} and Mg^{2+} (Thermofisher Scientific). Lamina propria tissue was cleaned of any left submucosa, cut into pieces, and washed three times in 1 mM EDTA (#E6511, Sigma-Aldrich) for 15 min at 37 °C in shaking condition at 250 rpm. The EDTA was then washed out with HBSS without Ca^{2+} and Mg^{2+} (Thermofisher Scientific), and the tissue was incubated in a digestion medium supplemented with 0.15 mg/mL collagenase A (Roche) for 16 hours at 37 °C in shaking condition at 200 rpm (**Supplemental Table 2**). On the following day, the supernatant was filtered through a 100 µm cell strainer (Thermofisher Scientific) and washed three times in HBSS without Ca^{2+} and Mg^{2+}. Cells were separated by centrifugation on a Percoll gradient (GE Healthcare) at 300 g for 30 min at 4 °C. Lymphocytes and monocytes were isolated from the 40-60% Percoll (GE Healthcare) interface, washed twice, and used for downstream assays.

Cell stimulation and mass cytometry staining
LPMCs were ex-vivo stimulated with ionomycin/PMA for 4h (see **Supplemental Table 2** for information on media used for activation) and 10 µg/mL brefeldin A (Sigma-Aldrich) was added to all samples for the last 2 h of stimulation, while 25 KU benzonase nuclease (Sigma-Aldrich) was added (1:10,000) 15 min before harvesting. LPMCs were then fixed in Smart Tube buffer (SMART TUBE) supplemented with 20% BSA (Sigma-Aldrich) and subsequently stored at -80 °C until antibody staining and acquisition. On the day of acquisition, single cell suspensions were thawed, and to pool multiple samples in single batch/acquisition run (max 40 samples per batch/run) to avoid introducing batch effects, LPMCs samples were pooled and barcoded for 30 min at room temperature (RT) using the Cell-ID 20-plex Pd Barcoding Kit (Fluidigm) ± CD45-89Y staining. Individual samples were washed twice with cell staining buffer (Fluidigm) and pooled before staining. Anti-

human antibodies were either purchased pre-conjugated to metal isotopes or conjugated in-house using the MaxPar X8 conjugation kit (Fluidigm). A full description of antibodies used is provided in **Supplemental Table 3**. Cells were incubated for surface staining for 30 min at 4 °C, washed twice with cell staining buffer, and incubated in fixation/permeabilization buffer (eBioscience) for 60 min at 4 °C. After two washes with permeabilization buffer (eBioscience), cells were stained with an antibody cocktail against intracellular molecules for 1 hour at 4°C. Cells were then washed twice with permeabilization buffer and incubated overnight in 2% methanol-free formaldehyde solution (Thermofisher Scientific) for 1 hour at room temperature. LPMCs were then washed and resuspended in 1 mL of iridium intercalator solution (Fluidigm) for 1 hour at RT. Finally, cells were washed twice with cell staining buffer and then twice with double-distilled water (ddH$_2$O) for time-of-flight (CyTOF) mass cytometry measurements. Cells were acquired using a CyTOF2 upgraded to Helios specifications (Fluidigm), with software version 6.5.236.

Analysis of mass cytometry data

The Cytobank software package (www.cytobank.org) was used for the initial manual gating of single cells and the de-barcoding of pooled samples. FCS files of de-barcoded single cells were exported and further compensated for signal spillover using the R software (3.6.0, Bioconductor 9) (www.R-project.org) and by applying the CATALYST package and arcsinh transformation (scale factor 5) prior to data analysis (1). Compensated files were then gated for CD45$^+$ cells (**Supplemental Figure 1**), and t-distributed stochastic neighbor embedding (t-SNE) maps were generated according to the expression of lineage and/or functional markers. T-sne maps were generated according to the expression of linage markers for T (CD4, CD8, CD45RO, CD45RA, CCR7, IL-7R, CD27, CD25, PD-1, perforin), B (CD38, CD19), NK (CD56) and myeloid cells (CD11b, CD11c) (**Figure 1C, D**). For all analyses, FCS files with t-SNE embedding as two additional parameters were exported from Cytobank. Clustering analysis was performed using the *FlowSOM/ConsensusClusterPlus* package in R (3.6.0, Bioconductor 9) (2). Cell clusters of LPMCs were identified after visual inspection of t-SNE plots and functional interpretation of heat maps generated by FlowSOM/ConsensusClusterPlus.

Statistical analysis of differential abundance (DA) clusters between disease groups was performed using a generalized linear mixed effects model (GLMM) available through the R package diffcyt (using all defaults with analysis_type = ”DA,” method_DA = ”diffcyt-DA-GLMM,” min-cells = 3). The false discovery rate (FDR) was adjusted to 10% using the Benjamini-Hochberg (BH) procedure for multiple hypothesis testing, as previously described (3). Multiple t-test (Benjamini, Krieger, and Yekutieli two-stage linear step-up procedure, with Q = 1%) was used to analyze protein expression levels among disease groups.

Whole exome sequencing

PBMCs were obtained from the AGLCD Patient and DNA was extracted from PBMC subsets or buccal swaps using DNeasy Blood & Tissue Kits (#69504, Qiagen, North Rhine-Westphalia, Germany). Exome sequencing was performed by the Institute of Clinical Molecular Biology, Kiel, Germany, using the xGen Exome Research Panel v 1.0 and 2 × 75 bp paired-end, while the sequencing was carried out on an Illumina HiSeq 3000 (Illumina), as previously reported (4, 5). Reads were mapped to the human reference genome assembly hg19 using BWA (6). They were then sorted, converted to bam format, and indexed using SAMtools (6). This was followed by PCR duplicate removal (http://picard.sourceforge.net) and local realignment around InDels followed together with base quality score recalibration using GATK according to their best practice recommendations, in accordance with variant calling and variant quality score recalibration. Finally, Alissa Interpret (Agilent) was used for Variant annotation and filtering. Exome enrichment was performed by the Center for Molecular Biosciences (ZMB), Kiel, Germany, using the xGen Exome Research Panel v 1.0 and 2 × 75 bp paired-end, while the sequencing was carried out on an Illumina HiSeq 3000 (Illumina). Reads were overlapped to the human reference genome assembly hg19 using BWA, sorted, converted to bam format, and indexed using SAMtools. This was followed by PCR duplicate removal and local realignment around InDels followed together with base quality score recalibration using the GATK. Finally, Alissa Interpret (Agilent) was used for Variant annotation and filtering.

Single cell RNA sequencing

PBMCs or LPMCs were collected from the AGLCD patient, 3 healthy donor or 3 Crohn's disease patients as indicated and resuspended in 1X PBS (#10010023, Gibco) containing 2% FBS (Sigma-Aldrich) and 1 mM EDTA (#E6511, Sigma-Aldrich) at a density of 5 x 10^7 cells/mL. Single cell suspensions were loaded onto a Chromium Chip G (10x Genomics, Pleasanton, California) according to the manufacturer's instructions for processing with the Chromium Next GEM Single Cell 5' Library and Gel Bead Kit v1.1. 15,000 cells were loaded for each reaction. TCR single cell libraries were subsequently generated from the same cells using the Chromium Single Cell V(D)J Enrichment Kit, Human T Cell (10x Genomics). Libraries were sequenced on the Illumina NovaSeq 6000 machine (Illumina) using 2x100 bp for gene expression, aiming for 50,000 reads per cell and 2x150 bp and 5000 reads per cell for TCR libraries. Raw FASTQ files were processed with Cell Ranger v9.1.0 (10x Genomics) using the GRCh38-2024-A reference for gene-expression (GEX) libraries and vdj_GRCh38_alts_ensembl-7.1.0 for VDJ libraries. Ambient RNA contamination was corrected using CellBender remove-background v0.3.2, applied to raw count matrices before downstream QC. Per-sample 10x 5′ scRNA-seq matrices were imported into Seurat v5.2.1 (R) and QC metrics annotated using scCustomize v3.1.4. Cells were retained if they expressed >800 and <5,500 genes, contained >500 UMIs, and had <20% mitochondrial transcripts. Data were then log normalized, variable features selected using VST (nfeatures = 2000), and scaled. Putative doublets were identified per sample using DoubletFinder v2.0.4 with pN = 0.25, pK optimized via paramSweep, and a 7.5% expected doublet rate. Filtered objects were merged and then mapped to the human 10k PBMC reference using Azimuth weighted-nearest-neighbor embeddings (RunAzimuth, reference = "pbmcref"). Differential cell-type abundances were tested on arcsine–square-root–transformed per-sample proportions using propeller.ttest and limma (speckle v1.2.0) with empirical-Bayes variance moderation (robust = TRUE, trend = FALSE). Effects with 95% confidence intervals were reported, and significance defined at FDR < 0.05. Clonotype repertoires were visualized with enclone v0.5.219 and

clonotypes defined using the CTstrict criterion in scRepertoire v2.5.2, requiring identical CDR3 amino acid sequences and V/J gene usage within each chain (TRA/TRB). Clonotype expansions were defined using the Large expansion category in scRepertoire, corresponding to highly expanded clones that additionally contained more than 50 cells per clonotype. Differential gene expression analyses were performed in Seurat (v5.2.1) using FindMarkers (Wilcoxon rank-sum test; logfc.threshold = 0, min.pct = 0.05), retaining genes with BH-adjusted FDR < 0.05 and absolute $\log_2$ fold change ≥ 0.25. Gene lists were ranked by $\log_2$ fold change and subjected to gene set enrichment analysis using fgsea v1.30.0 with MSigDB HALLMARK pathways (minSize = 10, maxSize = 500), reporting normalized enrichment scores and adjusted P values. All DGE and GSEA analyses were tissue- and cell-type-matched and performed at the clonotype/condition-pseudobulk level where appropriate. To quantify NRAS G13D variant-supporting reads per cell, BAM files were split by cellular barcode and base pileups at the locus generated with samtools mpileup (-r chr1:114716123-114716123 -q 10 -Q 10) and cells counted as mutated with at least one read supporting the C>T substitution. All random operations used set.seed(1234).

Stool scoring during mouse DSS treatment

Stool consistency was determined as follows: score 0, normal (solid pellet); 1, soft but in pellet shape; 2, loose stool but with some solidity; 3, loose stool with signs of liquid consistency; 4, watery diarrhea. Rectal bleeding was scored as follows: score 0, no sign of blood; score 1, no bleeding; 2, slight bleeding; 3, bloody diarrhea; 4, gross bleeding (7).

Processing of mouse spleens and mesenteric lymph nodes for single cell suspension

Spleens and mesenteric lymph nodes from lipodystrophic mice and control littermates were disassociated and filtered through a 70 μM cell strainer (Thermofisher Scientific) using MACS buffer consisting of PBS (Gibco) supplemented with 0.5% Fraction V bovine serum albumin (BSA) (Sigma-Aldrich). For red blood cell (RBC) lysis, cells were resuspended in RBC lysis buffer (8.9 g NH_4Cl, 1 g $KHCO_3$, 0.038 g EDTA, 1 L distilled water) for 2 min at RT, then washed and filtered again through a 40 μM cell strainer (Thermofisher Scientific). Alternatively, terminal ileum and colon sections were opened

longitudinally and cut into small pieces for two washes in 1 mM EDTA solution (Sigma-Aldrich) for 15 min at 37 °C under shaking conditions at 250 rpm. Tissue pieces were then digested in complete media supplemented with 200 U/mL Collagenase D (Roche) for 90 min under shaking conditions (250-300 rpm, 37 °C). Cells were then subjected to a 40/100% discontinuous Percoll gradient (GE Healthcare), 1200 g for 25 min at RT, to obtain mononuclear cell fractions. Finally, cell pellets were washed and resuspended for activation at 37 °C for 4 hours in RPMI medium 1640 (1X) (Gibco), supplemented with 10% fetal bovine serum (FBS) (Sigma-Aldrich), 10% penicillin-streptomycin (Thermofisher Scientific), 50 µM 2-mercaptoethanol (Gibco). Single-cell solutions were then stained with antibodies listed in **Supplemental Table 5** and further processed using standard FACS-protocols.

Stool 16S sequencing

10 ng of stool genomic DNA was used in 25 cycle PCR amplification (NEBNext Q5 Hot Start Hifi PCR Master Mix, New England Biolabs) of genomic 16S ribosomal RNA V4 regions using the prokaryotic primer pair (515F forward primer: 5'-GTGYCAGCMGCCGCGGTAA−3'; 806R reverse primer: 5'-GGACTACNVGGGTWTCTAAT−3') containing 12 bp barcodes on the forward primer (https://earthmicrobiome.org/protocols-and-standards/16s/). PCR products were purified with AMPure XP Beads (Beckmann Coulter), pooled in equimolar ratios and analysed by 2 × 151 paired-end sequencing on an Illumina MiSeq device. Raw fastq files were then imported and analysed in QIIME2 v2025.4 with DADA2 as the method for quality control, dereplication and amplicon sequence variant (ASV) table generation. The SILVA small subunit database release 138.2 was used at a 99% similarity cut-off for taxonomic classification. ASV and taxonomic tables were imported into R (version 4.5) as a phyloseq object and the “vegan” (2.6.4) package was used for diversity analysis and ordination. Ggplot2 was used for generation of graphical illustrations.

Bulk RNA sequencing

Total RNA was sequenced on an Illumina platform. HISAT2 (8) was used to perform alignments to the Mus musculus reference genome assembly GRCm38 (mm10) after data quality control and filtering. After gene expression quantification,

the differential gene expression analysis was performed using DESeq2 software, and significant differences were considered if |log2(FoldChange)| $\geq$ 1 & padj $\leq$ 0.05. Downstream pathway and gene enrichment analyses were performed using the g:Profiler platform (9) or the GSEA 4.3.2 software (9).

Ussing chamber experiments

Voltage and transepithelial resistance (TER; R^t) of murine colonic mucosal samples were measured in Ussing chambers with an area of 0.049 cm². Resistance of bathing solution and filter was measured prior to each experiment and subtracted. Ussing chambers and water-jacketed gas lifts were filled with 10 ml standard Ringer's solution, which was equilibrated with 5% CO_2 and 95% O_2 at 37 °C. Potential changes were recorded when the solution of one hemichamber was changed to a solution containing a reduced concentration of NaCl and all other components identical to standard Ringer's; osmolality was balanced by mannitol. Sodium and chloride permeabilities were calculated from the resulting dilution potentials and the Goldman-Hodgkin-Katz equation as previously reported (10, 11).

Flux measurements

Dextran fluxes were measured in 5 ml circulating Ringer's solution under short-circuit conditions. 50 µL of 40 mM dialyzed fluorescein isothiocyanate-labeled dextran (4-kDa FITC-dextran; #60842-46-8, TdB Labs A B) were added to the mucosal side and unlabeled dextran of the same concentration to the serosal side. Serosal samples (300 µL) were taken and replaced by fresh solution at 0, 15, 30, 45, and 60 min. Tracer fluxes were calculated from the collected samples measuring the concentrations with a fluorometer at 520 nm (Tecan Infinite M200, Tecan).

Western blot analyses

For the analysis of tight junction proteins, distal colon specimens were mechanically disassociated in an ice-cold membrane lysis buffer using a FastPrep-24 homogenizer (MP Biomedical). After the first centrifugation (1000 g, 5 min, 4 °C), supernatants were

once more centrifuged (42,000 g, 30 min, 4 °C) for membrane protein collection. The protein fraction was resolved in total lysis buffer and incubated 5 min at 95 °C in Laemmli buffer (See **Supplemental Table 6** for details on buffers and reagents). Samples were loaded on a 5% stacking gel and then separated on a 12% SDS polyacrylamide gel and transferred onto a polyvinylidene fluoride (PVDF) membrane (Perkin Elmer). PVDF Membranes were blocked in blocking solution and incubated overnight with primary antibodies (**Supplemental Table 7**) at 4 °C on a shaker. Membranes were washed in 1X Tris-buffered saline with 0.1% Tween® 20 solution and incubated with the respective peroxidase-conjugated secondary antibodies (Dako). Finally, membranes were washed, incubated in SuperSignalWest Pico PLUS buffer (ThermoFisher) and detected using a Fusion FX7 chemiluminescence imager (Vilber Lourmat). Densitometric analysis was performed using AIDA software (Elysia), and all values were normalized to the β-actin signal of the respective blot.

Calcium influx measurements in mice splenocytes

Splenocytes were isolated from lipodystrophic mice or Wildtype littermates and stained with 2 µg/mL calcium-sensing dye Fluo-4 AM (ThermoFisher Scientific) for 30 min on ice followed by an anti-mouse antibody cocktail targeting surface markers for T cells (CD4, CD8) for 15 min on ice in the dark. Cells were then washed using PBS supplemented with 10% FBS before being resuspended in 0 mM Ca^{2+} Ringer's solution (see in **Supplemental Table 8**). Baseline intracellular Ca^{2+} (f_0) was then measured for 30 or 40 seconds. Cells were then stimulated with 1 µM thapsigargin (Merck), and after 300 seconds a final concentration of 2 mM Ca^{2+}-containing Ringer's solution was added. The sample was then acquired for another 120 seconds. The mean fluorescence intensity (MFI) of Fluo-4 (f) was normalized to the mean MFI detected during the 40-second baseline measurement (f_0), and the resulting f/f_0 ratio was plotted against a time (t) axis. Graphs were generated using GraphPad Prism v.9 software (GraphPad).

Plasma treatment for calcium measurement

Spleens were isolated from WT mice and processed for splenocytes as described above. Cell pellets were washed and resuspended in RPMI medium 1640 (1X) (Gibco),

supplemented with 10% penicillin-streptomycin (Thermofisher Scientific), 50 µM 2-mercaptoethanol (Gibco) and 10% fetal bovine serum (FBS; Sigma-Aldrich). For experimental conditions, cultures additionally contained 10% pooled plasma from either WT or lipodystrophic mice. Cells were incubated for 16 h at 37 °C with 5% CO_2, followed by staining for intracellular calcium as previously described.

Supplementary References

1. Chevrier S, Crowell HL, Zanotelli VRT, Engler S, Robinson MD, and Bodenmiller B. Compensation of Signal Spillover in Suspension and Imaging Mass Cytometry. *Cell Syst.* 2018;6(5):612–20 e5.
2. Nowicka M, Krieg, C., Crowell, H.L., Weber, L.M., Hartmann, F.J., Guglietta, S., Becher, B., Levesque, M.P., Robinson, MD. CyTOF workflow: differential discovery in high-throughput high-dimensional cytometry datasets. *F1000Research.* 2019.
3. Bottcher C, Fernandez-Zapata C, Schlickeiser S, Kunkel D, Schulz AR, Mei HE, et al. Multi-parameter immune profiling of peripheral blood mononuclear cells by multiplexed single-cell mass cytometry in patients with early multiple sclerosis. *Sci Rep.* 2019;9(1):19471.
4. Hecker J, Letizia M, Loescher BS, Siegmund B, and Weidinger C. Early Onset of TNFalpha-Driven Arthritis, Auto-inflammation, and Progressive Loss of Vision in a Patient with ALPK1 Mutation. *J Clin Immunol.* 2022;42(4):880–4.
5. Ziegler JF, Bottcher C, Letizia M, Yerinde C, Wu H, Freise I, et al. Leptin induces TNFalpha-dependent inflammation in acquired generalized lipodystrophy and combined Crohn's disease. *Nat Commun.* 2019;10(1):5629.
6. Li H, and Durbin R. Fast and accurate short read alignment with Burrows-Wheeler transform. *Bioinformatics.*2009;25(14):1754–60.
7. Chan SSM, Chen Y, Casey K, Olen O, Ludvigsson JF, Carbonnel F, et al. Obesity is Associated With Increased Risk of Crohn's disease, but not Ulcerative Colitis: A Pooled Analysis of Five Prospective Cohort Studies. *Clin Gastroenterol Hepatol.* 2022;20(5):1048–58.
8. Mortazavi A, Williams BA, McCue K, Schaeffer L, and Wold B. Mapping and quantifying mammalian transcriptomes by RNA-Seq. *Nat Methods.* 2008;5(7):621–8.
9. Raudvere U, Kolberg L, Kuzmin I, Arak T, Adler P, Peterson H, et al. g:Profiler: a web server for functional enrichment analysis and conversions of gene lists (2019 update). *Nucleic Acids Res.* 2019;47(W1):W191–W8.
10. Weiss F, Czichos C, Knobe L, Voges L, Bojarski C, Michel G, et al. MarvelD3 Is Upregulated in Ulcerative Colitis and Has Attenuating Effects during Colitis Indirectly Stabilizing the Intestinal Barrier. *Cells.* 2022;11(9).
11. Srinivasan B, Kolli AR, Esch MB, Abaci HE, Shuler ML, and Hickman JJ. TEER measurement techniques for in vitro barrier model systems. *J Lab Autom.* 2015;20(2):107–26.

Supplemental Table 1. Clinical and demographic characteristics of cohorts included in the different experimental methods

Experiment (total number)	PMBC scRNAseq (n= 8)			LPMC scRNAseq (n= 4)		CyTOF (n=11)		
Cohorts´ characteristics	Crohn´s disease	AGLCD	Healthy	Crohn´s disease	AGLCD	Crohn´s disease	AGLCD	Non-IBD
number (%)	3 (37.5%)	1 (12.5%)	4 (50.0%)	3 (75%)	1 (25%)	6 (54.55%)	1 (9.09%)	4 (36.36%)
Mean age at inclusion in years (range)	36.8 (25-46)	(29-33)	34.5 (30-39)	36.8 (25-46)	(29-33)	55 (39-71)	(29-33)	73.33 (69-77)
Males number (%)	1 (33.3%)	1 (100.00%)	4 (100.00%)	1 (33.3%)	1 (100.00%)	1 (16.67%)	1 (100.00%)	3 (75.00%)
BMI (range)	25.8 (24.2-28.08)	17	22.9 (21.1-24)	25.8 (24.2-28.08)	17	25.8 (23.8-28.1)	17	23.8 (23.6-24)
Percentage of current or prior aminosalicylates medication (5-ASA)	66.67%	100.00%	0.00%	66.67%	100.00%	33.33%	100.00%	0.00%
Percentage of current or prior corticosteroids medication (Prednisolon, Hydrocortisone)	100.00%	100.00%	0.00%	100.00%	100.00%	50.00%	100.00%	0.00%
Percentage of current or prior immunomodulators medication (Tac)	0.00%	100.00%	0.00%	0.00%	100.00%	0.00%	100.00%	0.00%
Percentage of current or prior biologic therapies (agent used)	100.00% (IFX, ADA, UST)	100.00% (IFX)	0.00%	100.00% (IFX, ADA, UST)	100.00% (IFX)	66.67% (ADA)	100.00% (IFX)	0.00%
Chemotherapy at inclusion	0.00%	0.00%	0.00%	0.00%	0.00%	0.00%	0.00%	25.00%
Mean leucocyte count per nl at inclusion (range)	7.62 (4.45-9.52)	---	---	7.62 (4.45-9.52)	---	5.69 (4.01-9.52)	---	7.11 (5.77-8.47)

Tacrolimus (Tac), infliximab (IFX), adalimumab (ADA), and ustekinumab (UST).

Supplemental Table 2. Cell culture and in vitro assays medium

**Complete Medium**	**Cat. No.**	**Company**
RPMI Medium 1640 (1X)	#11875093	Gibco
10% Fetal bovine serum (FBS)	---	Sigma-Aldrich
10% Penicillin-Streptomycin	#15140122	Thermofisher Scientific
**Activation Medium**	---	---
RPMI Medium 1640 (1X)	#11875093	Gibco
10% FBS	---	Sigma-Aldrich
10% Penicillin-Streptomycin	#15140122	Thermofisher Scientific
50 μM 2-Mercaptoethanol	#31350010	Gibco
20 ng/ml phorbol 12-myristate 13-acetate (PMA)	#P8139	Sigma-Aldrich
1 μg/ml ionomycin	#I0634	Sigma-Aldrich
**T cell expansion Medium**	---	---
RPMI Medium 1640 (1X)	#11875093	Gibco
10% FBS	---	Sigma-Aldrich
10% Penicillin-Streptomycin	#15140122	Thermofisher Scientific
50 μM 2-Mercaptoethanol	#31350010	Gibco
250 U/ml human recombinant IL-2	#200-02	PeproTech, Rocky Hills, New
1:100 or 1:500 T Cell TransAct™, human	#130-111-160	Miltenyi Biotec, NRW, Germany
**Human LPMC digestion Medium**	---	---
RPMI Medium 1640 (1X)	#11875093	Gibco
10% FBS	---	Sigma-Aldrich
10% Penicillin-Streptomycin	#15140122	Thermofisher Scientific
0.15 mg/ml collagenase A	#11088793001	Roche, Basel, Switzerland
**Murine LPMC digestion Medium**	---	---
RPMI Medium 1640 (1X)	#11875093	Gibco
10% FBS	---	Sigma-Aldrich
10% Penicillin-Streptomycin	#15140122	Thermofisher Scientific
200 U/ml collagenase D	#11088858001	Roche

Supplemental Table 3: Anti-human antibodies used in mass cytometry

Metal	Target	Clone	Cat. No./ Company	Dilution
89Y	CD45	HI30	#3089003B / Fluidigm	1:100
141Pr	CD45	HI30	#3141009B / Fluidigm	1:100
142Nd	CD19	HIB19	#3142001B / Fluidigm	1:100
143Nd	CD45RA	HI100	#3143006B / Fluidigm	1:100
144Nd	IL-4	MP4-25D2	#3144010B / Fluidigm	1:100
145Nd	CD4	RPA-T4	#3145001B / Fluidigm	1:50
146Nd	TNFα	Mab11	#3146010B / Fluidigm	1:100
147Sm	CD11c	Bu15	#3147008B / Fluidigm	1:200
148Nd	IgA	Polyclonal	#3148007B / Fluidigm	1:100
149Sm	CD25	2A3	#3149010B / Fluidigm	1:100
150 Nd	CD86	IT2.2	#3150020B / Fluidigm	1:100
151Eu	CD103	Ber-ACT8	#3151011B / Fluidigm	1:100
152Sm	CD95/Fas	DX2	#3152017B / Fluidigm	1:200
153Eu	IgM	MHM-88	#314502 / Biolegend	1:200
154Sm	CD3	UCTH1	#3154003B / Fluidigm	1:100
155Gd	CD56	B159	#3155008B / Fluidigm	1:100
156Gd	IL-6	MQ2-13AS	#3156011B / Fluidigm	1:100
158Gd	IFNγ	B27	#3158017 / Fluidigm	1:400
159Tb	CCR7	G043H7	#3159003C / Fluidigm	1:200
160Gd	CD27	O323	#302802 / Biolegend	1:200
161Dy	IL-10	JES3-9D7	#3161008B / Fluidigm	1:100
161Dy	IL-23p19	23dcdp	#3161010B / Fluidigm	1:100
162Dy	CD8	RPA-T8	#3162015B / Fluidigm	1:100
163Dy	CD33	WM53	#3163023B / Fluidigm	1:100
163Dy	Granzyme B	QA16A02	#3173006B / Fluidigm	1:100
164Dy	CD45RO	UCHL1	#3164007B / Fluidigm	1:100
165Ho	CD40	5C3	#3165005B / Fluidigm	1:100
166Er	IL-2	MQ117H12	#3166002B / Fluidigm	1:100
167Er	CD38	HIT2	#3167001B / Fluidigm	1:200
168Er	CD40L	24-31	#3168006B / Fluidigm	1:100
169Tm	IL-13	JES105A2	#3169016 / Fluidigm	1:100
170Er	CD137	4B4-1	#3209015B / Biolegend	1:100
171Yb	CD178/FasL	NOK-1	#306402 / Biolegend	1:100
172Yb	IL-17	BL168	#3172020B / Fluidigm	1:100
173Yb	HLA-DR	L243	#3173005B / Fluidigm	1:200
174Yb	PD-1	EH12.2H7	#3174020B / Fluidigm	1:200
175Lu	CD14	M5E2	#3175015B / Fluidigm	1:50
175Lu	Perforin	B-D48	#3175004B / Fluidigm	1:100
176Yb	IL-7R	A019D5	#3176004B / Fluidigm	1:50
209Bi	CD11b	ICRF44	#3209003B / Fluidigm	1:100

Supplemental Table 4. Anti-human antibodies flow cytometry

Fluorochrome	Target	Clone	Cat. No. / Company	Dilution
FITC	CD14	61D3	#11-0149-42 / eBioscience	1:50
FITC	CD19	HIB19	#11-0199-42 / eBioscience	1:100
Pe-Cy7	CD16	3G8	#560918 / BD	1:200
APC	CD14	63D3	#367118 / Biolegend	1:50
APC	CD4	RPA-T4	#555349 / BD	1:100
APC	CD3	OKT3	#17-0037-42 / eBioscience	1:100
APC	CD56	TULY56	#17-0566-42 / eBioscience	1:200
APC-Cy7	CD19	HIB-19	#302218 / Biolegend	1:100
APC-Cy7	CD8	SK1	#344713 / Biolegend	1:100
Viogreen	CD3	REA613	#130-113-142 / Miltenyi Biotec	1:100
BV510	CD4	RPA-TA	#300546 / Biolegend	1:100

Supplemental Table 5. Anti-mouse antibodies for flow cytometry

Fluorochrome	Target	Clone	Cat. No. / Company	Dilution
FITC	CD4	GK1.5	#100406 / Biolegend	1:100
A488	Eomes	Dan11mag	#53-4875-80 / eBioscience	1:100
PE	Foxp3	FJK-16s	#12-5773-82 / eBioscience	1:100
PE	NK1.1	PK136	#12-5941-81 / eBioscience	1:200
PE	CD49a	REA493	#130-124-706 / Miltenyi Biotec	1:50
PerCP-Cy5.5	IL-17A	TC11-18H10.1	#506920 / Biolegend	1:200
PerCP-Cy5.5	CD3	145-2C11	#551163 / BD	1:200
PerCP-Cy5.5	CD27	LG.3A10	#124214 / BD	1:100
PerCP-Cy5.5	NK1.1	PK136	#108728 / Biolegend	1:50
Pe-Cy7	TNFα	MP6-XT22	#557644 / BD	1:200
Pe-Cy7	F4/80	BM8	#123118 / Biolegend	1:300
Pe-Cy7	CD49b	DX-5	#12-5971-82 / eBioscience	1:50
APC	IFNγ	XMG1.2	#554413 / BD	1:200
APC	T-bet	4B10	#644814 / Biolegend	1:500
APC-Cy7	CD4	GK1.5	# 100414 / Biolegend	1:500
APC-Cy7	CD45	30-F11	#103116 / Biolegend	1:200
APC-Cy7	CD11b	M1/70	#101226 / Biolegend	1:100
APC-Cy7	GR-1	RB6-8C5	#108434 / BD	1:200
APC-Cy7	CD19	6D5	#115530 / Biolegend	1:50
APC-Cy7	F4/80	BM8	#123118 / Biolegend	1:200
APC-Cy7	CD3e	145-2C11	#557596 / BD	1:50
APC-Cy7	FcERIa	MAR-1	#134326 / Biolegend	1:100
eFluor450	CD8a	53-6.7	#48-0081-82 / eBioscience	1:200
BV421	GR-1	RB6-8C5	#108434 / Biolegend	1:200
BV421	CD11b	M1/70	#101236 / Biolegend	1:100
V500	CD45	30-F11	#561487 / BD	1:200
Zombie Violet	Viability Dye		#423114 / Biolegend	1:1000
Zombie Aqua	Viability Dye		#423102 / Biolegend	1:1000
eFluor780	Viability Dye		#65-0865-14 / eBioscience	1:1000

Supplemental Table 6. Buffers used in western blotting

Tissue lysis buffer	Catalog number (Cat. No.)	Company
1 M Tris-HCl pH 7.4	#9090.3	Carl Roth, Karlsruhe, Germany
1 M $MgCl_2$	#5833	Merck, Darmstadt, Germany
0.5 M ethylenediaminetetraacetic acid (EDTA)	#E6511	Sigma-Aldrich, St. Louis, Missouri
0.5 M ethylene glycol-bis (β-aminoethyl ether)-N,N,N′,N′-tetraacetic acid (EGTA)	#324626	Sigma-Aldrich
Protease inhibitor cocktail	#P8340	Sigma-Aldrich
**RIPA buffer cocktail**	---	---
1X RIPA Buffer	#R0278	Merck
14X Protease inhibitor cocktail I	#20-201	Merck
10X Phosphatase inhibitor cocktail	#P5726	Merck
200mM NaVO4	#S6508	Merck
200mM NaF	#106449	Merck
**8% Sodium dodecyl sulfate (SDS) running gel**	---	---
40% acrylamide mix	#1610140	Biorad
5 mM Tris base pH 8.8	#4855.2	Carl Roth
10% SDS	#2326.2	Carl Roth
10% ammonium persulfate (APS)	#7727-54-0	Sigma-Aldrich
1% tetramethylethylenediamine (TEMED)	#161-0800	Biorad
**5% Stacking gel**	---	---
40% acrylamide mix	#1610140	Biorad
1.0 M Tris base pH 6.8	#4855.2	Carl Roth
10% SDS	#2326.2	Carl Roth
10% APS	#7727-54-0	Sigma-Aldrich
1% TEMED	#161-0800	Biorad
**Laemmli buffer (6X)**	---	---
60 mM Tris-HCl pH 6.8	#9090.3	Carl Roth
12% SDS	#2326.2	Carl Roth
47% glycerol	#3783.1	Carl Roth
0.06% bromophenol blue	#8122	Merck
12.5% β-mercaptoethanol	#60-24-2	Sigma-Aldrich
**Electrophoresis buffer (10X)**	---	---
144 g glycine	#3908.3	Carl Roth
30 g Tris base	#4855.5	Carl Roth
10 g SDS	#2326.2	Carl Roth
1 L distilled water	---	---
**Transfer buffer (10X)**	---	---
30.3 g Tris base	#4855.5	Carl Roth
144 g glycine	#3908.3	Carl Roth
1 L distilled water	---	---
**Transfer buffer (1X)**	---	---
100 ml transfer buffer (10X)	---	---
200 ml 100% methanol	#32213	Merck
700 ml distilled water	---	---
**Tris-Buffered Saline (TBS) pH 7.6 (10X)**	---	---
24 g Tris base	#4855.2	Carl Roth
88 g NaCl	#9265.2	Carl Roth

1 L distilled water	---	---
**Tris-Buffered Saline-Tween20 (TBS-T) (1X)**	---	---
100 ml TBS pH 7.6 (10X)	---	---
1 ml Tween	#9127.1	Carl Roth
900 ml distilled water	---	---
**5% Blocking buffer**	---	---
2.5 g bovine serum albumin (BSA) (Fraction V)	#9048-46-8	Sigma-Aldrich
50 ml TBS-T (1X)	---	---
2.5 mM L-glutamine	#56-85-9	Sigma-Aldrich

Supplemental Table 7. Antibodies used for western blot

Target	Clone	Cat. No. / Company	Dilution
CLDN1	Polyclonal	#19000 / Invitrogen	1:1000
CLDN2	MH44	#16100 / Invitrogen	1:1000
CLDN3	Polyclonal	#41700 / Invitrogen	1:1000
CLDN4	3E2C1	#29400 / Invitrogen	1:1000
CLDN7	Polyclonal	#49100 / Invitrogen	1:1000
OCCL	Polyclonal	#711500 / Invitrogen	1:1000
TRIC	54H19L38	#700191 / Invitrogen	1:1000
MD3	Polyclonal	#5567-1-AP/ Proteintech, Rosemont, IL	1:1000
ZO-1	ZO1-1A12	#33-9100 / Invitrogen	1:1000
LSR	Polyclonal	#HPA007270 / Atlas antibodies	1:1000
ILDR1	Polyclonal	#bs-11013R / Thermofisher	1:1000
ACTB	AC-15	#A5441 / Sigma-Aldrich	1:10,000

Supplemental Table 8. Buffers used for Ca^{2+} influx

Ringer Solution	Cat. No.	Company
155 mM NaCl	#9265.2	Merck
4.5 mM KCl	#7447-40-7	Merck
3 mM $MgCl_2$	#5833	Merck
10 mM D-glucose	#X997.2	Merck
5 mM Na-HEPES	#75277-39-3	Merck
± 4 mM $CaCl_2$	#2.382	Merck

Supplemental Table 9. Top 10 expanded clonotypes per sample in the AGLCD patient

Sample	Rank in sample	CTstrict	CTaa	Number of cells	Clonal proportion
LPMC_AGLCD_T1	1	TRAV8-1.TRAJ13.TRAC;TGTGCCGTGACCGCCGGGGGGGCTTCTGGGGGTTACCAGAAAGTTACCTTT_TRBV6-5.TRBD1.TRBJ1-2.TRBC1;TGTGCCAGCAGTTACTTCCCGCCCGGGACAGGGTTCTATGGCTACACCTTC	CAVTAGGASGGYQKVTF_CASSYFPPGTGFYGYTF	526	0.058353672
LPMC_AGLCD_T1	2	TRAV1-2.TRAJ41.TRAC;TGTGCTGTGAGAGAGGATTCCGGGTATGCACTCAACTTC_TRBV27..TRBJ2-4.TRBC2;TGTGCCAGCAGTCTCACCGTCAAAAACATTCAGTACTTC	CAVREDSGYALNF_CASSLTVKNIQYF	153	0.016973597
LPMC_AGLCD_T1	3	TRAV12-3.TRAJ15.TRAC;TGTGCAATGAGCGCGAAAGAGGCAGGAACTGCTCTGATCTTT_TRBV6-5..TRBJ1-6.TRBC1;TGTGCCAGCAGCCTTACCGACTCGGCAGGTTCACCCCTCCACTTT	CAMSAKEAGTALIF_CASSLTDSAGSPLHF	120	0.013312625
LPMC_AGLCD_T1	4	TRAV12-2.TRAJ4.TRAC;TGTGCCGTGATCCTGTTTTCTGGTGGCTACAATAAGCTGATTTTT_TRBV20-1..TRBJ2-3.TRBC2;TGCAGTGCTAGAGATCGACCTGGACGGGTCCTTTACACAGATACGCAGTATTTT	CAVILFSGGYNKLIF_CSARDRPGRVLYTDTQYF	93	0.010317284
LPMC_AGLCD_T1	5	TRAV26-2.TRAJ37.TRAC;TGCATCCTGACCCCCTCTAGCAACACAGGCAAACTAATCTTT_TRBV9..TRBJ2-7.TRBC2;TGTGCCAGCAGCGGATCTAGCGGGCGAGGACCCGAGCAGTACTTC	CILTPSSNTGKLIF_CASSGSSGRGPEQYF	80	0.008875083
LPMC_AGLCD_T1	6	TRAV29/DV5.TRAJ49.TRAC;TGTGCAGCAAGCGGGGCAATTTCCGGTAACCAGTTCTATTTT_TRBV9..TRBJ1-2.TRBC1;TGTGCCAGCAGCCCAGGGTCCCTGGGGAACTATGGCTACACCTTC	CAASGAISGNQFYF_CASSPGSLGNYGYTF	70	0.007765698
LPMC_AGLCD_T1	7	TRAV12-2.TRAJ8.TRAC;TGTGCCGCCAGAGAAATGAACACAGGCTTTCAGAAACTTGTATTT_TRBV19..TRBJ1-1.TRBC1;TGTGCCAGTAGTATAGACATGAACACTGAAGCTTTCTTT	CAAREMNTGFQKLVF_CASSIDMNTEAFF	55	0.00610162
LPMC_AGLCD_T1	8	TRAV14/DV4.TRAJ9.TRAC;TGTGCAATGAGAGAGGGCTCAGTAGGCTTCAAAACTATCTTT_TRBV14.TRBD1.TRBJ1-1.TRBC1;TGTGCCAGCAGCCGGAGGGACAGAGTGAACACTGAAGCTTTCTTT	CAMREGSVGFKTIF_CASSRRDRVNTEAFF	52	0.005768804
LPMC_AGLCD_T1	9	TRAV4.TRAJ17.TRAC;TGCCTCGTGGGTGAGGGGGCTGCAGGCAACAAGCTAACTTTT_TRBV9..TRBJ2-5.TRBC2;TGTGCCAGCAGCGCCATAGCGGGGGGGCCGGGGGAGACCCAGTACTTC	CLVGEGAAGNKLTF_CASSAIAGGPGETQYF	52	0.005768804
LPMC_AGLCD_T1	10	TRAV35.TRAJ49.TRAC;TGTGCTGGCCTTTCGAACACCGGTAACCAGTTCTATTTT_TRBV5-1..TRBJ1-2.TRBC1;TGCGCCAGCAGCTTGGAAGGGTATGGCTACACCTTC	CAGLSNTGNQFYF_CASSLEGYGYTF	51	0.005657866
PBMC_AGLCD_T1	1	TRAV30.TRAJ49.TRAC;TGTGGCACAGACACCGGTAACCAGTTCTATTTT_TRBV5-6..TRBJ1-5.TRBC1;TGTGCCAGCAGCTTGTGGGGACCCCGGAGCAATCAGCCCCAGCATTTT	CGTDTGNQFYF_CASSLWGPRSNQPQHF	56	0.014238495
PBMC_AGLCD_T1	2	TRAV26-1.TRAJ49.TRAC;TGCATCGTCAGAGGAGGCACCGGTAACCAGTTCTATTTT_TRBV4-2..TRBJ1-6.TRBC1;TGTGCCAGCAGCCAGAGGAACCTGGACGGATATAATTCACCCCTCCACTTT	CIVRGGTGNQFYF_CASSQRNLDGYNSPLHF	53	0.013475718
PBMC_AGLCD_T1	3	TRAV8-2.TRAJ4.TRAC;TGTGTTGTGAGTGTATTTTCTGGTGGCTACAATAAGCTGATTTTT_TRBV5-6..TRBJ2-6.TRBC2;TGTGCCAGCGCCGCCGGCGGGAACAGCCACAATTATCCCTCTGGGGCCAACGTCCTGACTTTC	CVVSVFSGGYNKLIF_CASAAGGNSHNYPSGANVLTF	50	0.012712942
PBMC_AGLCD_T1	4	TRAV35.TRAJ45.TRAC;TGTGCTGGTCTCGCAGATTCAGGAGGAGGTGCTGACGGACTCACCTTT_TRBV5-5..TRBJ2-6.TRBC2;TGTGCCAGCAGCTTTTACCCCGGCCGCTCTGGGGCCAACGTCCTGACTTTC	CAGLADSGGGADGLTF_CASSFYPGRSGANVLTF	39	0.009916095
PBMC_AGLCD_T1	5	TRAV12-3.TRAJ24.TRAC;TGTGCAATGAGGTTAACTGACAGCTGGGGGAAATTGCAGTTT_TRBV7-6..TRBJ1-1.TRBC1;TGTGCCAGCAGCTTAGAGGTTGGTTATGAAGCTTTCTTT	CAMRLTDSWGKLQF_CASSLEVGYEAFF	33	0.008390542
PBMC_AGLCD_T1	6	TRAV35.TRAJ49.TRAC;TGTGCTGGCCTTTCGAACACCGGTAACCAGTTCTATTTT_TRBV5-1..TRBJ1-2.TRBC1;TGCGCCAGCAGCTTGGAAGGGTATGGCTACACCTTC	CAGLSNTGNQFYF_CASSLEGYGYTF	31	0.007882024
PBMC_AGLCD_T1	7	TRAV12-2.TRAJ4.TRAC;TGTGCCGTGATCCTGTTTTCTGGTGGCTACAATAAGCTGATTTTT_TRBV20-1..TRBJ2-3.TRBC2;TGCAGTGCTAGAGATCGACCTGGACGGGTCCTTTACACAGATACGCAGTATTTT	CAVILFSGGYNKLIF_CSARDRPGRVLYTDTQYF	29	0.007373506
PBMC_AGLCD_T1	8	TRAV12-1.TRAJ41.TRAC;TGTGTGGTGAACATCTCTTATTCCGGGTATGCACTCAACTTC_TRBV5-6..TRBJ2-6.TRBC2;TGTGCCAGCGCCGCCGGCGGGAACAGCCACAATTATCCCTCTGGGGCCAACGTCCTGACTTTC	CVVNISYSGYALNF_CASAAGGNSHNYPSGANVLTF	28	0.007119247
PBMC_AGLCD_T1	9	TRAV16.TRAJ13.TRAC;TGTGCTCTTGTGTCTGGGGGTTACCAGAAAGTTACCTTT_TRBV7-6..TRBJ2-2.TRBC2;TGTGCCAGCAGCCTGACAGGTGCCGACACCGGGGAGCTGTTTTTT	CALVSGGYQKVTF_CASSLTGADTGELFF	28	0.007119247
PBMC_AGLCD_T1	10	TRAV35.TRAJ28.TRAC;TGTGCTGGGCAGCCGCCGGCGTCCGGGGCTGGGAGTTACCAACTCACTTTC_TRBV20-1..TRBJ1-6.TRBC1;TGCAGTGCTAGAGGTGGGCTTAATTCACCCCTCCACTTT	CAGQPPASGAGSYQLTF_CSARGGLNSPLHF	25	0.006356471
PBMC_AGLCD_T2	1	TRAV8-2.TRAJ4.TRAC;TGTGTTGTGAGTGTATTTTCTGGTGGCTACAATAAGCTGATTTTT_TRBV5-6..TRBJ2-6.TRBC2;TGTGCCAGCGCCGCCGGCGGGAACAGCCACAATTATCCCTCTGGGGCCAACGTCCTGACTTTC	CVVSVFSGGYNKLIF_CASAAGGNSHNYPSGANVLTF	111	0.014302281
PBMC_AGLCD_T2	2	TRAV30.TRAJ49.TRAC;TGTGGCACAGACACCGGTAACCAGTTCTATTTT_TRBV5-6..TRBJ1-5.TRBC1;TGTGCCAGCAGCTTGTGGGGACCCCGGAGCAATCAGCCCCAGCATTTT	CGTDTGNQFYF_CASSLWGPRSNQPQHF	105	0.013529184
PBMC_AGLCD_T2	3	TRAV35.TRAJ49.TRAC;TGTGCTGGCCTTTCGAACACCGGTAACCAGTTCTATTTT_TRBV5-1..TRBJ1-2.TRBC1;TGCGCCAGCAGCTTGGAAGGGTATGGCTACACCTTC	CAGLSNTGNQFYF_CASSLEGYGYTF	74	0.009534854
PBMC_AGLCD_T2	4	TRAV35.TRAJ28.TRAC;TGTGCTGGGCAGCCGCCGGCGTCCGGGGCTGGGAGTTACCAACTCACTTTC_TRBV20-1..TRBJ1-6.TRBC1;TGCAGTGCTAGAGGTGGGCTTAATTCACCCCTCCACTTT	CAGQPPASGAGSYQLTF_CSARGGLNSPLHF	66	0.008504059
PBMC_AGLCD_T2	5	TRAV12-1.TRAJ41.TRAC;TGTGTGGTGAACATCTCTTATTCCGGGTATGCACTCAACTTC_TRBV5-6..TRBJ2-6.TRBC2;TGTGCCAGCGCCGCCGGCGGGAACAGCCACAATTATCCCTCTGGGGCCAACGTCCTGACTTTC	CVVNISYSGYALNF_CASAAGGNSHNYPSGANVLTF	60	0.007730963
PBMC_AGLCD_T2	6	TRAV26-1.TRAJ49.TRAC;TGCATCGTCAGAGGAGGCACCGGTAACCAGTTCTATTTT_TRBV4-2..TRBJ1-6.TRBC1;TGTGCCAGCAGCCAGAGGAACCTGGACGGATATAATTCACCCCTCCACTTT	CIVRGGTGNQFYF_CASSQRNLDGYNSPLHF	58	0.007473264
PBMC_AGLCD_T2	7	TRAV12-2.TRAJ4.TRAC;TGTGCCGTGATCCTGTTTTCTGGTGGCTACAATAAGCTGATTTTT_TRBV20-1..TRBJ2-3.TRBC2;TGCAGTGCTAGAGATCGACCTGGACGGGTCCTTTACACAGATACGCAGTATTTT	CAVILFSGGYNKLIF_CSARDRPGRVLYTDTQYF	55	0.007086716
PBMC_AGLCD_T2	8	TRAV5.TRAJ4.TRAC;TGTGCAGAGACCTTTTCTGGTGGCTACAATAAGCTGATTTTT_TRBV5-6.TRBD1.TRBJ1-2.TRBC1;TGTGCCAGCAGCTCTTGGGGACAGGGGTACGGCTACACCTTC	CAETFSGGYNKLIF_CASSSWGQGYGYTF	53	0.006829017
PBMC_AGLCD_T2	9	TRAV35.TRAJ45.TRAC;TGTGCTGGTCTCGCAGATTCAGGAGGAGGTGCTGACGGACTCACCTTT_TRBV5-5..TRBJ2-6.TRBC2;TGTGCCAGCAGCTTTTACCCCGGCCGCTCTGGGGCCAACGTCCTGACTTTC	CAGLADSGGGADGLTF_CASSFYPGRSGANVLTF	50	0.006442469
PBMC_AGLCD_T2	10	TRAV12-1.TRAJ4.TRAC;TGTGTGGTGACCCGCCCTTCTGGTGGCTACAATAAGCTGATTTTT_TRBV4-1..TRBJ2-1.TRBC2;TGCGCCAGCAGCCAAGAACTAGCGGGGGGCAAGATTCCCCTCAGCTCCTACAATGAGCAGTTCTTC	CVVTRPSGGYNKLIF_CASSQELAGGKIPLSSYNEQFF	34	0.004380879

Supplemental Figure 1

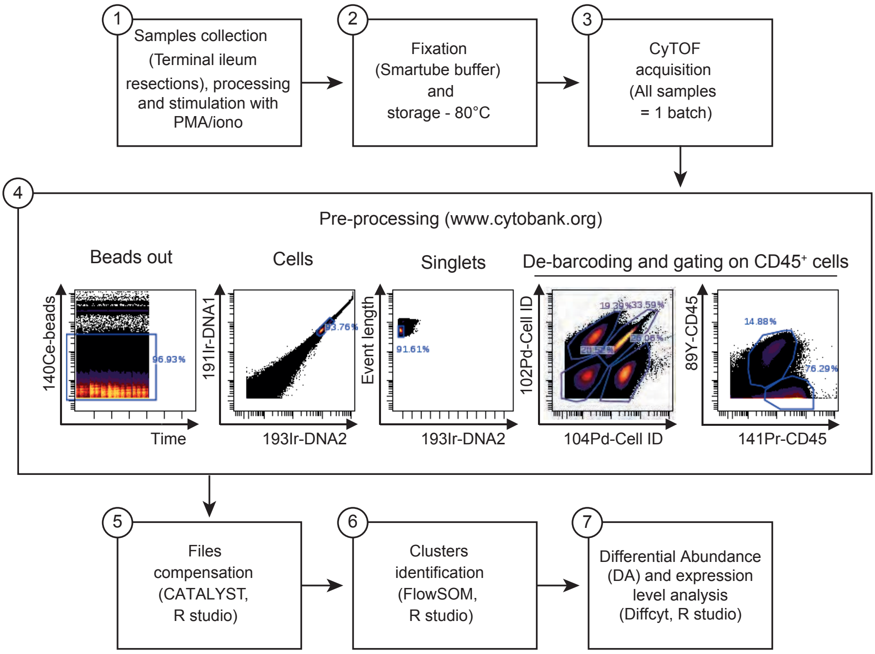

Supplemental Figure 1. Experimental setup to characterize gut-resident immune cells via mass cytometry. Lamina propria mononuclear cells (LPMCs) were isolated from terminal ileum samples obtained from 6 Crohn's disease (CD) patients, 4 non-IBD control patients and one AGLCD patient. Cells were stimulated with PMA/ionomycin for 4h before being stored at -80°C until acquisition. All the samples collected were acquired on a CyTOF2 mass cytometer in one batch. For data analysis, resulting flow cytometry standard (FCS) files were first normalized and then uploaded to Cytobank (www.cytobank.org) for gating of single, live cells and de-barcoding. Individual FCS files were compensated using the R package CATALYST and 17 clusters were identified using the R package FlowSOM. Differential abundance and expression level analyses were then done using the R package Diffyct.

Supplemental Figure 2

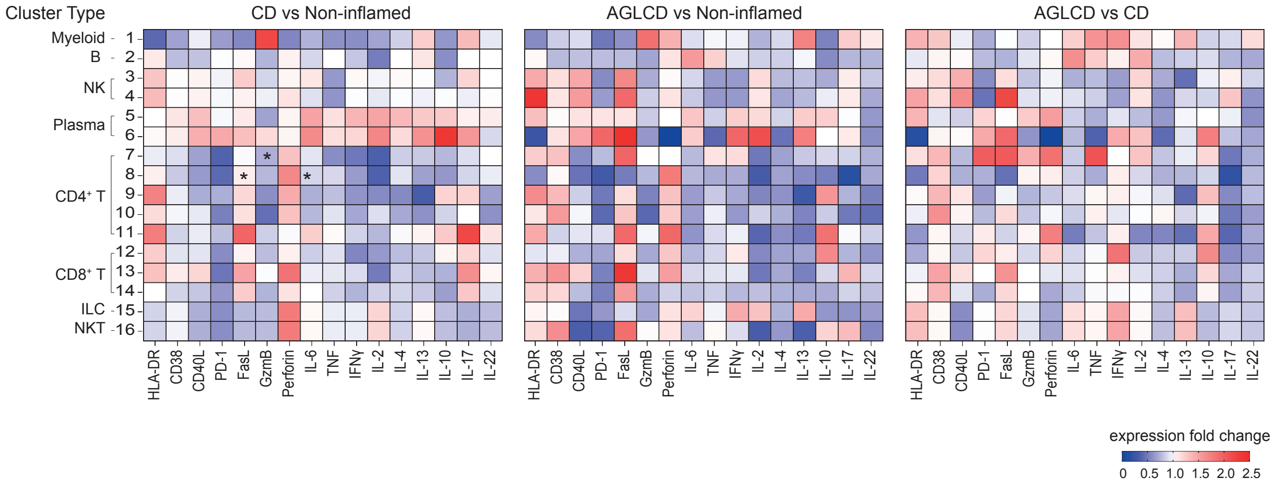

Supplemental Figure 2. Differences in expression levels of activation markers, cytokines and pore-forming cytolytic proteins between AGLCD, CD, and non-inflamed control cell clusters. LPMCs were isolated from AGLCD patient and controls (AGLCD: n=1, CD: n= 6, non-inflamed: n=4) and then activated for 4 h in vitro with PMA/ionomycin. Heatmaps show the median fold change of cytokines and activation marker expression in different immune cell populations. Significant differences were calculated using multiple t tests, *P < 0.05.

Supplemental Figure 3

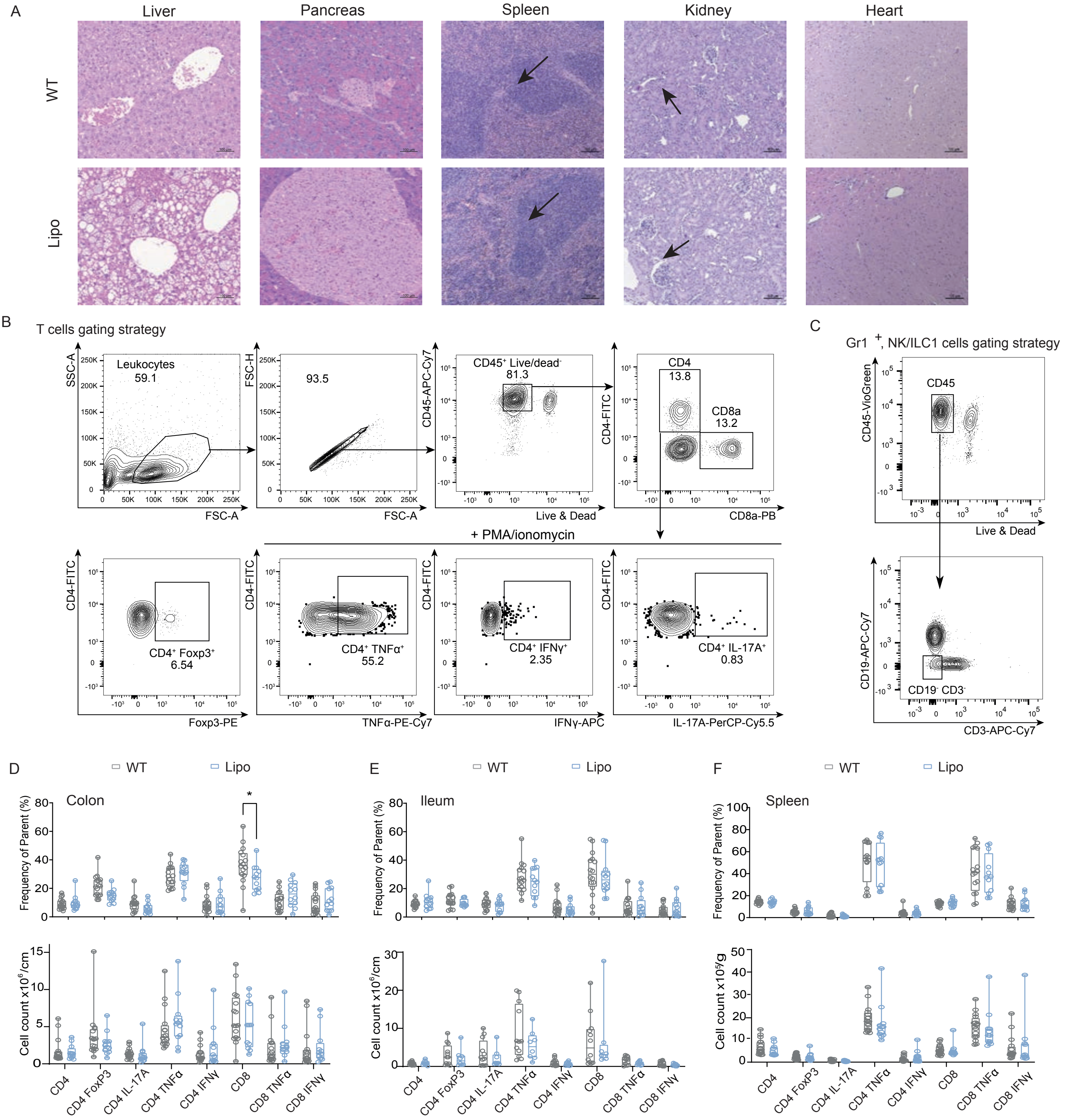

Supplemental Figure 3. Characterization of steady state lipodystrophic and wild-type mice using histology and flow cytometry (**A**) Representative histologic analyses of various organs obtained from wild-type (WT) and lipodystrophic (lipo) mice. Arrows point to the enlarged red pulp in the spleen and enlarged glomeruli in the kidney. (**B**) Immune cells isolated from spleen or intestinal tissues of WT and lipo mice were characterized using flow cytometry. Cells were gated for scattering (SSC-A/ FCS-A), then doublets (FSC-H/FSC-A) were excluded, and only live immune ($CD45^+$ Live&Dead$^-$) cells were selected for further evaluation. For cytokine staining of $CD4^+$ or $CD8^+$ T cells, cells were stimulated in vitro for 4 h with PMA/ionomycin. (**C**) For NK/ILC1 cell populations, only live immune ($CD45^+$ Live&Dead$^-$) cells that were also $CD19^-CD3^-$ were selected for further evaluation. Box-and-whisker plots showing the frequency (top panels) and absolute cell counts (bottom panels) of indicated immune cell subsets in the colon (**D**), ileum (**E**), and spleen (**F**). Data were pooled from n = 8-16 mice per group and analyzed by two-way ANOVA with Šídák's correction. Boxes range from the 25th to the 75th percentile. Whisker plots show the minimum (smallest) and maximum (largest) values. The line in the box indicates the median.

Supplemental Figure 4

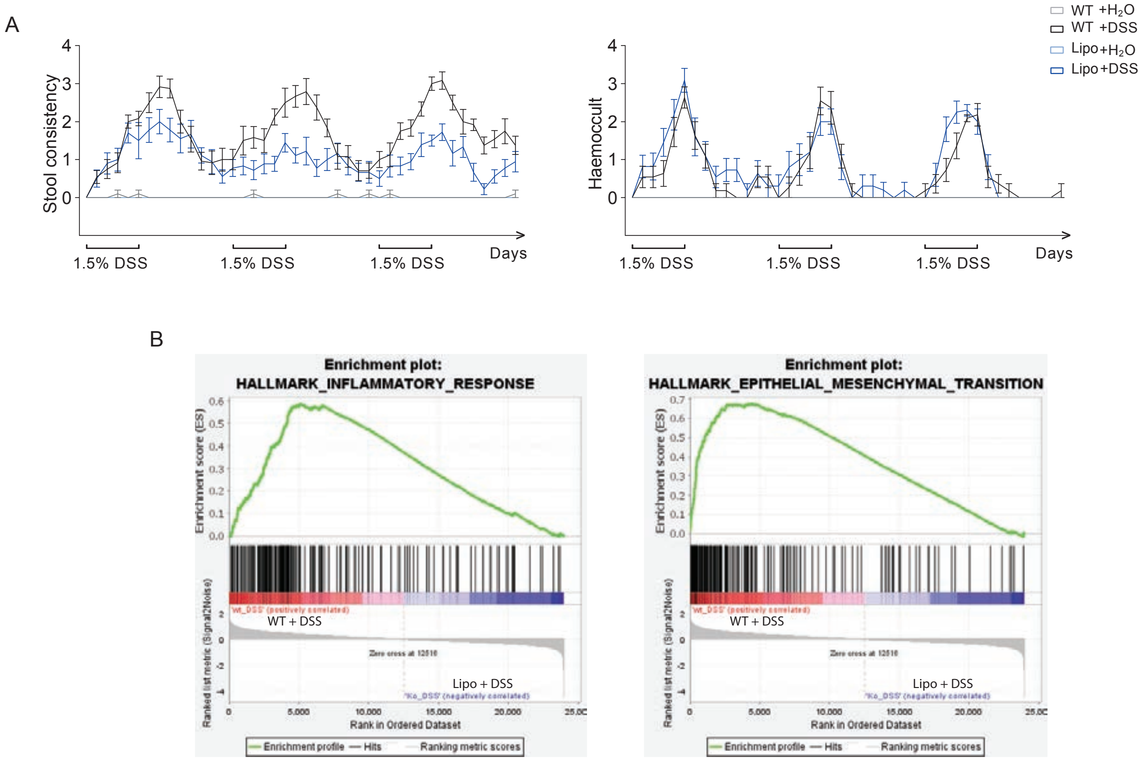

Supplemental Figure 4. Lipodystrophic mice have less severe colitis and altered immune responses compared to WT mice in the chronic DSS model. Mice received three cycles of 1.5% DSS and were monitored for (**A**) stool consistency and tested for hemoccult blood during the experiment. Data was pooled from two independent experiments. Lines are projections of mean values, and the error bars represent the standard error mean. (WT+ H2O: n=10; WT+ DSS: n=11; Lipo + H2O: n=6; Lipo + DSS: n=11). (**B**) Gene set enrichment analysis (GSEA) plot showing upregulation of genes involved in the inflammatory response and epithelial-mesenchymal transition gene sets in the WT DSS-treated mice compared to lipoatrophic mice DSS-treated mice (n = 5 per group).

Supplemental Figure 5

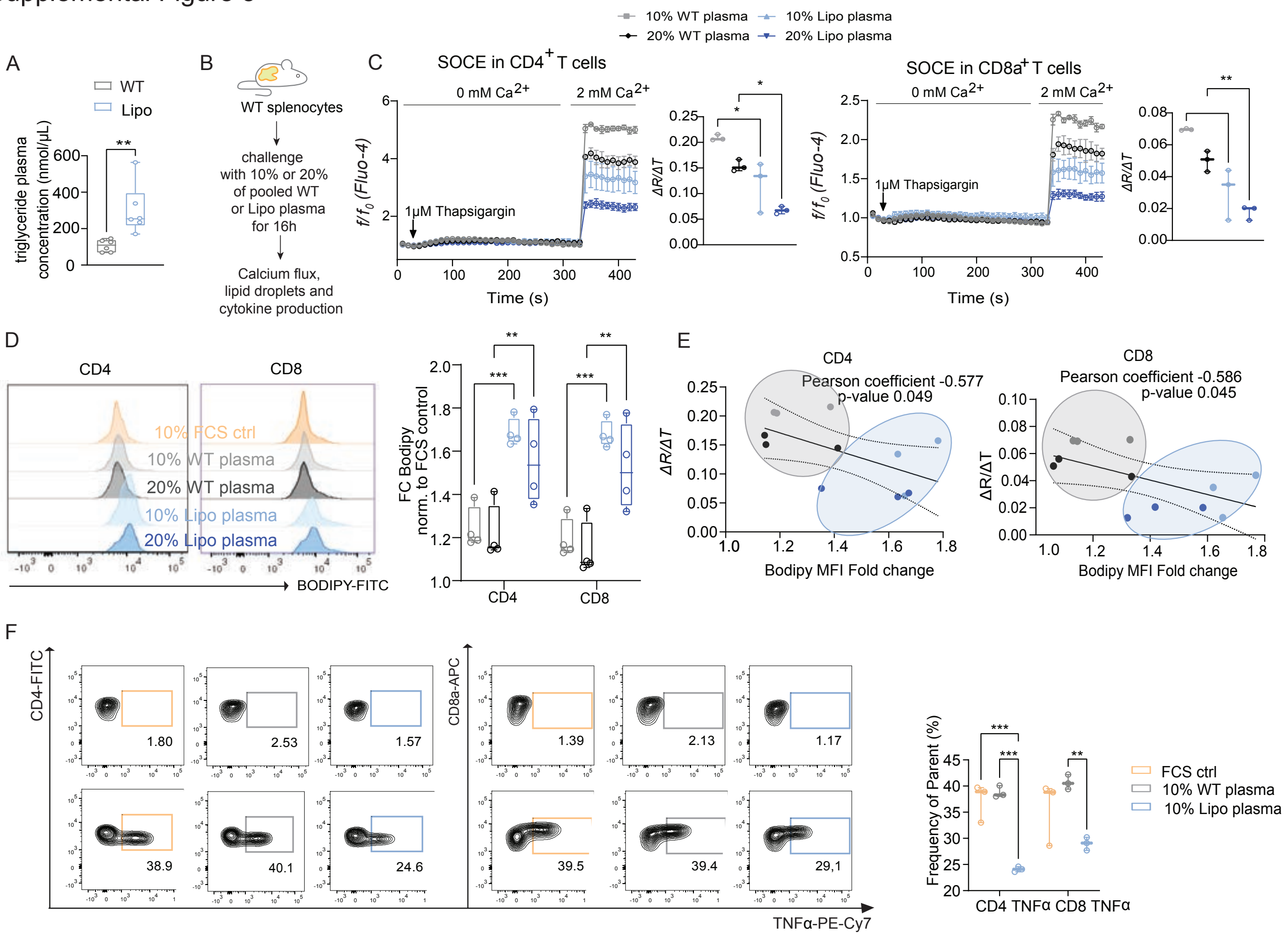

Supplemental Figure 5. Exposure of wild-type splenocytes to triglyceride-containing plasma from lipodystrophic mice impairs Ca^{2+} homeostasis. (**A**) Triglyceride levels in plasma of lipo mice compared with WT controls (6 per group), as assessed by triglyceride assay quantification kit. (**B**) Experimental setup (**C**) Representative calcium traces and bar graphs quantifying store-operated calcium entry (SOCE) influx rate in $CD4^+$ and $CD8^+$ T cells after exposure to plasma from WT or lipo mice. (**D**) Histograms showing lipid droplet accumulation in WT splenocytes after challenge with plasma from WT or lipo mice. (**E**) Correlation plots between lipid droplet accumulation and calcium flux rates in $CD4^+$ and $CD8^+$ T cells after incubation in WT or lipo plasma overnight. Spleens were collected from 3-4 mice and run in experimental triplicates. (**F**) TNFα production in T cells after 10% plasma exposure. Statistical test used for A and C was Mann-Whitney test and two-way ANOVA with Šídák's multiple comparisons test for D and F.

Supplemental Figure 6

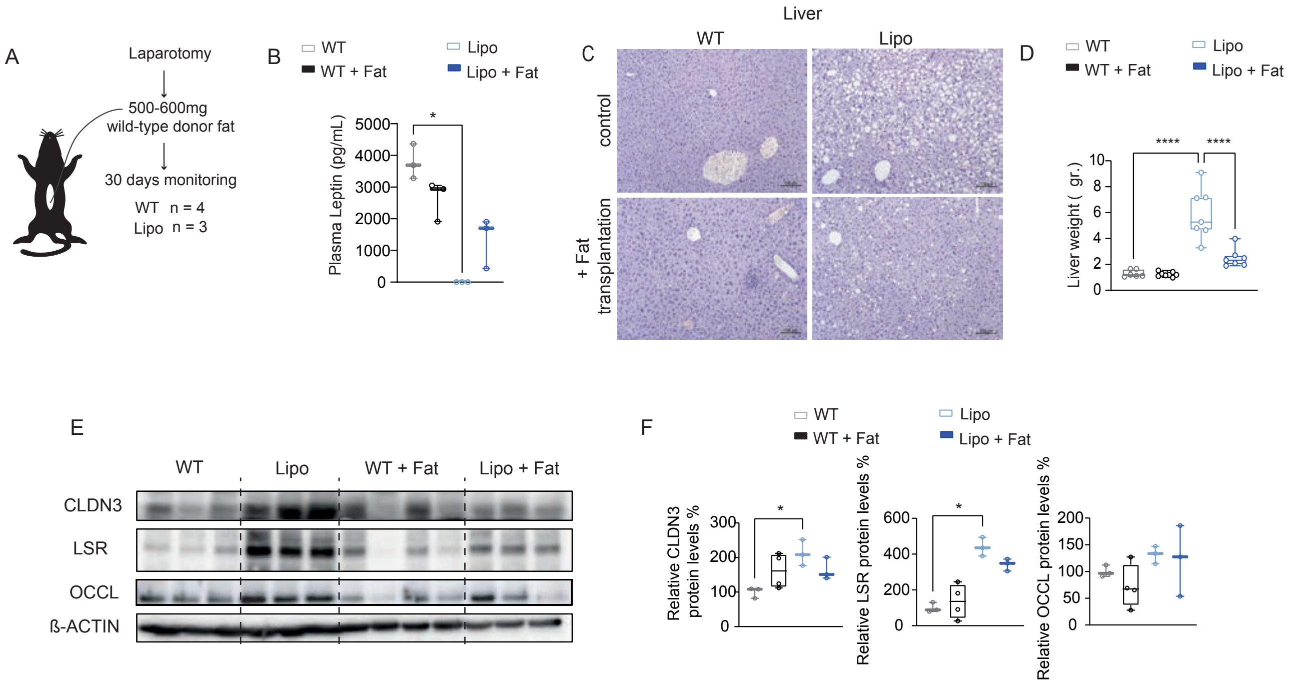

Supplemental Figure 6. Allogenic fat transplantation reverses steatohepatitis and alters tight junction protein expression in lipodystrophic mice. (**A**) Experimental design: lipodystrophic (Lipo) or wild-type (WT) mice were transplanted with 500-600 mg adipose tissue obtained from WT donor mice by performing mini-laparotomy. Mice were monitored for 30 days before organs were harvested for further experimentation. (**B**) Plasma levels of leptin. (**C**) Representative H&E staining of liver sections, (**D**) liver weight, (**E**) Western blot showing expression levels of TJs proteins isolated from the colon lamina propria of mice (WT: n = 3; WT + fat: n = 4; Lipo: n = 3; Lipo + fat: n = 3). (**F**) Whisker and box plots show relative expression of Claudin 3 (CLDN3), Lipoprotein receptor (LSR), Occludin (OCCL) proteins in lipoatrophic mice normalized to wild-type controls (WT= 100%). Boxes range from the 25th to the 75th percentile. Whisker plots show the minimum (smallest) and maximum (largest) values. The line in the box indicates the median (SEM). Statistics was calculated by one-way ANOVA test.

Supplemental Figure 7

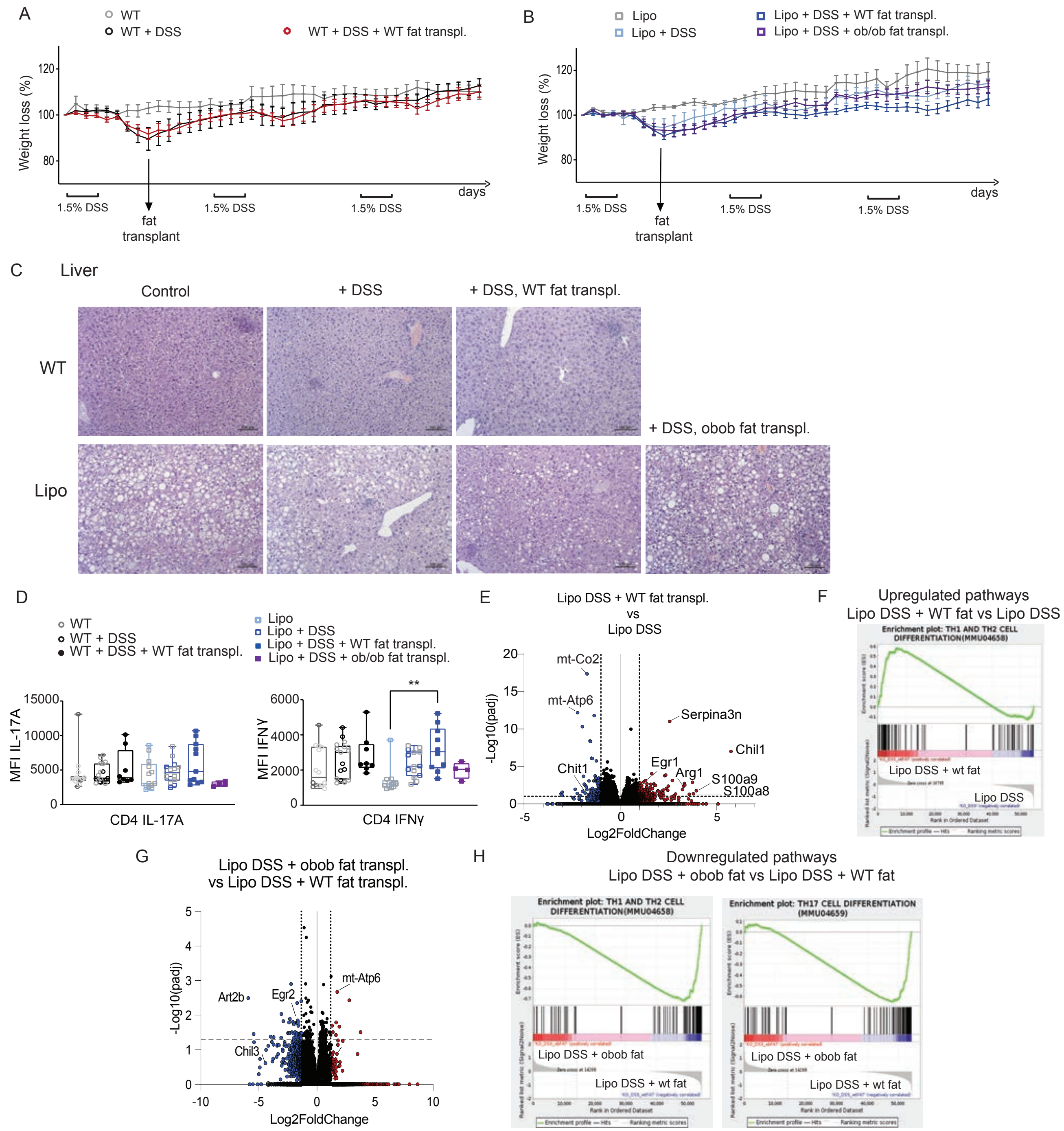

Supplemental Figure 7. Lipodystrophic mice that received allogenic wild-type (WT) fat show reduced colitis compared to lipodystrophic mice that received allogenic ob/ob fat in the chronic DSS model. Lipodystrophic (Lipo) or WT mice received 1 cycle of 1.5% DSS followed by transplantation with 500-600 mg adipose tissue obtained from WT or leptin-deficient ob/ob donor mice by performing mini-laparotomy. Seven days after transplantation, mice were subsequently challenged with 2 cycles of DSS treatment. Mice were monitored throughout the experiment and relative weight loss was recorded (**A**) for Lipo and (**B**) WT mice groups. Lines are projections of mean values. Data shown is pooled from 2 independent transplantation experiments (n=4-13). (**C**) Representative H&E staining of liver sections (**D**) Box-and-whisker plots summarizing MFI IFNγ- and IL-17A–producing CD4+ T cells normalized to WT mice (n=4-18; pooled from five experiments). Statistical differences were calculated using the Mann-Whitney U test. (**E**) Volcano plot showing differentially regulated genes between DSS-treated Lipo mice that received WT fat and DSS-treated Lipo mice (n=6 per group). (**F**) Gene set enrichment analysis (GSEA) plot showing upregulation of genes involved in Th1 and Th2 cell differentiation in DSS-treated lipodystrophic that received WT fat and DSS-treated Lipo mice. (**G**) Volcano plot showing differentially regulated genes in DSS-treated Lipo mice that received *ob/ob* fat compared to DSS-treated Lipo mice that received WT fat (n=4-6). (**H**) GSEA plot showing downregulation of genes involved in Th1, Th2 and Th17 cell differentiation in DSS-treated Lipo mice that received *ob/ob* fat and DSS-treated Lipo mice that received WT fat.

Supplemental Figure 8

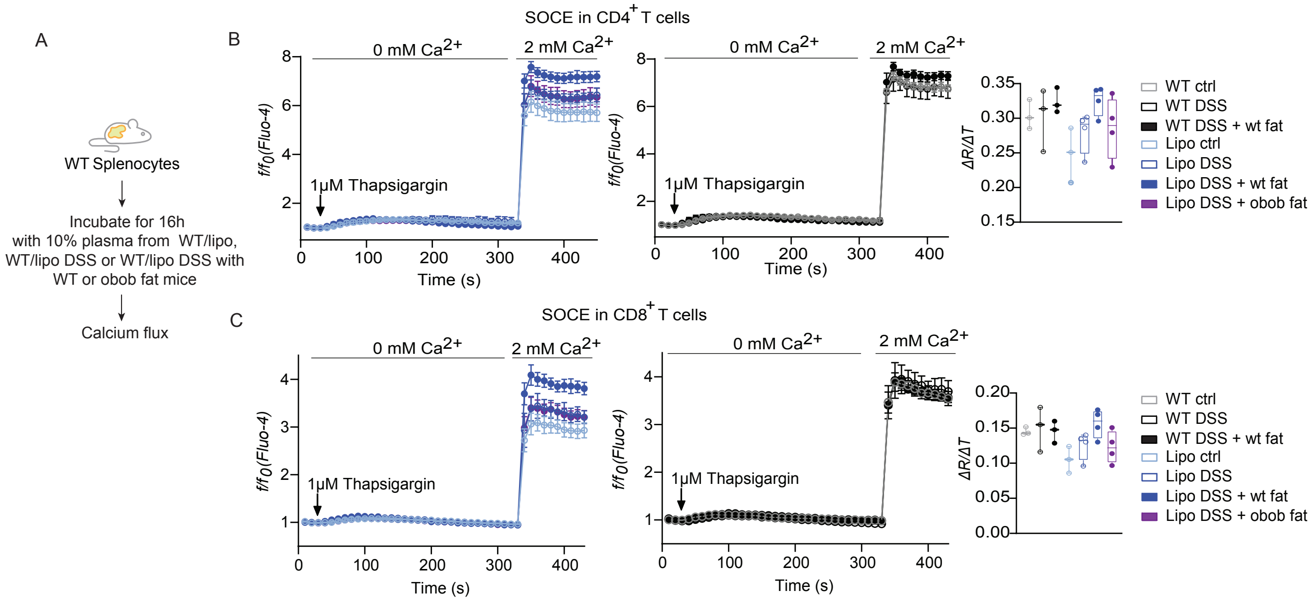

Supplemental Figure 8. Improved SOCE in T cells after challenge with plasma from fat-transplanted lipodystrophic mice. (A) Experimental setup of plasma-challenge experiments. Representative calcium traces and bar graphs quantifying store-operated calcium entry (SOCE) in wild-type (**B**) $CD4^{+}$ and (**C**) $CD8^{+}$ T cells after exposure to plasma derived from WT or lipo control mice, DSS-treated WT or lipo mice, DSS-treated WT or lipo mice transplanted with WT fat, or DSS-treated lipo mice transplanted with ob/ob fat (n=3-4)

Supplemental Figure 9

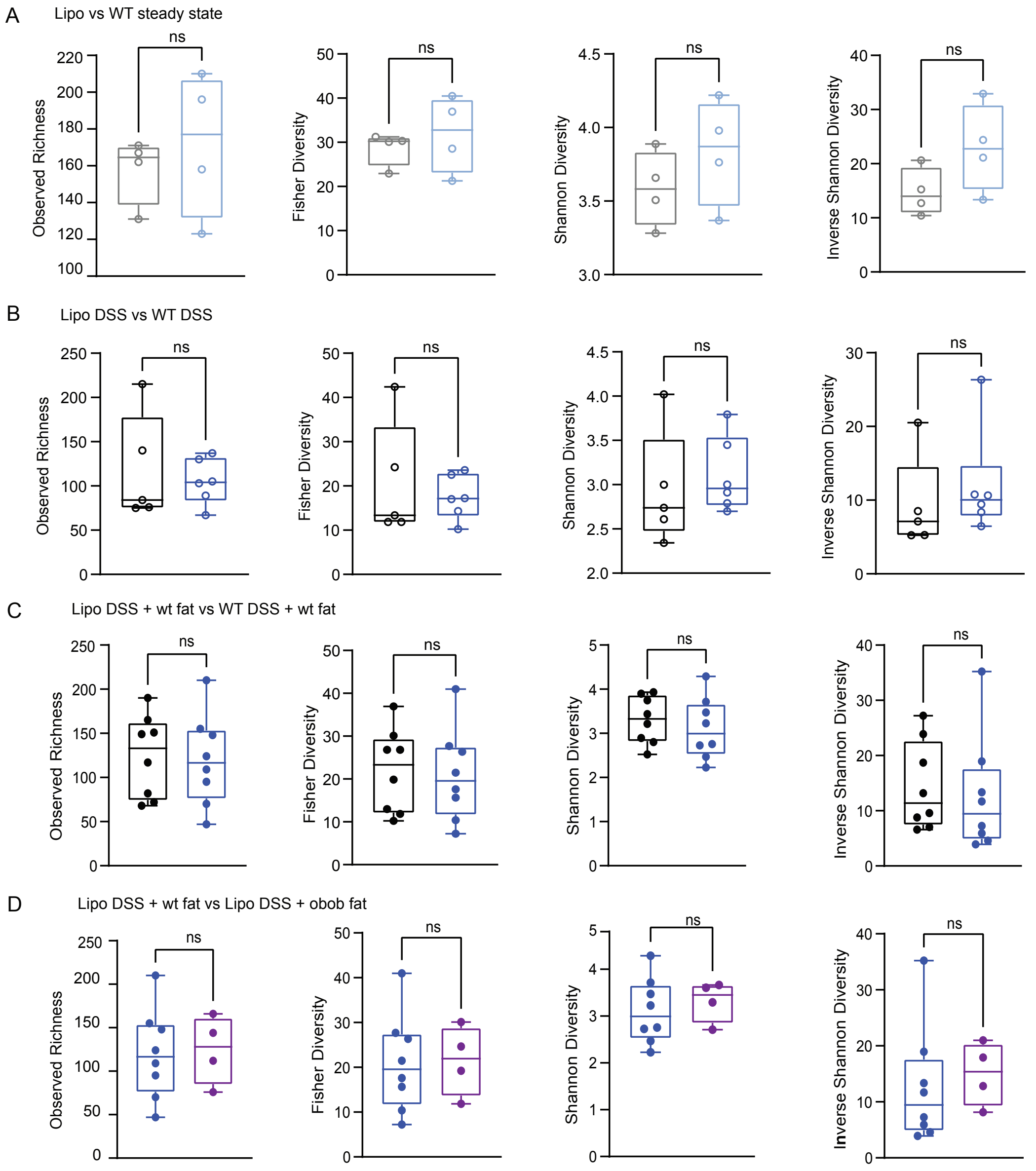

Supplemental Figure 9. The gut microbiota is not significantly altered in lipodystrophic mice compared to wt littermates. Alpha diversity was assessed using 16S rRNA gene sequencing and is presented as box-and-whisker plots for Observed Richness, Fisher Diversity, Shannon Diversity, and Inverse Shannon Diversity for the following comparisons (**A**) Lipo (n=4) vs WT (n=4) mice at steady state, (**B**) DSS-treated lipo mice (n=5) vs DSS-treated WT mice (n=4), (**C**) DSS-treated lipo mice + wt fat (n=8) vs DSS-treated WT mice + wt fat (n=8), and (**D**) DSS-treated lipo mice + wt fat (n=8) vs DSS-treated lipo mice + ob/ob fat (n=4). Each point represents an individual mouse. Statistical comparisons between groups were performed using Pairwise Wilcoxon Tests.
