# Supplementary material for: Characterization of intestinal immune responses in generalized human and murine lipodystrophy
Source: J Clin Invest. 2026 Mar 16;136(6):e192322. doi: 10.1172/JCI192322 (PMC12987653; doi:10.1172/JCI192322)

Western Blots

Merged images with ladder (Thermo Fisher, PageRuler Plus Prestained Protein Ladder 10 to 250 kDa, #26620),
not adjusted as during densitometric analysis and visualization in figures

Fig. 4I-J: Cldn1

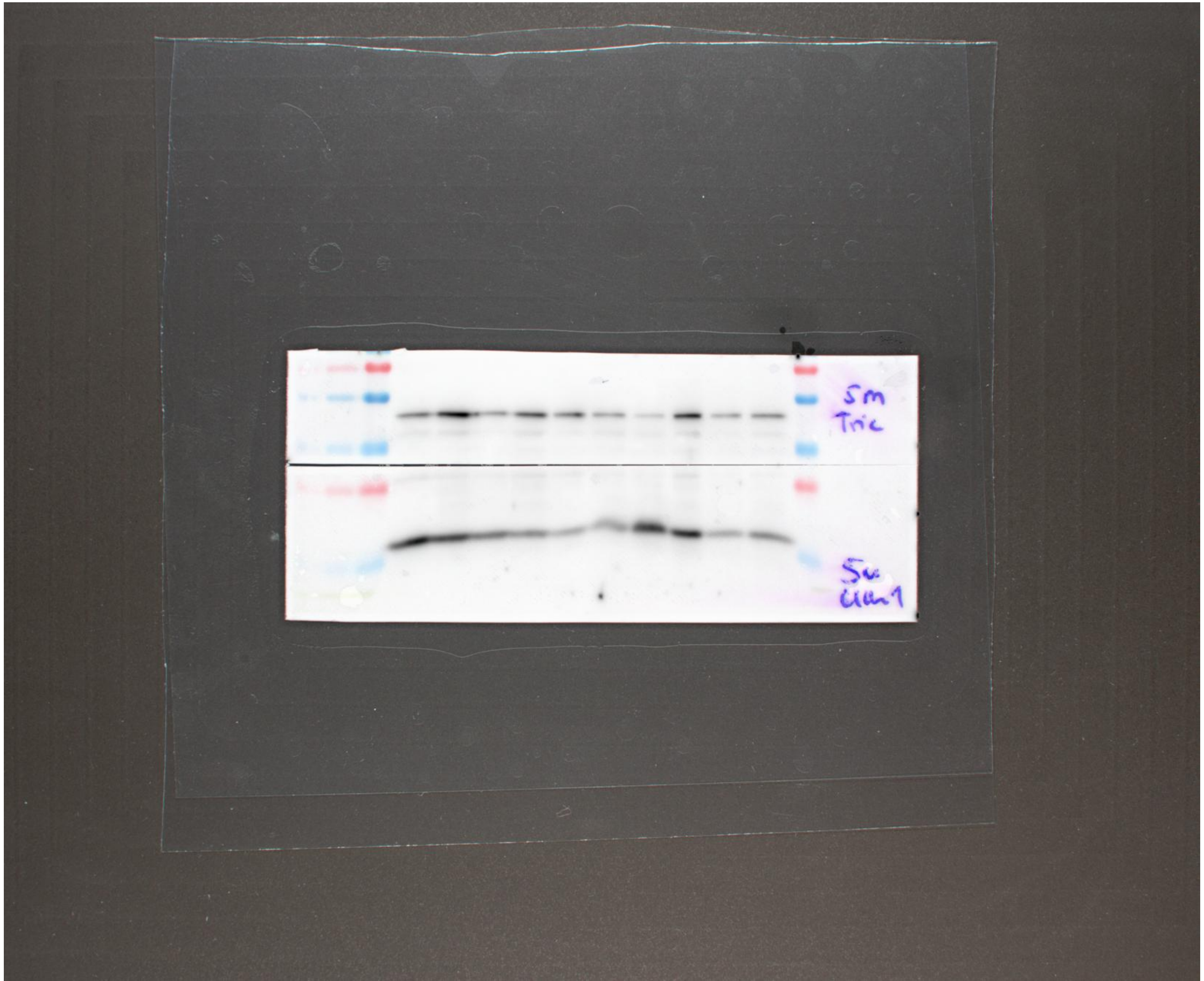

Fig. 4I-J: Cldn2

specific band

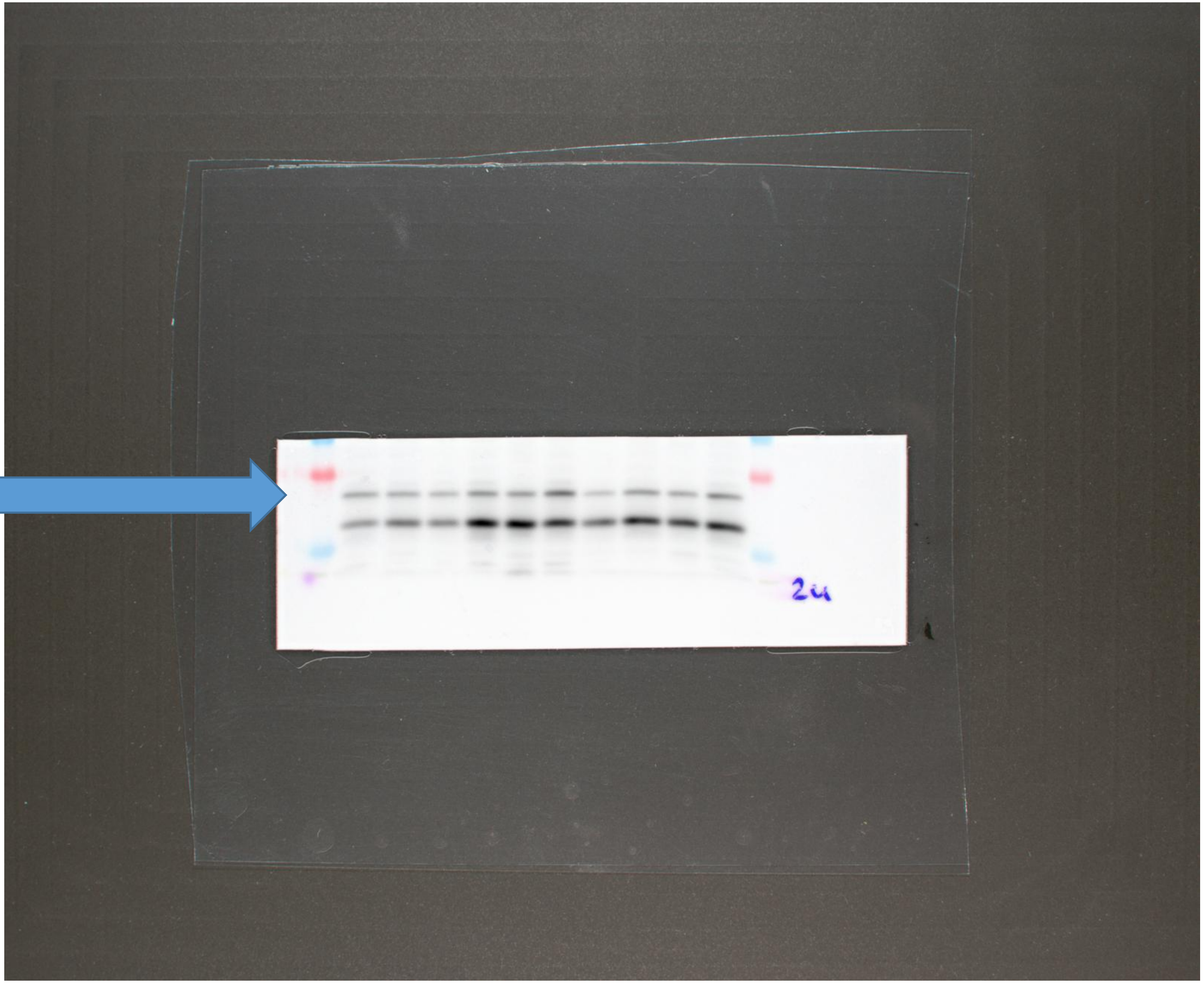

Fig. 4I-J: Cldn3

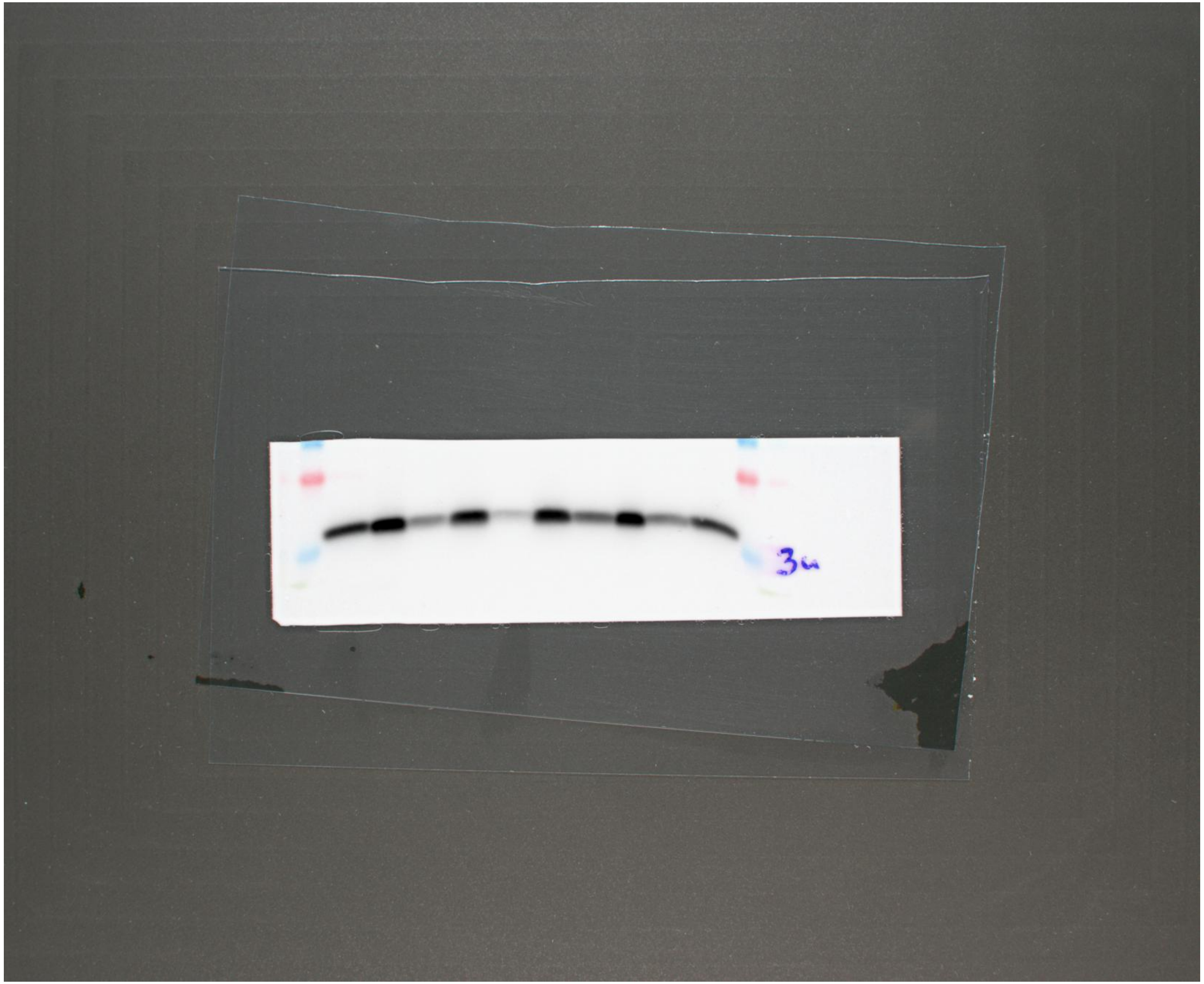

Fig. 4I-J: Cldn4

specific band

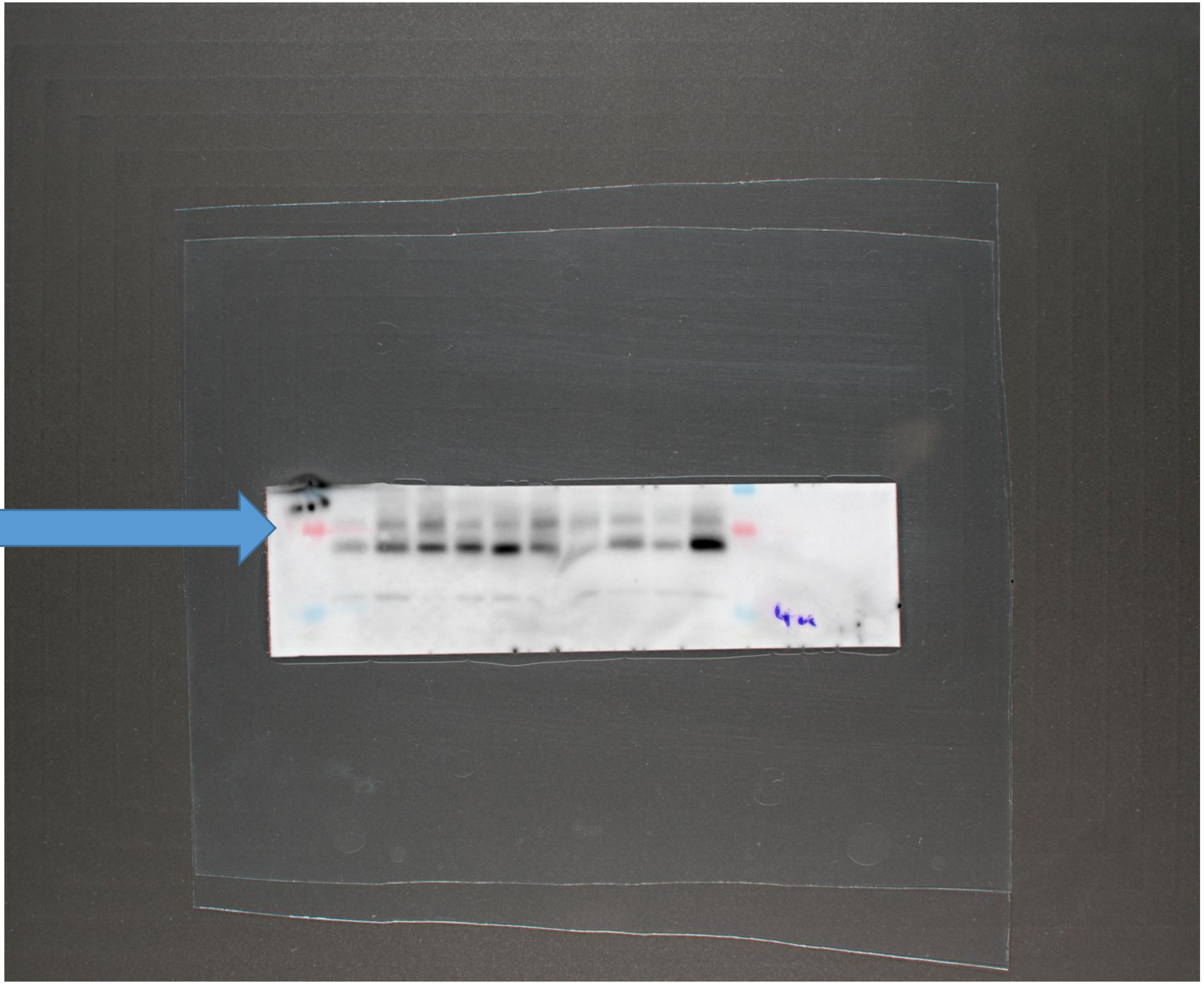

Fig. 4I-J: Cldn7

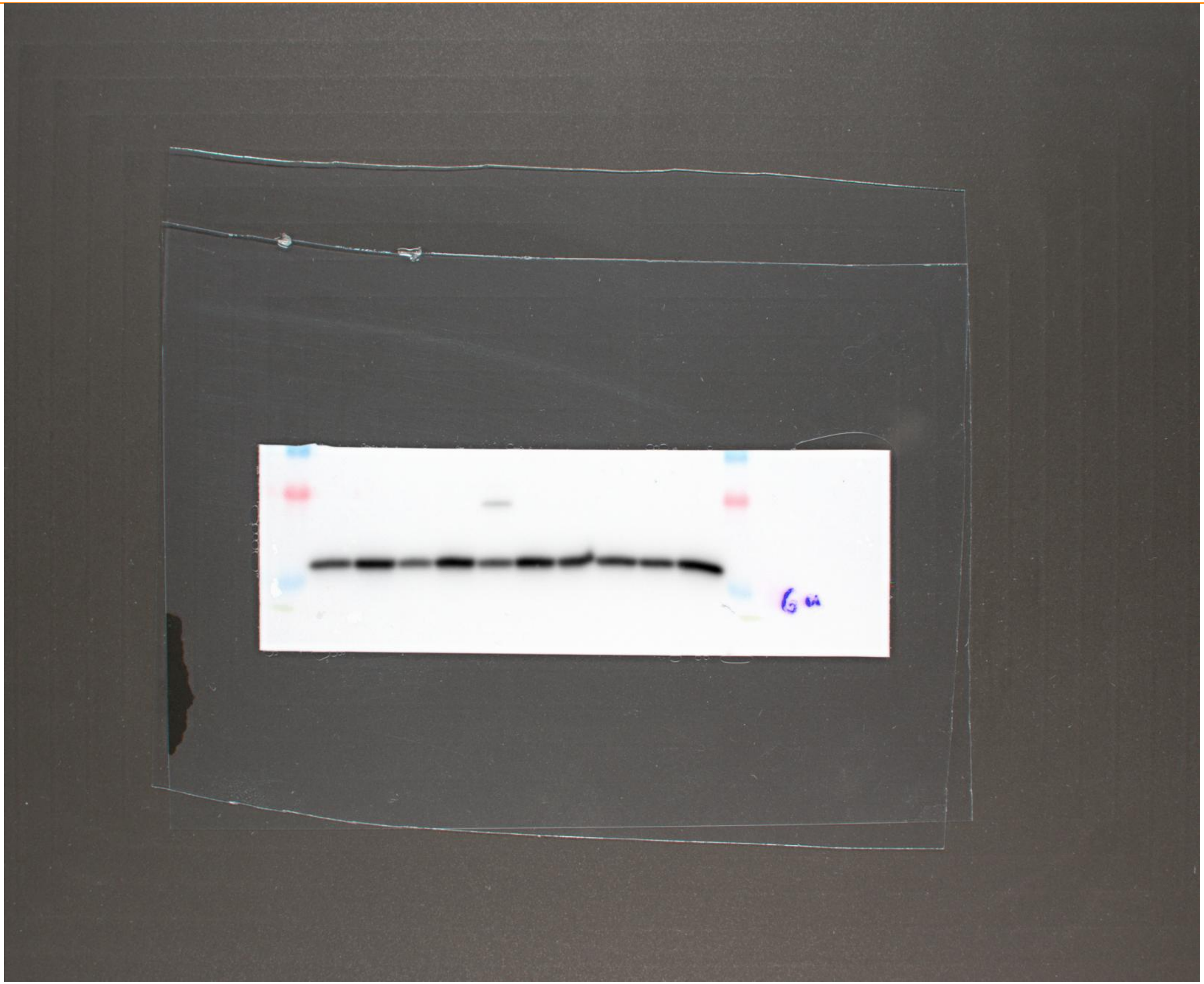

Fig. 4I-J: Occl

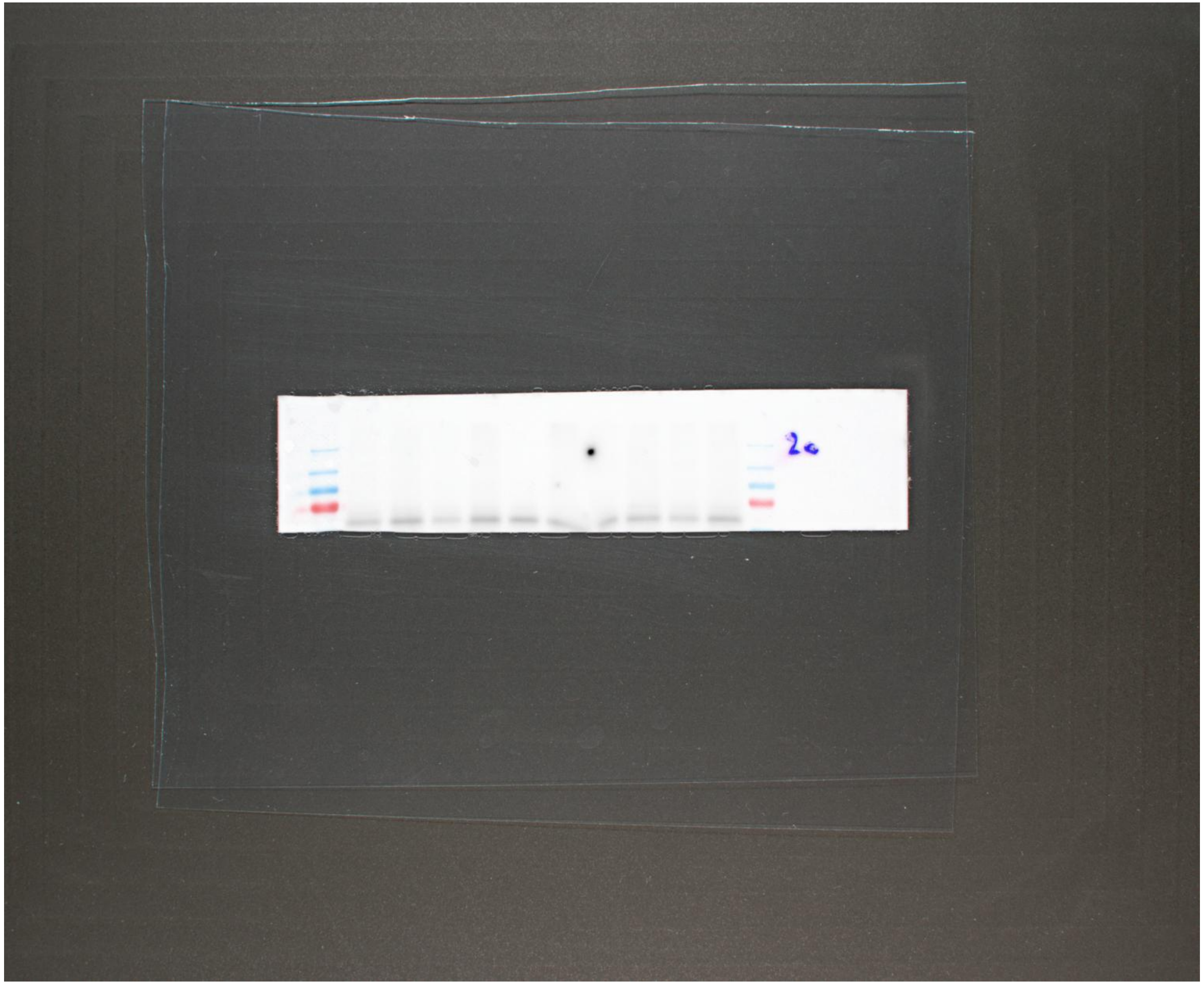

Fig. 4I-J: MD3

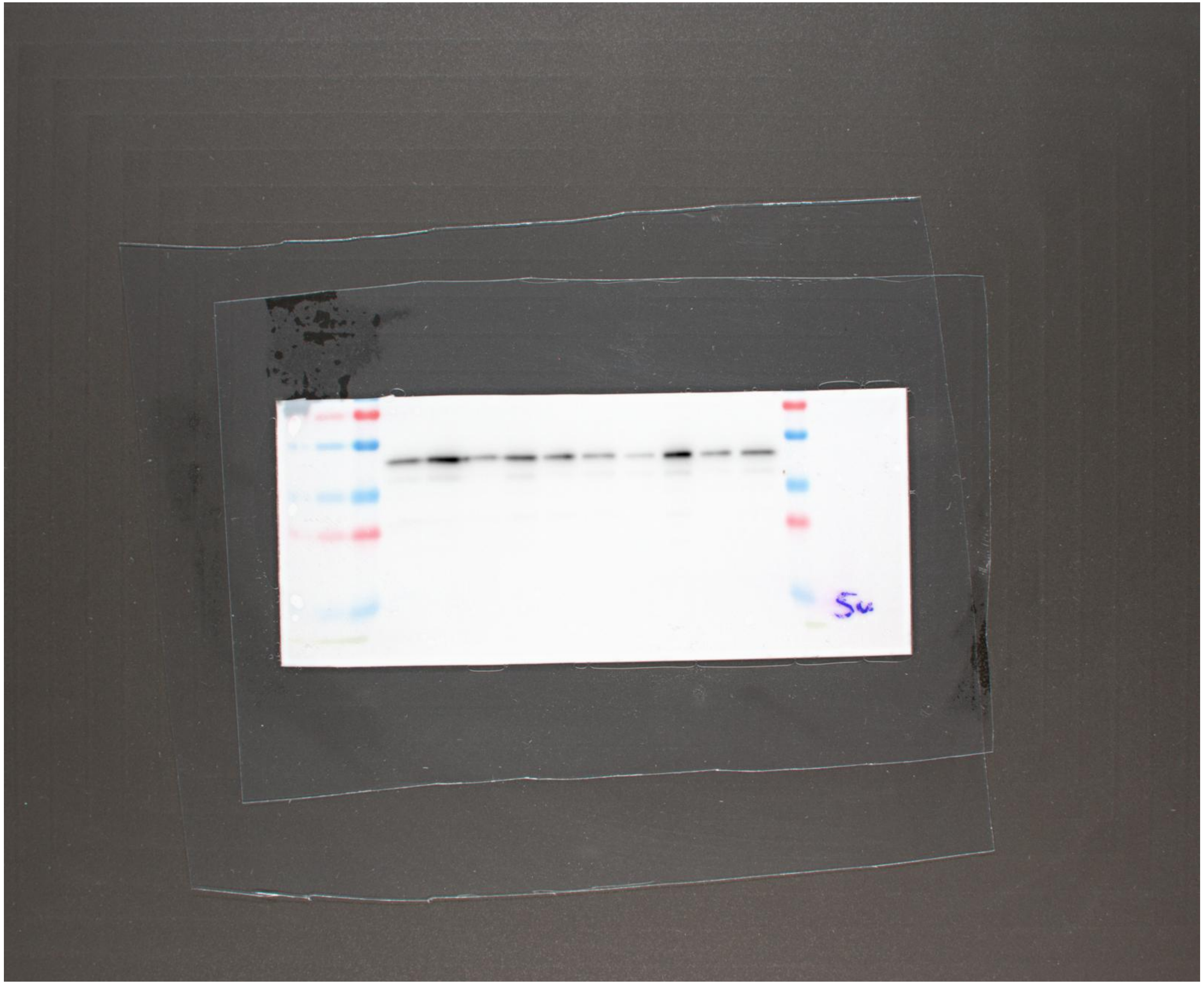

Fig. 4I-J: LSR

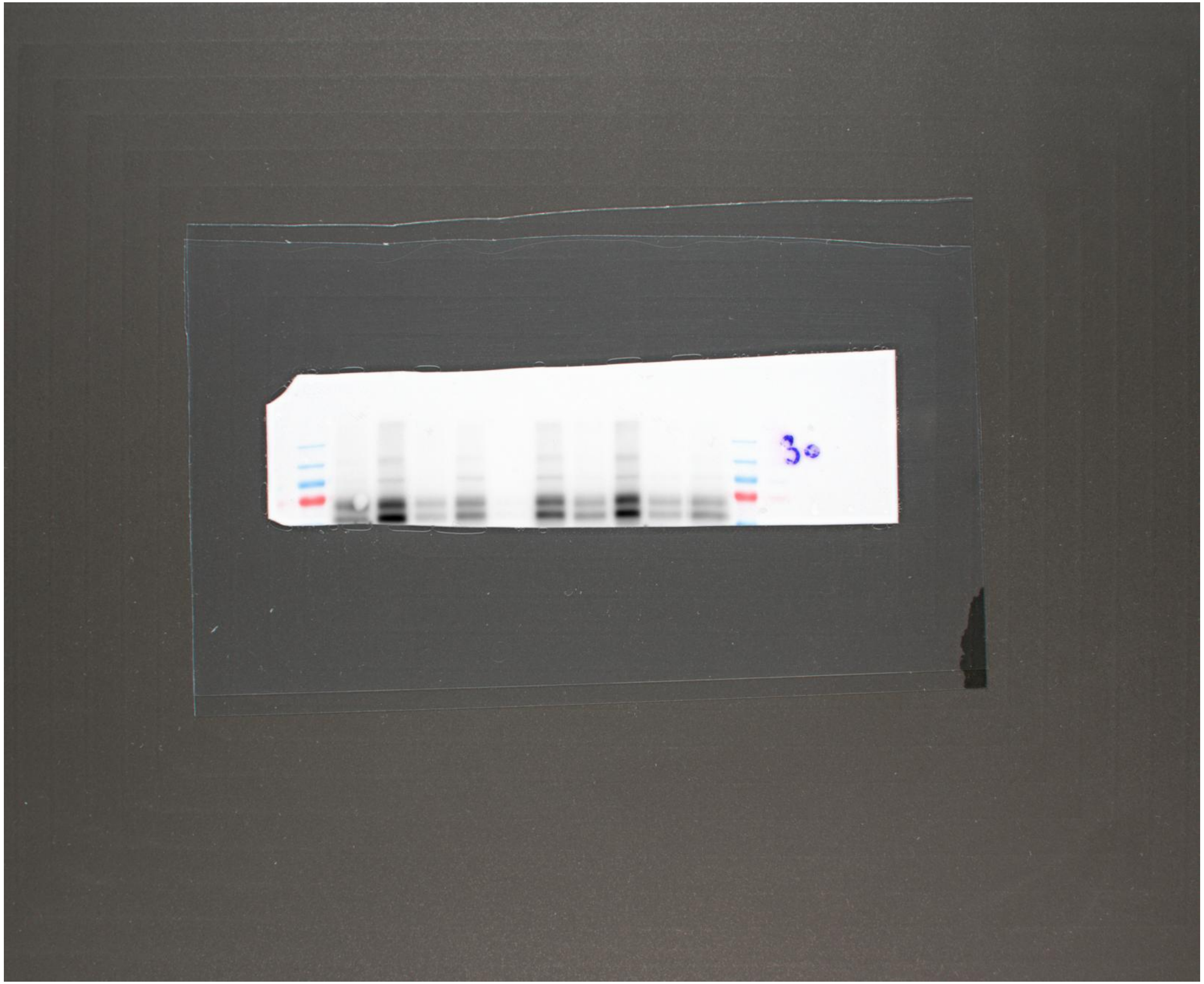

Fig. 4I-J: ZO-1

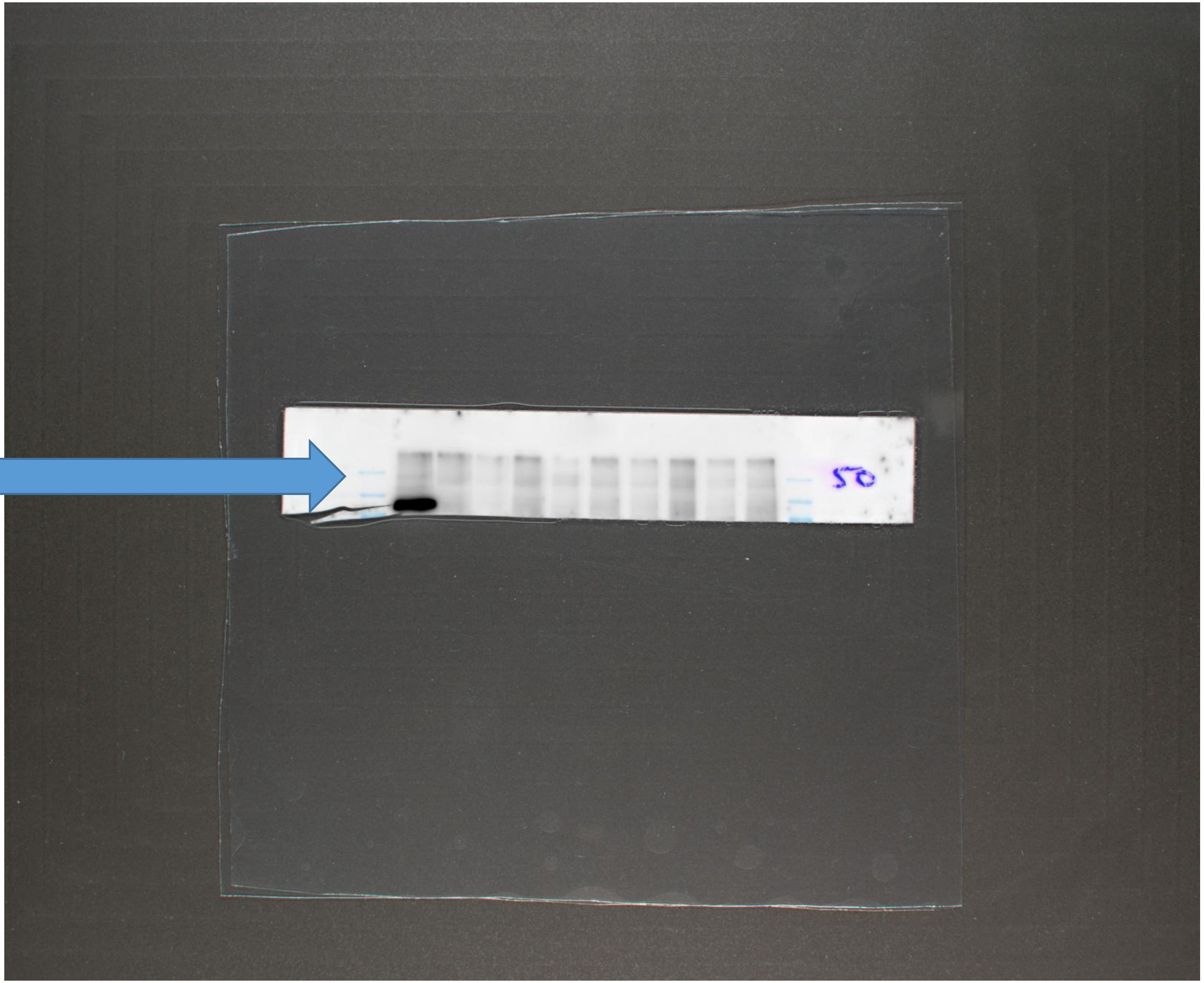

specific band

Fig. 4I-J: ILDR1

Isoform 1

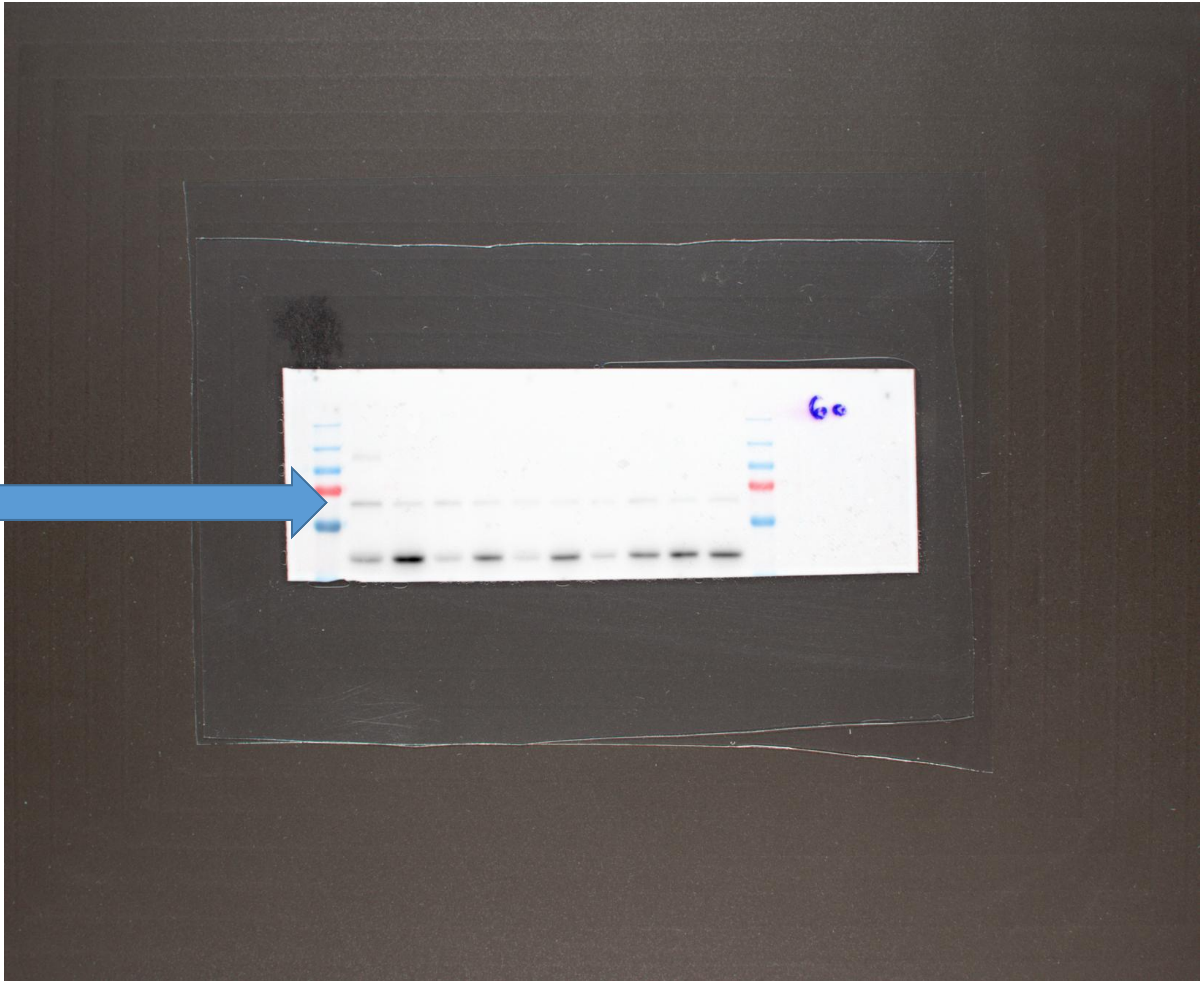

Fig. 4I-J: Tric

specific band

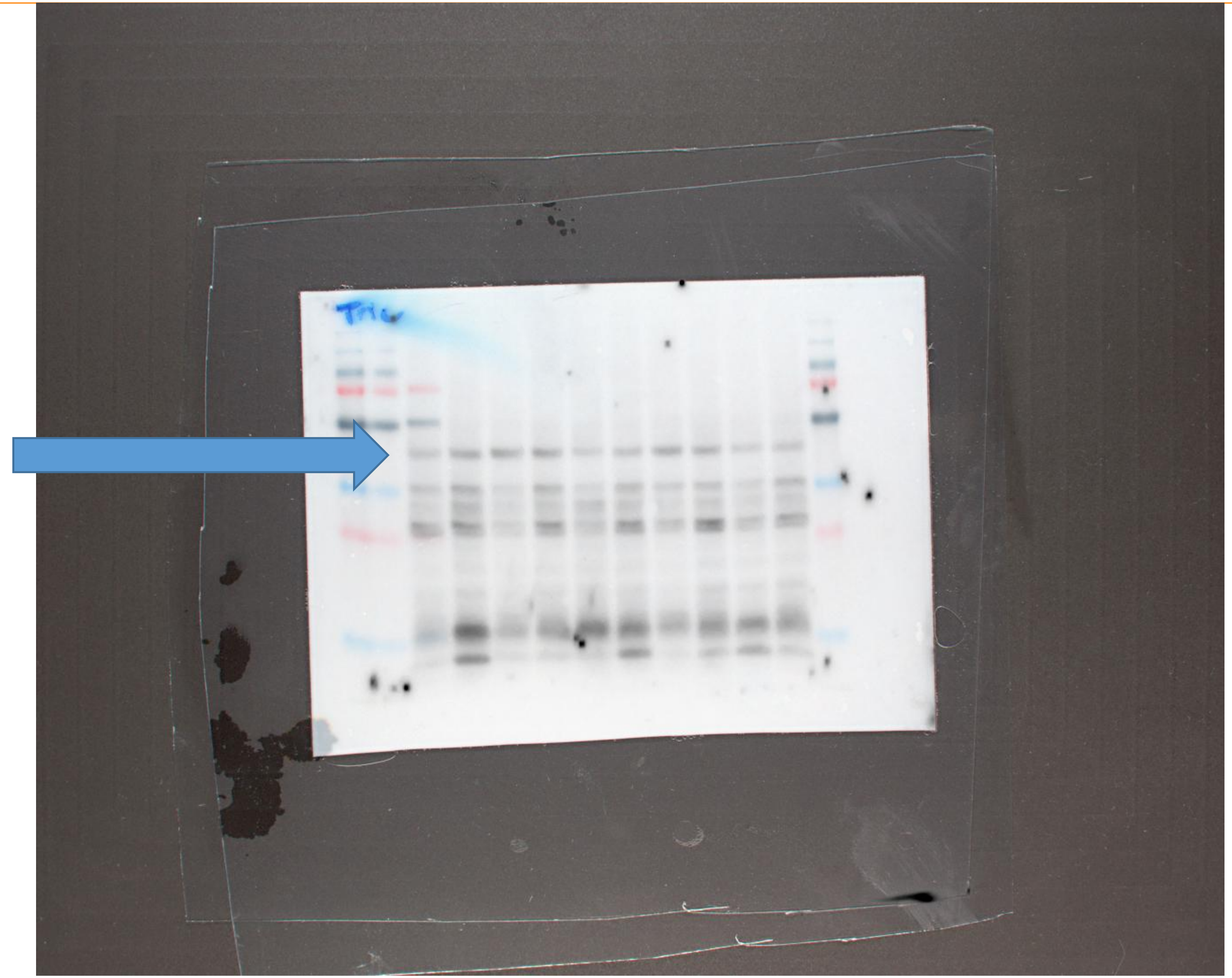

Fig. 4I-J: beta-Actin

Shown blot

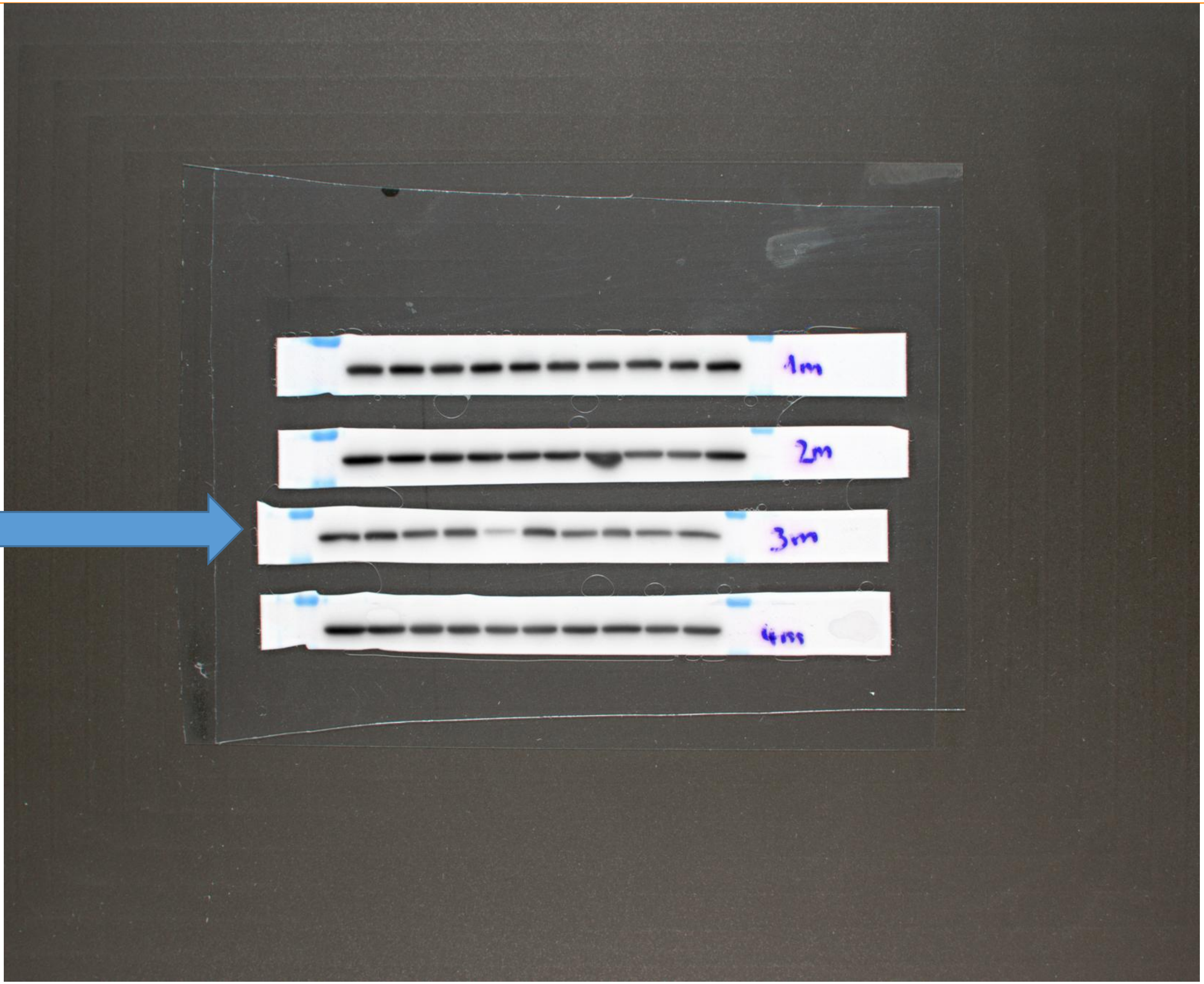

Suppl-Fig. 6E: Cldn3

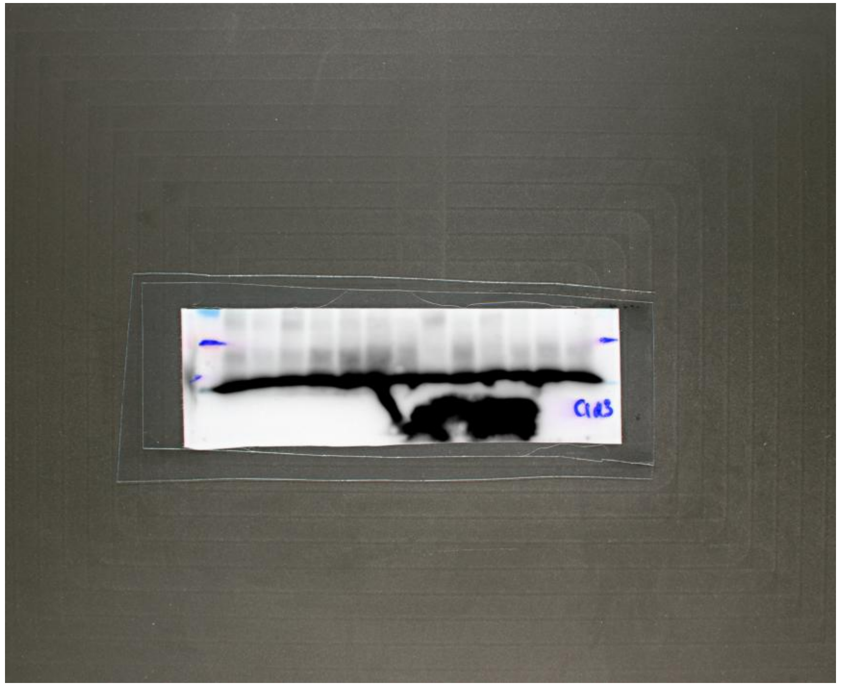

unspecific signals were covered as they were too bright (see above) and would overshine the specific signals when detection time was optimized for the region of interest

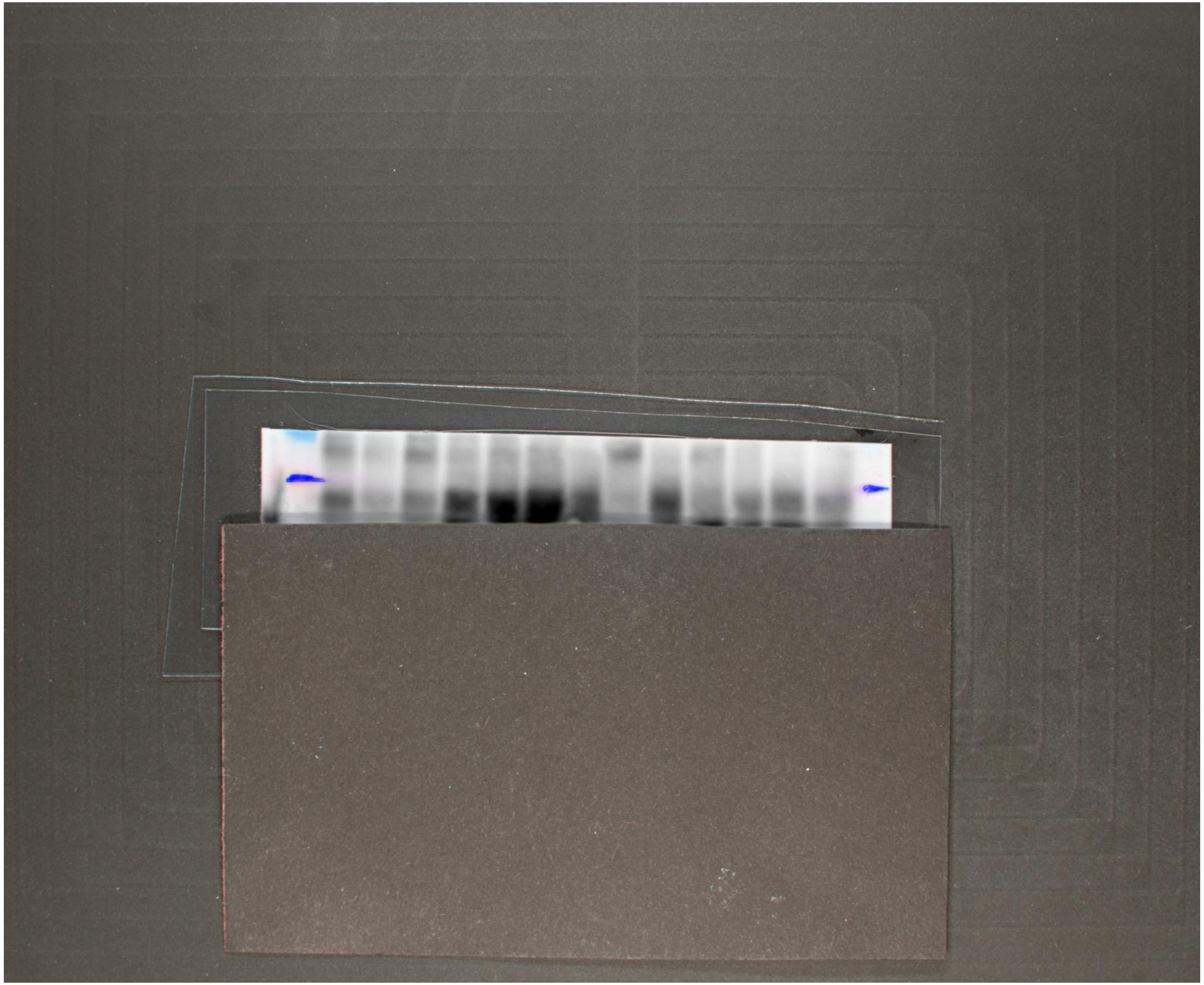

Suppl-Fig. 6E: LSR

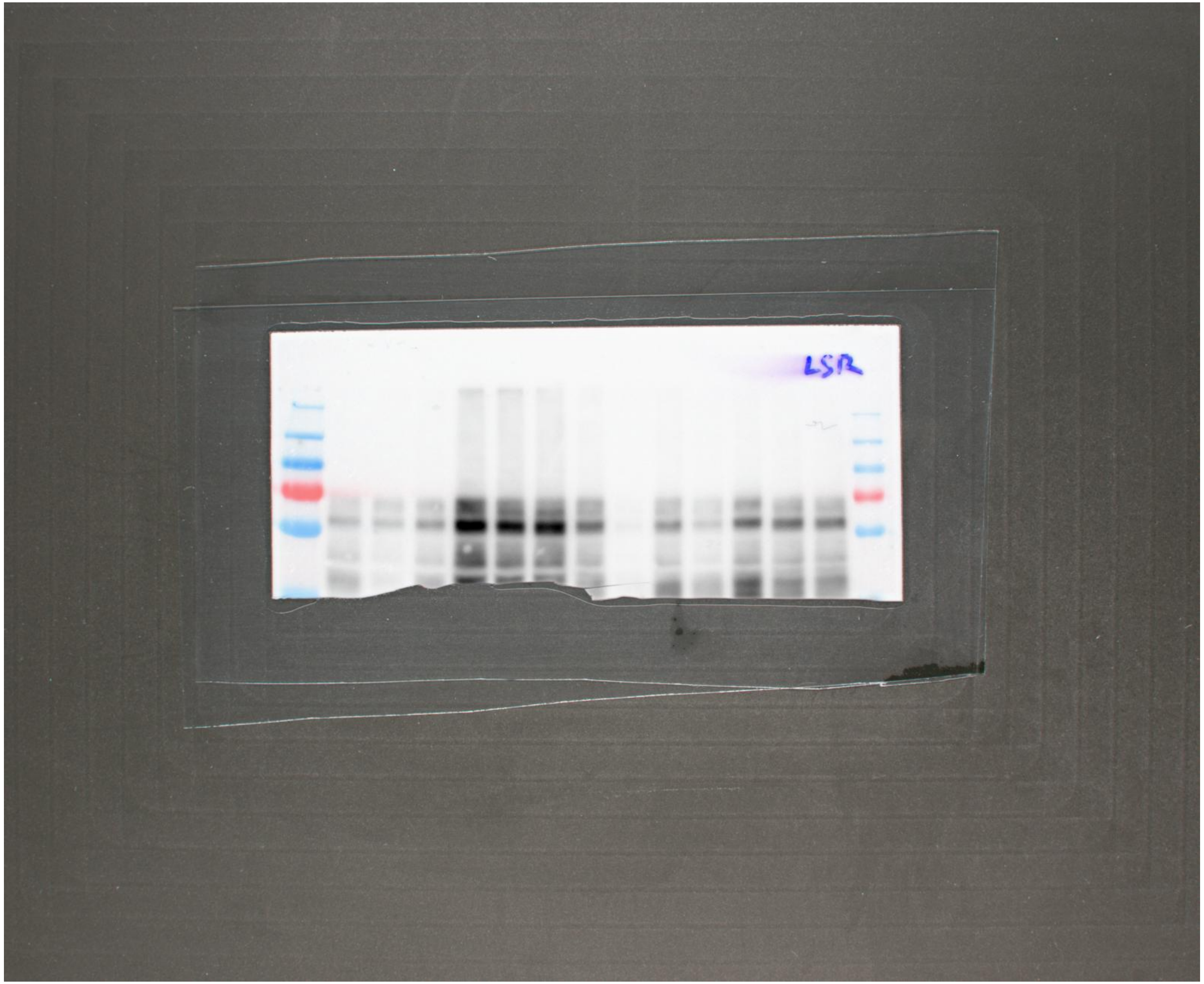

Suppl-Fig. 6E: Occludin

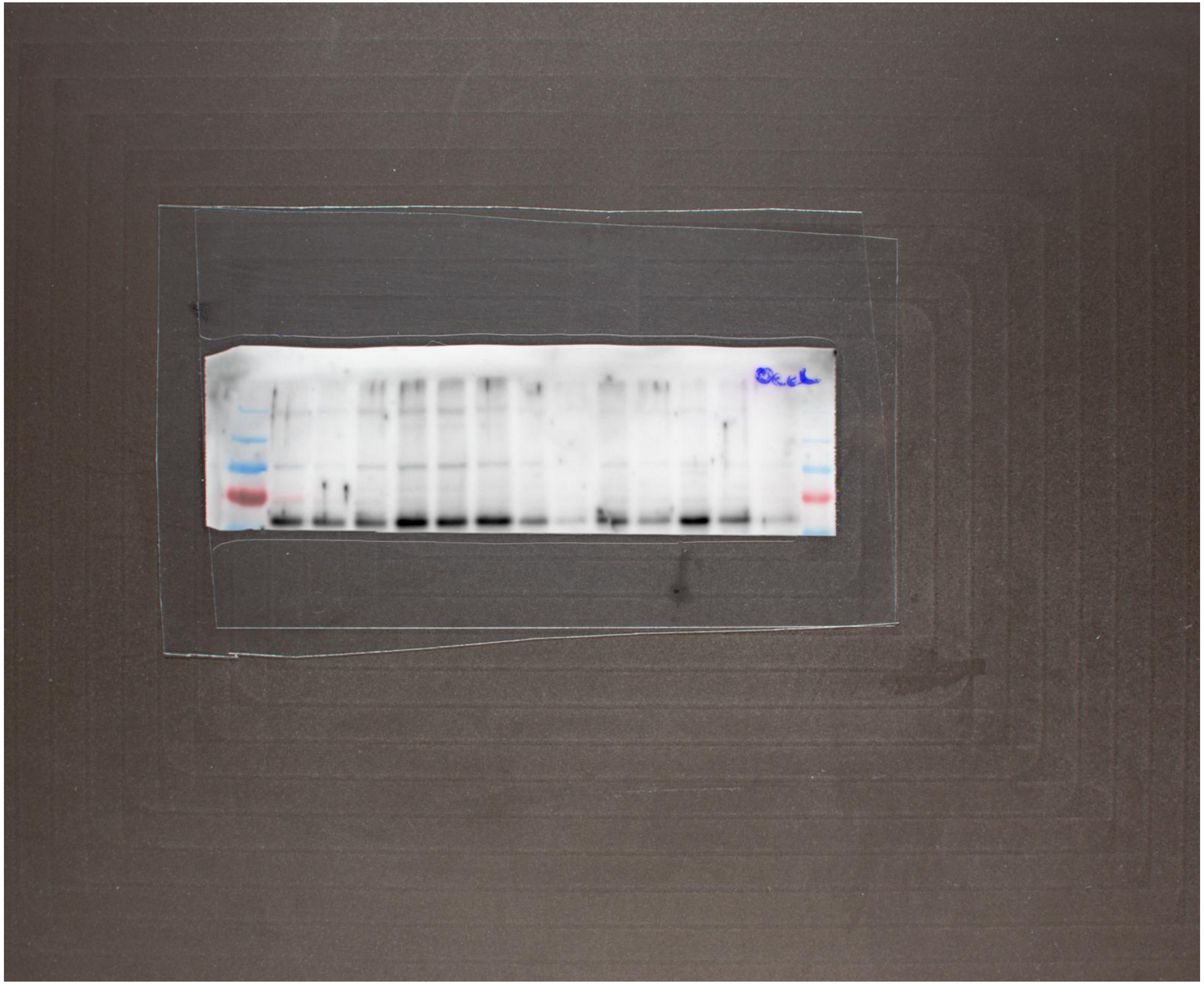

Suppl-Fig. 6E: beta-Actin

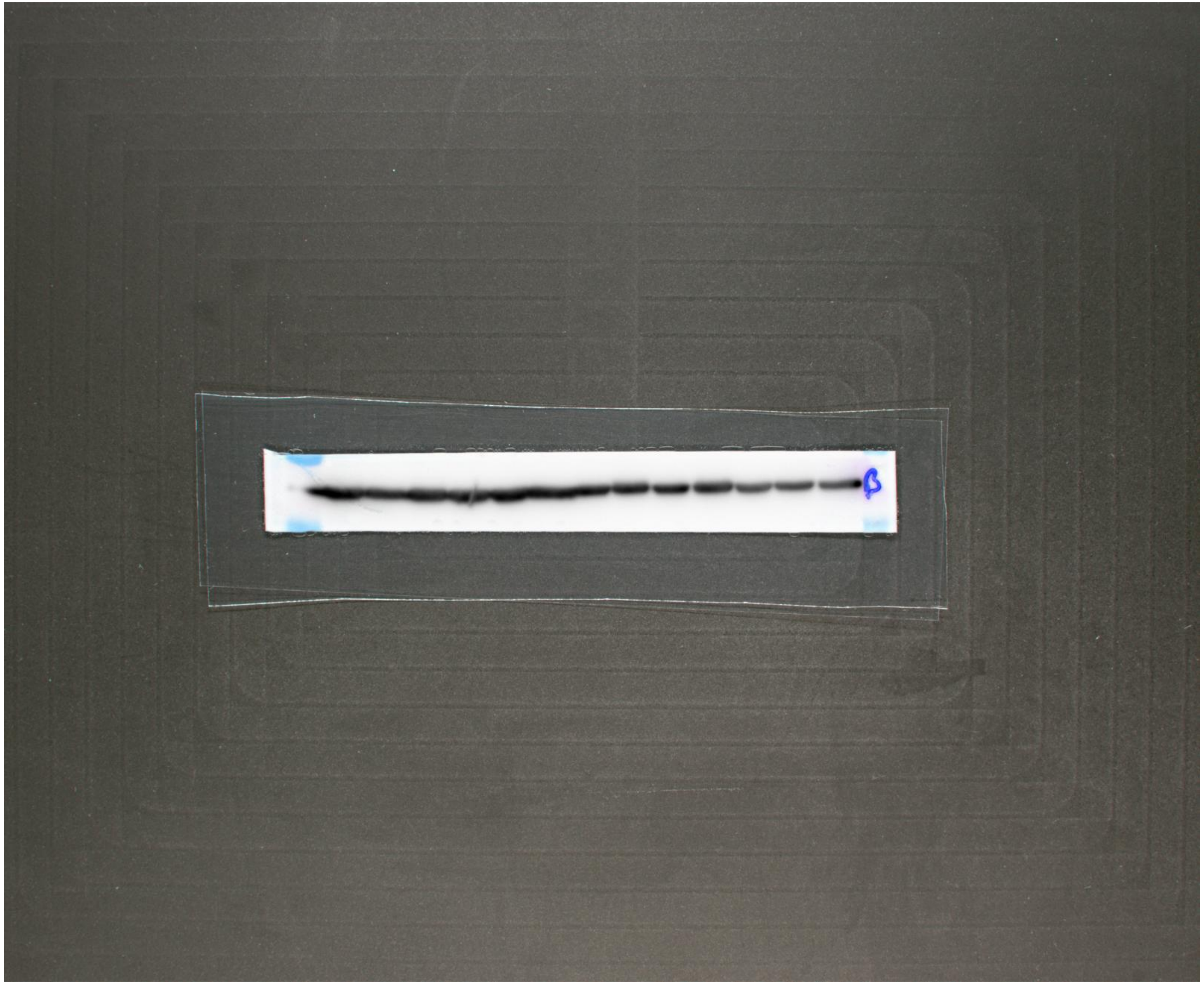

Supplement: Unedited blot and gel images [file jci-136-192322-s255.pdf]
